# Whole-exome sequencing identifies rare genetic variants associated with human plasma metabolites

**DOI:** 10.1016/j.ajhg.2022.04.009

**Published:** 2022-05-13

**Authors:** Lorenzo Bomba, Klaudia Walter, Qi Guo, Praveen Surendran, Kousik Kundu, Suraj Nongmaithem, Mohd Anisul Karim, Isobel D. Stewart, Claudia Langenberg, John Danesh, Emanuele Di Angelantonio, David J. Roberts, Willem H. Ouwehand, INTERVAL study, Ian Dunham, Adam S. Butterworth, Nicole Soranzo

**Affiliations:** 1 Wellcome Sanger Institute, Wellcome Genome Campus, Hinxton CB10 1SA, UK; 2 Open Targets, Wellcome Genome Campus, Hinxton CB10 1SD, UK; 3 British Heart Foundation Cardiovascular Epidemiology Unit, Department of Public Health and Primary Care, University of Cambridge, Cambridge CB1 8RN, UK; 4 Department of Haematology, University of Cambridge, Cambridge Biomedical Campus, Puddicombe Way, Cambridge CB2 0AW, UK; 5 MRC Epidemiology Unit, Institute of Metabolic Science, University of Cambridge, Cambridge CB2 0SL, UK; 6 Computational Medicine, Berlin Institute of Health at Charité – Utniversitätsmedizin Berlin, Berlin 10117, Germany; 7 British Heart Foundation Centre of Research Excellence, University of Cambridge, Cambridge CB2 0QQ, UK; 8 National Institute for Health Research Blood and Transplant Research Unit in Donor Health and Genomics, University of Cambridge, Cambridge CB1 8RN, UK; 9 Health Data Research UK Cambridge, Wellcome Genome Campus and University of Cambridge, Cambridge CB10 1SA, UK; 10 Human Technopole, Palazzo Italia, Viale Rita Levi-Montalcini 1, 20157 Milan, Italy; 11 NHS Blood and Transplant-Oxford Centre, Level 2, John Radcliffe Hospital, Oxford OX3 9BQ, UK; 12 Radcliffe Department of Medicine, University of Oxford, John Radcliffe Hospital, Oxford OX3 9BQ, UK; 13 European Molecular Biology Laboratory, European Bioinformatics Institute, Wellcome Genome Campus, Hinxton CB10 1SD, UK

**Keywords:** metabolon, rare genetic variant, metabolomics, loss-of-function, proteomics, endophenotypes, sequencing, WES, WGS, drug targets

## Abstract

Metabolite levels measured in the human population are endophenotypes for biological processes. We combined sequencing data for 3,924 (whole-exome sequencing, WES, discovery) and 2,805 (whole-genome sequencing, WGS, replication) donors from a prospective cohort of blood donors in England. We used multiple approaches to select and aggregate rare genetic variants (minor allele frequency [MAF] < 0.1%) in protein-coding regions and tested their associations with 995 metabolites measured in plasma by using ultra-high-performance liquid chromatography–tandem mass spectrometry. We identified 40 novel associations implicating rare coding variants (27 genes and 38 metabolites), of which 28 (15 genes and 28 metabolites) were replicated. We developed algorithms to prioritize putative driver variants at each locus and used mediation and Mendelian randomization analyses to test directionality at associations of metabolite and protein levels at the *ACY1* locus. Overall, 66% of reported associations implicate gene targets of approved drugs or bioactive drug-like compounds, contributing to drug targets' validating efforts.

## Graphical abstract

**Figure:**
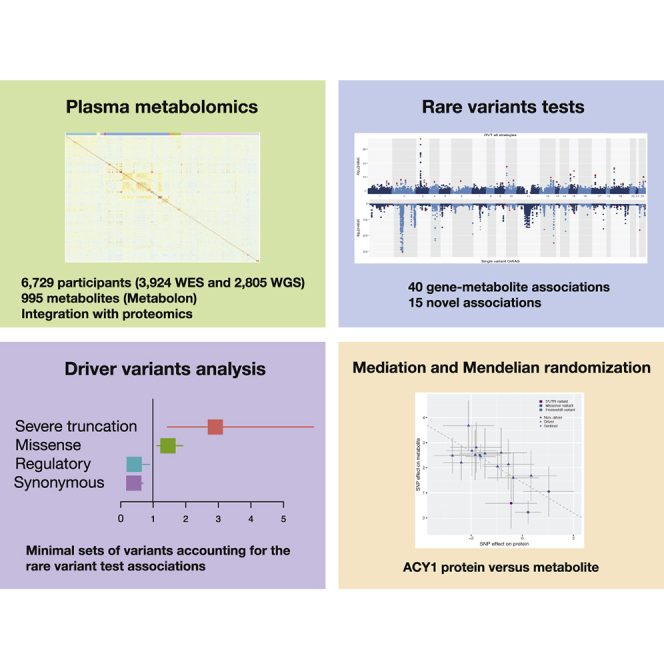

## Introduction

Variability of metabolite levels in the human population is influenced by both extrinsic and intrinsic factors. Genetic variation can affect metabolite levels by regulating the expression of enzyme-coding genes, modifying the structure of the enzyme or completely inactivating the enzyme in the case of protein-truncating variants. This could lead to a disruption of a particular metabolic pathway and, depending on the severity of this disruption, to the development of disease.^1^ Metabolites are intermediate phenotypes between genes and clinical outcomes, and thus studying metabolites can aid the interpretation of effector genes of genome-wide association studies (GWASs) of complex traits and diseases. Genetic variants associated with metabolites are enriched near genes of pharmacological interest, aiding the evaluation of potential drug targets.^2^ Often, genes involved in inborn errors of metabolism also harbor genetic variants associated with metabolite levels related to the disorder, and those same genetic variants may also be associated with complex traits and diseases.^2^

GWASs have identified hundreds of common (minor allele frequency [MAF] > 0.1%) genetic variants associated with metabolite levels.^2^^,^^3^ Relatively less is known about the contribution to metabolites across the rare spectrum of genetic variation.^4^, ^5^, ^6^ Studying variants with a predicted severe impact on proxies of protein function can inform drug discovery efforts^7^ by aiding the interpretation of the phenotypic consequences of partial or complete gene “knockouts” in humans. A genetic variant that inactivates a gene encoding a drug target may mimic the pharmacological modulation of a drug, providing an “experiment of nature” to inform drug development. Metabolite levels associated with such variants could be used as readouts to infer clinical and therapeutic effects of a drug.

To expand our knowledge of rare high-impact (i.e., predicted loss-of-function and missense) variants associated with levels of nearly 1,000 plasma metabolites, we interrogated a cohort of apparently healthy blood donors recruited in the INTERVAL study^8^ with whole-exome sequencing (WES) or whole-genome sequencing (WGS). We used a robust approach to aggregate loss-of-function and/or missense rare variants in test windows to improve the statistical power to detect signals. For comparison, we also explored synonymous variants with no predicted functional impact. Moreover, we developed a novel method to assess the contributions of individual rare variants to the aggregated association signals. We also investigated whether any of the genes found to be associated with metabolite levels were also associated with protein levels measured in the same cohort. Overall, this study demonstrates the value of a densely phenotyped cohort for dissecting changes associated with metabolite and protein levels.

## Subjects and methods

### Study description

The INTERVAL study^8^ comprises approximately 45,000 apparently healthy blood donors nested within a randomized trial of blood donation intervals. The trial has received ethics committee approval from the National Research Ethics Service Committee East of England - Cambridge East (Research Ethics Committee [REC] reference 11/EE/0538). Between mid-2012 and mid-2014, whole-blood donors aged 18 years and older were consented and recruited at 25 centers of England’s National Health Service Blood and Transplant. All participants completed an online questionnaire including questions about demographic characteristics (e.g., age, sex, ethnic group), anthropometry (height, weight), lifestyle (e.g., alcohol and tobacco consumption), and diet. Participants were generally in good health because blood donation criteria exclude people with a history of major disease (such as myocardial infarction, stroke, cancer, HIV, and hepatitis B or C) and those who have had recent illness or infection. Study participants were randomly selected into two non-overlapping sub-cohorts of 4,502 and 3,762 participants, which were both screened with the Metabolon platform. The former sub-cohort was whole-exome sequenced, while the latter was whole-genome sequenced. Both were genotyped with the UK Biobank Axiom Array and imputed with a combined UK10K-1000G Phase III imputation panel.

### Metabolite measurements

The non-targeted metabolomics analysis was performed at Metabolon (Durham, North Carolina, USA) on a platform consisting of four independent ultra-high-performance liquid chromatography–tandem mass spectrometry (UPLC–MS/MS) instruments.

Raw data were extracted, peaks identified, and quality control (QC) processed via Metabolon’s hardware and software. Compounds were identified by comparison to library entries of purified standards or recurrent unknown entities. Metabolon maintains a library based on authenticated standards that contains the retention time/index (RI), mass-to-charge ratio (m/z), and chromatographic data (including MS/MS spectral data) on all molecules present in the library. Furthermore, biochemical identifications are based on three criteria: retention index within a narrow RI window of the proposed identification, accurate mass match to the library ±10 ppm, and MS/MS forward and reverse scores between the experimental data and authentic standards. MS/MS scores are based on a comparison of the ions present in the experimental spectrum to the ions present in the library spectrum. While there may be similarities between these molecules based on one of these factors, the use of all three data points can distinguish and differentiate biochemicals. More than 3,300 commercially available purified standard compounds have been acquired and registered into LIMS for analysis on all platforms for determination of their analytical characteristics. Additional mass spectral entries have been created for structurally unnamed biochemicals, which have been identified by virtue of their recurrent nature (both chromatographic and mass spectral). These compounds have the potential to be identified by future acquisition of a matching purified standard or by classical structural analysis.

A variety of curation procedures were carried out to ensure that a high-quality dataset was made available for statistical analysis and data interpretation. The quality control and curation processes were designed to ensure accurate and consistent identification of true chemical entities and to remove those representing system artifacts, misassignments, and background noise. Metabolon data analysts used proprietary visualization and interpretation software to confirm the consistency of peak identification among the various samples. Library matches for each compound were checked for each sample and corrected if necessary.

### Quality control of metabolites

Plasma samples from 8,536 INTERVAL participants that passed WES QC (Table S1) were sent to Metabolon for metabolite profiling. Plasma samples were sent in two batches and were thus processed at different times. Because of the potential for batch effects, QC of the metabolite data was done by batch. Metabolites with >100 missing values were excluded, and in total, 995 metabolites were available after QC. Metabolites were log-transformed by taking the natural logarithm. A metabolite value was defined as an outlier and winsorized where the value was 5 or more standard deviations away from the mean metabolite value. A principal-component analysis (PCA) was done with nonlinear iterative partial least squares because of the sparsity of the dataset. To help identify any multivariate outliers, we performed biplots comparing the first principal component to each of the next nine principal components. We undertook linear regressions of the first five principal components against age, sex, BMI, current smoking, alcohol consumption frequency, center, batch, plate (as a proxy for run day), appointment month (proxy for possible seasonal effects), and time between appointments (when blood samples were taken) and processing (measured in two ways because of varying levels of missing data: as hours and by days) to determine whether these factors were significantly associated with variability of the metabolites. As shown in Table S2, age, sex, BMI, current smoking, alcohol, INTERVAL center, plate, appointment month, and lag time between appointment and processing account for some of the variability in the metabolites. Adjusting the metabolite levels for some of these variables may be appropriate, however as a result of high levels of missingness, not all variables were considered. For this reason, log-transformed and winsorized metabolite values were adjusted in a linear regression only for age, sex, BMI, center, batch, plate, appointment month, time between appointment and processing, and the first five principal components of ancestry from multi-dimensional scaling. The metabolite residuals from this linear regression were then rank-inverse normalized and used as phenotype for association testing. To give a more detailed description of the covariates: age was calculated as the participant’s age (in years) at the time at which the blood sample was collected. BMI was estimated as self-reported weight (in kilograms) divided by the square of self-reported height (in meters). Current smoking was assigned on the basis of the information provided by the participant about their tobacco smoking status at baseline, with adjustment for “current smoker” versus “never” or “former” smoker combined. More specifically, current smoking was based on participants who responded “yes” to the question "Do you currently smoke?" while never/former smoking status was defined as those who responded “no” to that question and responded either “yes” (former) or “no” (never) to the question "Have you ever smoked?" Alcohol consumption frequency was also collected by self-report in the baseline questionnaire. Appointment month was taken as the month of the year in which the blood sample was collected and was used as a 12-factor categorical variable to account for potential seasonal effects. Finally, we also adjusted for the length of time between the blood collection (as proxied by the time of the appointment at the recruitment center) and the time at which the blood sample was fractionated at the processing laboratory after it had been shipped from the recruitment center. For the majority of samples (>95%), the processing happened the next day, so we used a binary variable (1 day versus >1 day) to account for those samples for which samples were not processed the following day. To account for more subtle effects, we also adjusted for the number of hours between the sample collection and sample processing as a continuous variable.

A total of 230 metabolic biomarkers were produced by the serum nuclear magnetic resonance (NMR) metabolomics platform (Nightingale Health)^9^ on 46,097 samples in the INTERVAL cohort. Glucose, lactose, pyruvate, and acetate were excluded initially because of unreliable measurements. Conjugated linoleic acid and conjugated linoleic acid to total fatty acid ratio were set to missing for 3,585 samples showing signs of peroxidation. Creatinine levels were set to missing for 1,993 samples with isopropyl alcohol signals. Glutamine levels were set to missing for 347 samples that showed signs of glutamine to glutamate degradation. Samples with more than 30% missingness or identified as EDTA plasma were removed.

### Protein measurements and quality control

We used a multiplexed, aptamer-based approach (SOMAscan assay) to measure the relative concentrations of 3,622 plasma proteins or protein complexes assayed via 4,034 modified aptamers in plasma. The proteins cover a wide range of molecular functions. Details of the protein measurements have been described previously.^10^ After quality control and excluding samples with missing protein measurements, 3,301 participants overlapping with WES data remained for analysis.

### Sequencing and quality control

WES and WGS were performed at the Wellcome Sanger Institute (WSI) sequencing facility.

For WES, sheared DNA was prepared for Illumina paired-end sequencing and enriched for target regions with Agilent's SureSelect Human All Exon V5 capture technology (Agilent Technologies; Santa Clara, California, USA). The exome-captured library preparation was sequenced with the Illumina HiSeq platform as paired-end 75 bp reads, reaching an average depth of approximately 50×. Reads were aligned to the GRCh37 human reference genome wiyh BWA (v0.5.10).^11^ GATK HaplotypeCaller v3.4 ^12^ was used for variant calling and recalibration. Samples were excluded on the basis of the following criteria: (1) withdrawn consent; (2) estimated contamination >3% according to the software VerifyBamID;^13^ (3) sex inferred from genetic data different from sex supplied; (4) non-European samples after manual inspection of clustering in 1000G PCA and choosing cutoffs on the first two PCs; (5) heterozygosity outliers (samples +/− 3 SDs away from the mean number of heterozygous counts); (6) non-reference homozygosity outliers (samples +/− 3 SDs away from the mean number of non-reference homozygous counts); (7) outlier Ti/Tv ratio (transition to transversion ratio +/− 3 SDs away from the mean ratio); and (8) excess singletons (number of singleton variants >3 SDs from the cohort mean). After QC, 4,070 samples were kept in the final release. Genetic variants with MAF > 1% were excluded with the following thresholds: (1) variant quality score recalibration (VQSR): 99.90% tranche; (2) missingness > 3%; and (3) Hardy Weinberg Equilibrium (HWE) p < 1 × 10^–5^. Genetic variants with MAF ≤ 1% were excluded with the following thresholds: (1) VQSR: 99.90% tranche; (2) genotype quality (GQ): <20 for SNPs and <60 for Indels; (3) sequencing depth (DP) < 2; and (4) allelic balance (AB) > 15 and < 80 for heterozygous variants. After genotype-level QC (GQ, DP, AB), only variants with <3% missingness were kept. A total of 1,716,946 variants were kept in the final release. Out of the 4,070 samples passing the QC, metabolite data were available for 3,924.

For WGS, sheared DNA was prepared for Illumina paired-end sequencing. Sequencing was performed with the Illumina HiSeq X platform as paired-end 75 bp reads, reaching an average depth of 15×. Reads were aligned to the GRCh38 human reference genome with mostly BWA (v.0.7.12) although a subset of samples was aligned with v.0.7.13 or v.0.7.15. GATK HaplotypeCaller v3.5 was used for variant calling and recalibration. We extracted coordinates from the VCF files that mapped to regions targeted in the WES. We then used custom scripts to transform coordinates of variants to the GRCh37 human reference. We filtered out samples on the basis of the following criteria: (1) estimated contamination > 2% according to the software VerifyBamID; (2) non-reference discordance (NRD) with genotype data on the same samples >4%; (3) population outliers from PCA (PC1 > 0 and minimum PC2); (4) heterozygosity outliers (samples +/− 3 SDs away from the mean number of heterozygous counts); (5) number of third-degree relatives (proportion IBD [PI-HAT)] > 0.125) > 18; and (6) overlap with WES. After quality control, 3,670 WGS samples were kept. Out of the 3,670 samples passing the QC, metabolite data were available for 2,805.

All the genetic variants reported in the text and in the tables were lifted to GRCh38.

### Single-variant association test

Single-variant association tests were performed for each variant (all QC’ed whole-exome sequence variants) via an additive genetic model for all 995 metabolites. The association tests were carried out with RAREMETALWORKER v4.14.^14^ The analysis software returns the summary statistics for each variant and each specific metabolite and a covariance matrix that reports the pairwise LD of variants in 1 MB regions. These statistics were subsequently used in RAREMETAL to perform rare variant aggregation tests as described in the next section. Genomic control values ranged from 0.98 to 1.04, indicating no substantial inflation or deflation due to population stratification.

### Rare-variant aggregation tests

We used a total of four different rare-variant tests (RVTs) to investigate the aggregated effect of multiple rare variants with MAF < 0.1% on each trait, exploring two types of allelic architecture: (1) we used burden family tests, such as burden test, Madsen and Browning (MB), and variable threshold (VT), to discover signals where variants with the same direction and magnitude of effects were tested together. They mostly vary on how they use weighted and unweighted functions with a fixed or variable frequency threshold. (2) SKAT is a variance-component multiple regression test that retains power in settings where neutral variants or variants with opposite direction of effects could result in loss of power. For the rare variant analyses, we used RAREMETAL v4.14,^14^ which allows us to perform RVT by using single-variant test statistics and their correlations.

One of the biggest challenges of rare variant aggregation is to define sets of variants that identify domains encoding for a biological function. Our selection included all rare variants within coding exons, splice sites, or UTR regions of known genes (52,912 genes on autosomes) based on GENCODE v24 lifted over to build 37, only some types of pseudogenes (IG_C, IG_J, IG_V, TR_J, TR_V) were removed. Overlapping exons (528,874 exons) were merged within each gene, resulting in 301,736 exonic regions. Windows were generated by keeping the exon structure intact as far as possible and allowing between ∼5 and ∼20 variants per window. If there were less than five variants within an exon or more than 20 variants per gene, then windows were created by combining neighboring exons so that the number of variants was similar between windows. More specifically, the algorithm procedure is as follows. (1) If the first or last exon has fewer than five variants, the variants in this exon are combined with the variants in the neighboring exon. (2) If there is still exactly one exon with fewer than five variants, it is combined with its neighbor. (3) If an exon has more than 20 variants, it is split into roughly equal numbers of variants (e.g., 21 variants will be split into 11 and 10 variants). (4) The overall number of variants is calculated for each gene and the number of windows needed. (5) Then the average number of variants per window is calculated as a target so that the variants can be split equally between windows. (6) The number of variants per window is computed iteratively by adding the number of variants for each exon, starting with the first exon. (7) The optimal distribution is the one where the number of variants per window has the minimum difference to the target number.

Three different strategies in selecting variants were used. (1) CODING tests of all rare exonic variants, splice sites, and variants residing in UTRs. In the CODING approach, we tested 23,864 genes in 52,024 windows with 15 variants per window on average and a minimum of five and a maximum of 30 variants. (2) MLOF tests of LoF and missense variants combined; In total we analyzed 20,835 genes in 32,534 windows with 14 variants per window on average, and at least 5 variants and maximal 28 variants per window. iii) LOF variant tests; In the LOF approach we tested only LoF variants within each window predicted as high confidence (HC) by LOFTEE. LOFTEE (loss-of-function transcript effect estimator) is a plugin to the Variant Effect Predictor (VEP) that considers all stop-gained, splice-disrupting, and frameshift variants and filters out many known false-positive modes, such as variants near the end of transcripts and in non-canonical splice sites, as described in the code documentation. In total, we analyzed 9,385 genes in 9,428 windows with three variants per window on average and minimum two and maximum 19 variants per window. The distribution of the number of variants per window in each method are reported in Figure S1.

Multiple correction testing was performed with false discovery rate (FDR). We included p values tested in all analysis approaches to calculate q values by using the core R package function “p.adjust,” that implements the Benjamini and Hochberg (1995) FDR method.

The approach described above could lead to false negative signals if variants associated with a given trait are distributed across different windows. For this reason, we also tested associations by combining variants across entire genes. Overall, this gene-based approach tested up to 23,864 genes under the same variant selection models described above, resulting in a greater average number of variants per gene compared to the window-based approach (range 5–2,024 under the CODING scenario).

### Conditional analysis

We tested whether our RVT signals were independent from sentinel variants identified in the metabolite genome-wide association study (mGWAS) meta-analysis of INTERVAL and EPIC-Norfolk (P.S. and I.S.D., unpublished data). The sentinel variants were selected 500 kb upstream or downstream of the window of interest. To test for conditional independence, we included genotypes of the sentinel variants in the RVT as covariates. We calculated the difference of −log10(p values) before and after conditional analysis and we called it “delta”. We arbitrarily set a delta threshold of 1 and called all the RVT signals having a delta value below the threshold independent from sentinel variants.

### Forward selection procedure

We developed a forward selection procedure to identify a minimum set of variants that could explain the association in each test unit. This procedure is as follows. In the first step each variant is dropped one at a time and the test statistics recalculated. The new test statistics could either result in an increased or roughly unchanged p, which means that either the variant contributed considerably to the association p or not much. We define this p difference as “delta,” and to verify the cumulative effect of the variants with high impact, we rank all the variants by the magnitude of delta. Finally, we apply a forward selection procedure, calculating the test statistics by adding each of the ranked variants until we reach the lowest test p. The set of variants that are necessary to achieve the lowest p are called “driver” variants because they are driving the association identified with the full test unit. We found that associations detected by the burden test family were driven by many contributing variants while associations detected by SKAT had only few driver variants. In those cases where deltas are quite similar, the final set of variants might be interchangeable.

### Mediation and Mendelian randomization analysis

We used a traditional approach to mediation analysis consisting of comparing two regression models, one with and one without conditioning on the mediator. We used protein levels and metabolite levels, each as mediator, in two analyses to assess the direction of the associations between the genetic signals, protein levels, and metabolite levels.

If the dependent variable is regressed on the mediator and the genetic variants, and the size of the effect of the genetic signal remains similar, then this indicates that the mediator is unlikely to be on the causal path from the genetic variants to the dependent variable and is therefore not a mediator. However, when the effect of the genetic signal is much reduced compared to a regression model with the genetic variants as sole covariates, then this demonstrates a mediating effect on the path from the genetic variants to the dependent variable.^15^

After the mediation analysis, which established that metabolite level acts as mediator for protein level, we also carried out a two-stage least squares Mendelian randomization (MR) analysis, which uses genetic variants as the instrumental variables, metabolite level as the exposure, and protein level as the outcome variable.^16^ However, one has to keep in mind that an MR makes (among others) the assumptions that the instrument is independent of confounders and that the outcome is independent of the instrument conditioned on exposure and confounders. The latter assumption, for example, might not be fulfilled when protein level is taken as the exposure and metabolite level as the outcome variable because metabolite level is not conditionally independent of the genetic variants given protein level as seen in the above mediation analysis.

### External data sources

We searched for the rare variants identified through our analysis in the UK Biobank (UKB) summary statistics by using two sources available: (1) Global Biobank Engine (GBE) with meta-analysis of array data, including White British, European, African, South Asian, East Asian, admixed, and related,^17^ and (2) pheWEB - UKB pheWAS imputed with TOPMed.^18^

### Annotation of missense variants

We explored the deleteriousness of missense variants with a number of functional prediction scores derived from the variant effect predictor (VEP v.85): sorting intolerant from tolerant (SIFT),^19^ polymorphism phenotyping (PolyPhen),^20^ combined annotation-dependent depletion (CADD),^21^ and rare exome variant ensemble learner (REVEL).^21^^,^^22^

## Results

### Dataset and study design

**Figure 1:**
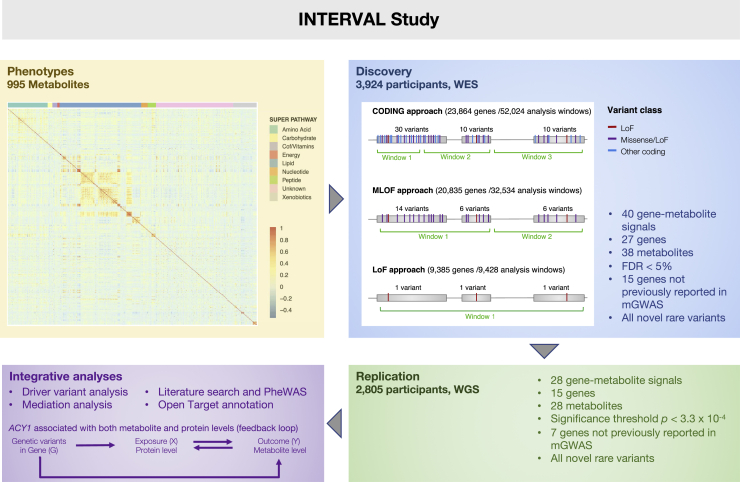
Study design, including data, methods, and results summary Data —INTERVAL study description and correlation of metabolite levels ordered by super-pathways; rare variant test strategies —analysis windows (in green) were defined to be exons containing at most 20 rare variants (MAF ≤ 0.1%), three variant selection strategies were applied (CODING, MLOF, and LOF; variant classes are color coded), and for each strategy, four rare variant aggregation tests were used to explore different allelic architectures; results —results of WES RVT analysis at 5% FDR threshold and replication of discovery signals using WGS RVT analysis. Cof/Vitamins represent the super pathway cofactors and vitamins.

We analyzed 995 metabolites measured with a non-targeted Metabolon HD4 metabolomics platform in plasma samples from 3,924 apparently healthy European-ancestry participants recruited in the INTERVAL study^23^ (see subjects and methods and Table S1). 672 metabolites (68%) were chemically identified and assigned to eight biochemical super-pathways (i.e., amino acids, carbohydrates, cofactors and vitamins, energy, lipids, nucleotides, peptides, and xenobiotics). These broad categories can be further subdivided into 79 biochemical pathways (Table S3). The remaining 323 (32%) metabolites were of unknown chemical structure. To identify rare (MAF < 0.1%) coding variants associated with metabolite levels, we accessed whole-exome sequencing (WES) of 3,924 participants (mean sequencing depth of 50×; subjects and methods), resulting in 1.72 million variants after strict QC.^24^^,^^25^ To test associations with metabolites, we applied three variant selection strategies and two classes of statistical models in order to capture a broad spectrum of possible allelic architectures. As detailed in the methods, our primary analysis was based on the definition of windows of <20 variants each, but results were compared to a whole-gene-based analysis. For all scenarios, we applied three variant selection strategies: (1) variants predicted by LOFTEE to be loss-of-function with high confidence (LOF; i.e., essential splice site changes, stop codon gain, or frameshifts);^26^ (2) missense + loss-of-function (MLOF); and (3) all coding region variants plus untranslated regions (UTRs) and essential splice sites (CODING). To achieve a comparable number of variants in each test region, we split genes into test windows of up to 20 variants on average while preserving intron-exon boundaries (GENCODE v24, Figure 1, Figure S1, Table S4, subjects and methods). For each test, we applied (1) three different implementations of burden tests (burden [BU],^27^^,^^28^ Madsen and Browning [MB],^27^^,^^28^ and variable threshold [VT]^29^), which capture associations driven by variants with similar direction and magnitude of effect (see subjects and methods) and (2) a regression-based test, i.e., the sequence kernel association test [SKAT]^30^ to capture regions that include variants with opposite direction of effects, testing for both protective and risk alleles. We applied a p values cutoff (p = 2.28 × 10^−8^) to declare genome-wide significance, corresponding to a 5% global FDR (gFDR) value correcting for all models and phenotypes tested. We tested associations surpassing this significance threshold in an independent replication sample of 2,805 whole-genome sequences from non-overlapping INTERVAL participants by applying identical test window boundaries (mean sequencing depth of 15×, subjects and methods).^27^^,^^28^

### Rare coding variants in 27 genes are associated with metabolite levels

**Figure 2:**
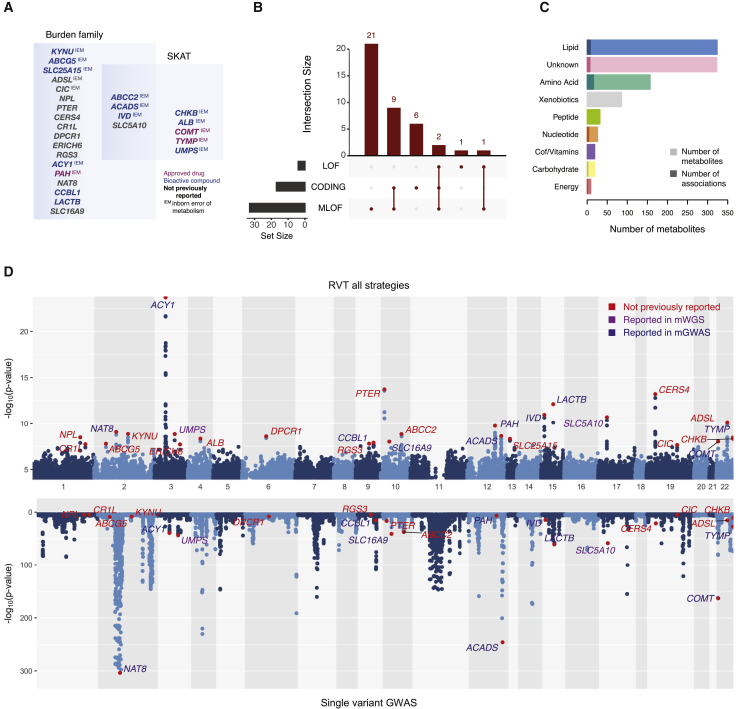
WES association results (A) List of genes discovered by type of test (burden family and/or SKAT). (B) UpSet plot of associations by approach. On the left, bar plot of total number of associations by approach and on top, bar plot of number of shared associations in multiple approaches. The number of associations in each set appears above the column, while approaches shared are indicated in the graphic below the column. (C) Bar plot of all metabolites used in the analysis split by pathway and number of associated metabolites shown in darker color. (D) Mirrored Manhattan plot showing −log10 Ps for WES single-variant tests (bottom) and WES rare-variant tests (top). Strongest gene-metabolite associations are highlighted in red. All genetic associations derived from any approach or aggregation test are reported in the RVT Manhattan plot. All 27 genes found to be associated with metabolites in RVT are labeled in the plot. Gene label color code highlights genes as not previously reported (red), reported in mWGS (purple), or reported in mGWAS (blue).

Overall, using the window-based approach, our RVTs identified 40 signals in 27 genes associated with 38 metabolites, of which LOF, MLOF, and CODING tests identified 4, 33, and 17 gene-metabolite associations, respectively (gFDR ≤ 5%; Table 1, Table S5, Figure 2). We compared our results to published metabolite GWASs based on common or low-frequency variants captured by SNP array technology (mGWAS)^2^^,^^31^^,^^32^ or rare variants reported in genome sequencing studies.^4^^,^^5^^,^^33^ Of the 27 genes with an association in this study, two (*UMPS* and *SLC5A10*) had reported association with rare variants in a WGS-based study,^5^ ten had associations involving common variants in SNP-based studies, and 15 identified new associations (described later). Overall, 4% of all metabolites were associated with at least one gene (mean 1.5 metabolites per gene, 1–7 range). Associations implicated rare genetic variants (median variant frequency = 0.013%, 148 singletons and 1 ≥ allele count [AC] ≥ 7) compared to available signals from most recent mGWAS based on SNP array imputation. The rare variant associations included between five and 19 variants/window and had minor allele count (MAC) between one and seven. Variants had large effect sizes (e.g., 21% with beta > 2 SD standardized phenotype, range −3.67–3.68 SD). The whole-gene-based testing strategy discovered 37 additional associations, 2, 30, and 15 respectively for LOF, MLOF, and CODING, and 34 associations (21 genes) matched discoveries under the window-based model (Table S5).

**Table 1:** Associations of metabolites with 27 genes that are enriched for inborn errors of metabolism and drug targets

**Gene symbol**	**Strongest metabolite**	**Super pathway**	**N driver variants**	**Variant selection strategy**	**RVT-WES p**	**RVT-WGS p**	**Replicated signal**	**mGWAS**	**OMIM disorder**	**Drug**	**Test**
*ABCC2*	X - 21467	–	2	MLOF	1.5 × 10^−9^	8.6 × 10^−6^	yes	–	Dubin–Johnson syndrome (DJS)	bioactive compound	SKAT
*ABCG5*	campesterol	lipid	9	MLOF	1.6 × 10^−8^	3.7 × 10^−4^	no	–	sitosterolemia	bioactive compound	burden
*ACADS*	butyrylcarnitine	lipid	10	CODING	2.3 × 10^−9^	1.2 × 10^−7^	yes	yes	ACYL-CoA dehydrogenase, short-chain, deficiency of (ACADSD)	bioactive compound	burden
*ACY1*	N-acetylmethionine	amino acid	14	MLOF	2.1 × 10^−24^	5.3 × 10^−17^	yes	yes	aminoacylase 1 deficiency (ACY1D)	bioactive compound	variable threshold
*ADSL*	N6-succinyladenosine	nucleotide	9	MLOF	8.3 × 10^−11^	2.8 × 10^−13^	yes	–	adenylosuccinase deficiency (ADSLD)	N/A	burden
*ALB*	X - 22771	–	1	MLOF	4.5 × 10^−9^	1.3 × 10^−4^	yes	–	analbuminaemia (ANALBA), familial dysalbuminemic hyperthyroxinemia (FDAH)	bioactive compound	SKAT
*CCBL1*	indolelactate	amino acid	10	MLOF	1.2 × 10^−8^	1.7 × 10^−5^	yes	yes	–	bioactive compound	burden
*CERS4*	sphingomyelin (d18:1/20:1, d18:2/20:0)^∗^	lipid	12	MLOF	6.2 × 10^−14^	2.9 × 10^−4^	yes	–	–	N/A	burden
*CHKB*	5-methyluridine (ribothymidine)	nucleotide	1	CODING	4.8 × 10^−9^	7.2 × 10^−1^	no	–	congenital Muscular dystrophy, megaconial type	bioactive compound	SKAT
*CIC*	1-(1-enyl-stearoyl)-2-linoleoyl-GPE (P-18:0/18:2)^∗^	lipid	14	CODING	2.2 × 10^−8^	9.5 × 10^−1^	no	–	mental retardation, autosomal dominant 45 (MRD45)	N/A	burden
*COMT*	X - 11593	–	1	MLOF	9.2 × 10^−9^	1.2 × 10^−1^	no	yes	panic disorder 1 (PAND1), schizophrenia (SCZD)	approved drug	SKAT
*CR1L*	X - 21444	–	10	CODING	1.8 × 10^−8^	9.5 × 10^−1^	no	–	–	N/A	Madsen and Browning
*DPCR1*	2-aminobutyrate	amino acid	14	CODING	2.5 × 10^−9^	3.5 × 10^−1^	no	–	–	N/A	burden
*ERICH6*	glycerophosphorylcholine (GPC)	lipid	4	LOF	1.9 × 10^−8^	2.3 × 10^−1^	no	–	–	N/A	variable threshold
*IVD*	isovalerylcarnitine	amino acid	7	MLOF	1.1 × 10^−11^	3.5 × 10^−3^	no	yes	isovaleric acidemia (IVA)	bioactive compound	burden
*KYNU*	xanthurenate	amino acid	8	MLOF	1.4 × 10^−9^	4.3 × 10^−9^	yes	–	hydroxykynureninuria; Vertebral, cardiac, renal and limb defects syndrome 2 (VCRL2)	bioactive compound	burden
*LACTB*	succinylcarnitine	energy	9	MLOF	7.6 × 10^−13^	5.5 × 10^−6^	yes	yes	–	bioactive compound	variable threshold
*NAT8*	N-acetylarginine	amino acid	8	MLOF	8.4 × 10^−10^	1.3 × 10^−5^	yes	yes	–	N/A	burden
*NPL*	N-acetylneuraminate	carbohydrate	10	MLOF	3.3 × 10^−9^	1.5 × 10^−9^	yes	–	–	N/A	burden
*PAH*	phenylalanine	amino acid	9	MLOF	1.7 × 10^−10^	5.2 × 10^−8^	yes	yes	phenylketonuria (PKU) and hyperphenylalaninemia, non-PKU	approved drug	Madsen and Browning
*PTER*	N-acetyl-beta-alanine	nucleotide	11	MLOF	1.9 × 10^−14^	3.3 × 10^−8^	yes	–	–	N/A	Madsen and Browning
*RGS3*	stearoyl sphingomyelin (d18:1/18:0)	lipid	8	MLOF	1.6 × 10^−8^	3.1 × 10^−1^	no	–	–	N/A	variable threshold
*SLC16A9*	carnitine	lipid	9	MLOF	9.5 × 10^−9^	4.3 × 10^−1^	no	yes	–	N/A	variable threshold
*SLC25A15*	X - 15728	–	12	CODING	4.8 × 10^−9^	5.3 × 10^−1^	no	–	hyperornithemia-hyperammonemia-homocitrullonuria syndrome	bioactive compound	burden
*SLC5A10*	1,5-anhydroglucitol (1,5-AG)	carbohydrate	6	LOF	2.0 × 10^−11^	5.4 × 10^−5^	yes	rare	–	N/A	burden
*TYMP*	5-methyluridine (ribothymidine)	nucleotide	1	CODING	3.5 × 10-9	6.9 × 10^−3^	no	yes	mitochondrial DNA depletion syndrome-1 (MTDPS1)	approved drug	SKAT
*UMPS*	Orotate	nucleotide	1	MLOF	1.4 × 10^−9^	2.4 × 10^−5^	yes	rare	orotic aciduria	bioactive compound	SKAT

We attempted to replicate the associations by using WGS data from 2,805 participants from the INTERVAL study not overlapping with the WES dataset. For 15 genes associated with 28 metabolites, we replicated associations between the reported window and the reported metabolite at a stringent Bonferroni correction (p ≤ 0.05/149 = 3.3 × 10^−4^, Table S6), implicating ACY1, PTER, ADSL, NPL, KYNU, PAH, ACADS, LACTB, ABCC2, NAT8, CCBL1, UMPS, SLC5A10, ALB, and CERS4. The remaining associations did not reach this stringent level of significance because of absence of the corresponding rare variants from the WGS data.

We also attempted to replicate associations by using an independent metabolomic platform (Nightingale Health) based on NMR,^9^ which includes 226 metabolites of different classes (ketone bodies, glycolysis related metabolites, amino acids, fluid balance, inflammation, fatty acids and saturation, cholesterol, glycerides and phospholipids, apolipoproteins, lipoprotein subclasses, and lipoprotein particle sizes). We tested the 27 genes by using models identical to the Metabolon-based discovery. Two of the relevant metabolites were shared between the metabolomics platforms, and associations were confirmed at two of the gene-metabolite associations, *PAH*-phenylalanine and *RGS3*-sphingomyelin (p < 4.3 × 10^−5^). Additionally, we scanned the phenotypes by searching for associations of the significant 27 genes with any of the 226 NMR metabolites, but we did not find any additional associations (Bonferroni p < 8.19 × 10^−6^) (Table S7).

Finally, we carried out conditional analyses to test whether the RVT associations may be explained by the presence of nearby common variants. We modeled the RVT associations while conditioning for nearby (<500 kb) common sentinel variants identified through a meta-analysis of INTERVAL and EPIC-Norfolk studies (P.S. and I.S.D., unpublished data) and found that most (93%) of the RV associations were independent from the proximal sentinel common variants, while *ACADS*-ethylmalonate and *NAT8*-N-acetyltyrosine narrowly missed the cutoff for independence (Table S8 and subjects and methods). We also used the WES data to search for novel metabolite associations with common coding genetic variants (MAF > 0.1%, n = 44,135) and we detected 1,836 signals in 580 genes at exome-wide significance level (p < 2.63 × 10^−9^) that had all been previously reported in mGWASs.

### Biological, biochemical, and functional interpretation of associations

We next investigated the genetic architecture of each locus and their biochemical characteristics in detail. We assessed the proteins and diseases that are reportedly linked to each gene-metabolite pair, employing a range of bioinformatic tools and data repositories.^34^^,^^35^ Overall, the majority of associations had a clear biochemical rationale and could plausibly be explained by protein-coding variants altering the efficiency of the enzyme reactions (Table S9). To inform the genetic structure of each locus, we used two alternative approaches based on either leave-one-out/forward selection or Lasso (subjects and methods) to identify minimal sets of variants with the greatest probability of accounting for the RVT association signal (“driver” variants, which are not necessarily all causal variants).

**Figure 3:**
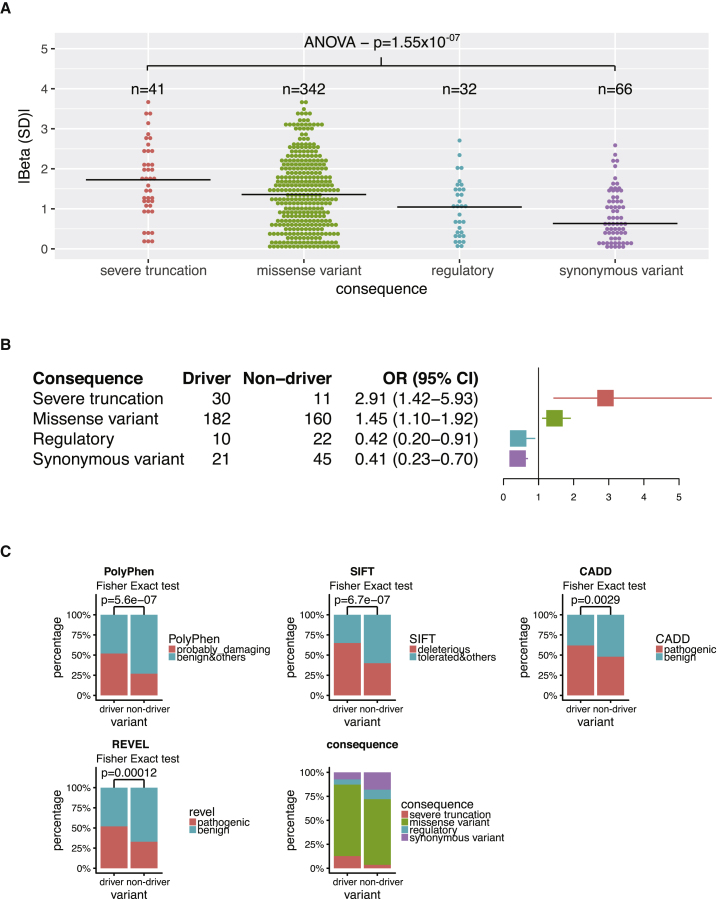
Driver variants analyses (A) Absolute effect size (beta in SD) of driver variants split by their predicted consequence on protein. (B) Enrichment of driver variants split by their predicted consequence on protein. (C) Enrichment of driver variants by different functional prediction methods: Polyphen, SIFT, CADD, REVEL, and predicted consequences.

Using the forward selection approach, we found that the majority of SKAT signals could be accounted for by only one or two driver variants, whereas the number of driver variants in burden tests was greater (4–14). Overall, the proportion of driver variants identified by Lasso and forward selection was 55%, and 66% if SKAT tests were excluded from the comparison (Figure S2). We found that driver variants predicted to cause severe protein truncation tended to have higher effect sizes when compared to other functional classes (Figure 3). Driver variants were enriched for protein-truncating variants (i.e., splice donor, splice acceptor, frameshift, and stop gained variants) with 3-fold increased odds. However, missense was the most represented category among all rare variants and we show that missense driver variants are significantly enriched for being likely deleterious (Figure 3 and subjects and methods). For 12 genes, we were able to confirm for each variant the biological consistency of the direction of effect between gene and metabolite. This means that in some cases the substrate metabolites accumulate if missense or LoF variants are decreasing the efficiency of the enzyme or transporter, while in other cases disrupted gene function results in reduced levels of the metabolite product (Table S5). In the following sections we focus on results generated from the window-based approach.

### Neurological function

We observed associations of 18 rare variants in *ACY1* (14 drivers) with seven metabolites, including N-acetylmethionine (VT test, p_WES_ = 2.1 × 10^−24^, p_WGS_ = 5.3 × 10^−17^), five N-terminal acetylated amino acids (N-formylmethionine, N-acetylserine, N-acetylglutamate, N-acetylthreonine, N-acetylvaline, and N-acetylalanine), and N-formylmethionine (Table S10). *ACY1* encodes aminoacylase-1, a homodimeric zinc-binding metalloenzyme involved in the hydrolysis of N-acetylated proteins. The two most significant encoded non-synonymous changes are predicted to disrupt protein function: frameshift p.Ser192fs (rs770702363, MAF = 0.051%, beta(SE) = 2.449(0.4997), p = 9.45 × 10^−7^) and missense p.Asp174Gly (rs200314495, MAF = 0.013%, beta(SE) = 3.679(0.999), p = 2.30 × 10^−4^, SIFT = deleterious, PolyPhen = probably damaging). rs770702363 is predicted to be pathogenic and located within the M20 peptidase domain, in the proximity of metal ion binding (protein position: 175). The remaining driver variants were all missense changes, with the exception of another frameshift variant 3:51987362:C:CT (Table S5).

A possible interpretation of the association is that the combined effect of missense variants may reduce the overall enzymatic activity of aminoacylase 1, leading to an increase of their substrate (in this case N-acetylated amino acids). *ACY1* has been described in OMIM as a gene causing aminoacylase 1 deficiency (ACY1D), a rare inborn error of metabolism characterized by increased urinary excretion of specific N-acetyl amino acids, and most affected individuals show neurologic abnormalities such as intellectual disability, seizures, hypotonia, and motor delay.^36^ We therefore searched two UKB PheWAS databases, GBE^17^ and PheWeb,^18^ for associations with complex neurological phenotypes (subjects and methods). Driver variant rs6804746 (MAF = 0.02%) was associated with ICD10 code G31 (“other degenerative diseases of the nervous system not elsewhere classified”, GBE; p = 4.6 × 10^−8^) and with chronic fatigue syndrome (PheWeb; p = 3.5 × 10^−4^). Two other driver variants associated with mental disorders in PheWeb, namely rs1164299165 with pervasive developmental disorders (MAF = 0.005%; p = 9.7 × 10^−4^) and rs887540 with major depressive disorder (MAF = 0.03%; p = 3 × 10^−4^).

### Complex carbohydrates and cellular infectivity

Rare variants in *NPL* were associated with increased N-acetylneuraminate (burden test, p_WES_ = 3.2 × 10^−9^, p_WGS_ = 1.5 × 10^−9^). *NPL* encodes a member of the N-acetylneuraminate lyase subfamily, which regulates cellular concentrations of N-acetylneuraminate by mediating the reversible aldol condensation between N-acetyl-d-mannosamine (ManNAc) and pyruvate to N-acetylneuraminate.

The signal included 15 variants (ten drivers), including a missense (rs141892236, MAF = 0.090%, beta(SE) = 1.552(0.378), p = 4.13 × 10^−5^) and a splice donor variant (rs757256606, MAF = 0.026%, beta(SE) = 2.005(0.707), p = 4.62 × 10^−3^) and several other missense changes close to the active protein sites at positions 143 and 173 (rs146355388, p.Pro146Thr, MAF = 0.039%, beta(SE) = 1.550(0.578), p = 7.32 × 10^−3^; rs148306247, p.Glu156Ala, MAF = 0.090%, beta(SE) = 0.654(0.378), p = 8.43 × 10^−2^; rs138338286, p.Glu157Val, MAF = 0.026%, beta(SE) = 0.685(0.707), p = 3.33 × 10^−1^). We can hypothesize that reduced enzymatic activity of NPL driven by rare variation may lead to the accumulation of N-acetylneuraminate, which is then recycled and returned to the cell surface, potentially increasing susceptibility to bacterial and viral pathogenicity. Interestingly, rs148306247, rs146355388, and rs138338286 were all associated with different infection-related traits in both GBE and PheWeb, reflecting the importance of the N-acetylneuraminate in host-parasite interactions. Finally, rs141892236 was associated with septicemia in GBE (p = 2.3 × 10^−11^), while a weaker signal for Streptococcus infection was found in PheWeb (p = 3 × 10^−3^), reinforcing the role of this gene in viral and bacterial infection.

### Lipids homeostasis and phytosterols

We observed an association between variants in *ABCG5* and an increase in campesterol levels (burden test, p_WES_ = 1.6 × 10^−8^, p_WGS_ = 3.7 × 10^−4^). *ABCG5* encodes ATP-binding cassette subfamily G member 5 (ABCG5), an ABC transporter involved in the lipid homeostasis pathway transporting sterols from the cytosol to the extracellular domain, limiting intestinal absorption and promoting biliary excretion of sterols. Campesterol is a phytosterol (PS), or a steroid derived from plants. As a food additive, phytosterols have cholesterol-lowering properties (by reducing cholesterol absorption in intestines) and may act in cancer prevention. The signal included 14 variants (nine drivers), of which the two most significant associations encode for missense changes (rs755523464, p.Tyr487Cys, MAF = 0.025%, beta(SE) = 3.491(1.002), p = 4.94 × 10^−4^ and 2:43822798:A:G, p.Trp488Arg, MAF = 0.025%, beta(SE) = 3.185(1.002), p = 1.48 × 10^−3^) (Table S5).

We hypothesize that rare variants in *ABCG5* could reduce the efficiency in transporting sterols, therefore increasing plasma dietary campesterol. The missense variant rs150401285 (MAF = 0.1%) was associated with cholesterol (p = 4.9 × 10^−14^), LDL cholesterol (p = 1.1 × 10^−13^), and apolipoprotein B (p = 3.9 × 10^−9^) in GBE and with cholelithiasis and cholecystitis in PheWeb (p = 4.6 × 10^−4^), indicating that loss of *ABCG5* activity due to rs150401285 results in a protective effect on gallstones. Interestingly, a recent GWAS of gallstone disease^37^ showed that the missense p.Asp19His variant in *ABCG5*/*ABCG8* (gain-of-activity) increases the risk for gallstone disease through increases in biliary cholesterol secretion and decreases in dietary cholesterol intake in the gut. It remains to be determined whether this association is mediated by decreased cholesterol levels.

### Kynurenine pathway

Variants in *KYNU* were associated with increased levels of xanthurenate, a metabolite from the tryptophan catabolism (burden test, p_WES_ = 1.4 × 10^−9^, p_WGS_ = 4.3 × 10^−9^). *KYNU* encodes for Kynureninase (EC 3.7.1.3), a 3-hydroxykynureninase-type enzyme involved in the kynurenine pathway for the biosynthesis of NAD cofactors from tryptophan. It catalyzes the conversion of L-3-hydroxykynurenine and L-kynurenine to 3-hydroxyanthranilic acid and anthranilic acid, respectively. The signal included 12 variants (eight drivers), of which two missense variants with the strongest association (rs137982021, MAF = 0.065%, beta(SE) = 2.172(0.447), p = 1.20 × 10^−6^ and 2:142985130:A:G, MAF = 0.013%, beta(SE) = 3.065(0.999), p = 2.17 × 10^−3^) encode changes predicted to impact the catalytic efficiency of Kynureninase, leading to an increase in the levels of L-3-hydroxykynurenine (substrate) that is converted to xanthurenate by kynurenine aminotransferase (KATs). The amino acid substitutions that we identified span protein positions 212 to 432, corresponding to the aminotransferase domain. Further, they are all located downstream of the reported homozygous mutation (encoding p.Thr198Ala) that causes hydroxykynureninuria, an inborn error of metabolism characterized by accumulation of kynurenine, 3-hydroxykynurenine, and xanthurenic acid excreted in the urine.^38^ The absence of kynureninase results in a block in the pathway from tryptophan to nicotinic acid, and can result in niacin (vitamin B3) deficiency. The clinical phenotype has a wide range from asymptomatic to severe, characterized by intellectual disability, cerebellar ataxia, pellagra, progressive encephalopathy with muscular hypotonia, global developmental delay, stereotyped gestures, and/or congenital deafness.

*CCBL1* is another signal that is part of the tryptophan pathway and was associated with increased levels of indolelactate (burden test, p_WES_ = 1.2 × 10^−8^, p_WGS_ = 1.7 × 10^−5^). *CCBL1* encodes for kynurenine aminotransferase (KYAT1; EC 2.6.1.7) and it is part of the tryptophan catabolism pathway that converts L-kynurenine and L-3-hydroxykynurenine into kynurenate and xanthurenate, respectively. Indolelactate is also part of the tryptophan catabolism pathway metabolized via a series of indoles. This process is mainly enabled by gut microbiota and in particular *Clostridia*. Though the direct biochemical mechanism of this association is still unclear, it is interesting to note that serum levels of indolelactate were found to be significantly lower in adults with multiple sclerosis, as well as the bacteria producing it.^39^

KYNU and CCBL1 are enzymes of the kynurenine pathway that have multiple biological implications, such as an active role in the immune response; some kynurenins are neuroactive and the kynurenine pathway is involved in many diseases such as Alzheimer's disease, amyotrophic lateral sclerosis, Huntington's disease, AIDS dementia complex, malaria, cancer, depression, and schizophrenia, where imbalances in tryptophan and kynurenines have been found.^40^ Specifically, the missense variant rs137982021 (MAF = 0.03%) was associated with cholecystitis without cholelithiasis (p = 2.6 × 10^−4^), renal failure NOS (p = 1.7 × 10^−3^), and other specified cardiac dysrhythmias (p = 1.8 × 10^−3^) in PheWeb, confirming its implication in a broad range of disease categories.

### Signals overlapping with drug targets

In total, 66% of the rare variant associations identified in our study were found within genes of pharmacological interest. Three genes (*COMT*, *TYMP*, and *PAH*) discovered in our study overlap drug targets for four approved drugs (entacapone, tolcapone, tipiracil, and sapropterin). In addition, 12 of the genes (*CHKB*, *UMPS*, *ALB*, *ABCC2*, *IVD*, *KYNU*, *LACTB*, *ABCG5*, *CCBL1*, *ACADS*, *SLC25A15*, and *ACY1*) are targets for bioactive drug-like compounds that were experimentally validated in ChEMBL. Entacapone and tolcapone are both inhibitors of catechol-O-methyltransferase (COMT), used in the treatment of Parkinson's disease as an adjunct to levodopa/carbidopa therapy. COMT eliminates biologically active catechols and other hydroxylated metabolites. In the presence of a decarboxylase inhibitor, COMT becomes the major metabolizing enzyme for levodopa, catalyzing the metabolism to 3-methoxy-4-hydroxy-L-phenylalanine (3-OMD) in the brain and periphery. The mechanism of action of entacapone is believed to be through its ability to inhibit COMT and alter the plasma pharmacokinetics of levodopa. The gene target and two inhibiting drugs are also associated with several other brain-related diseases and addictions, such as schizophrenia, cocaine dependence, gambling behavior (phase II), and epilepsy. Tipiracil is a small molecule first approved in 2015 and indicated for the treatment of adults with metastatic colorectal cancer. Tipiracil selectively inhibits thymidine phosphorylase (TYMP), a cytosolic enzyme essential for the nucleotide salvage pathway. Sapropterin was approved in 2007 and is today a well-established drug for the treatment of phenylketonuria. It is a small molecule targeting phenylalanine hydroxylase (PAH) to activate the hydroxylation of L-phenylalanine to L-tyrosine. Sapropterin has recently been associated with other indications in phase III and IV, including hyperphenylalaninemia and peripheral arterial disease.

### Mediation and Mendelian randomization analysis via protein level

For a subset of the participants included in this study (n = 3,301), we also interrogated the plasma proteome, using an expanded version of an aptamer-based multiplex protein assay (SOMAscan, Somalogic)^41^ to quantify 3,622 plasma proteins.^10^ We conducted RVT for all our metabolite-associated genes, searching for *cis* and *trans* associations with available protein levels by using the same analysis strategy as described previously (the same variants, the same grouping within windows, and the same selection strategy). We identified a significant (p = 5.9 × 10^−9^; beta = −1.09; MLOF approach) *cis* protein level association within *ACY1*—our strongest signal in the Metabolon analysis. The same rare genetic variants in *ACY1* were associated with metabolite and protein levels, implying highly concordant allelic architectures.

**Figure 4:**
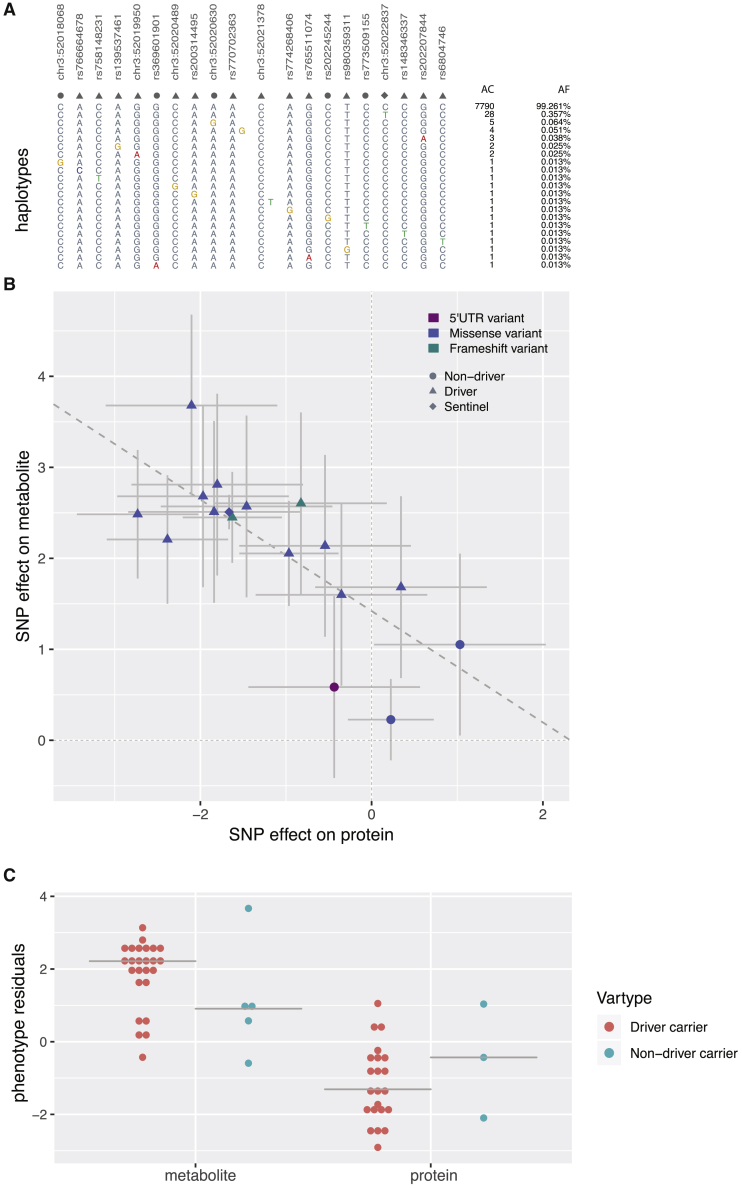
*ACY1*/N-acetylmethionine in depth analysis (A) Proportions of haplotypes composed of non-driver, driver, and sentinel variants that are represented by their point shape in (B). (B) Scatter plot of effect sizes and relative standard errors of shared protein versus metabolite associations; color coding indicates predicted consequence on protein and point shape indicates type of variants. (C) Dot plots of phenotype residuals in carriers of driver and non-driver variants for both metabolite and protein levels.

To investigate the predicted functional impact of rare *ACY1* variants, we performed a structural analysis of this protein by using UCSF Chimera^42^ and evaluated the impact of substituting the different rotamers on the protein structure. The p.Gln26Pro substitution had a potential to create steric clashes in the proximity of the active site of the enzyme. To investigate the relationship between the protein and metabolite levels associated with *ACY1*, we performed a mediation analysis, testing two alternative models where N-acetylmethionine and ACY1 protein levels were fit as covariates in a model testing associations of rare variants with protein and N-acetylmethionine levels, respectively (Figure 4). The results were consistent with a scenario where N-acetylmethionine level may mediate associations of genetic variants to ACY1 protein levels.

Once we established that the metabolite level lies on the causal path from the genetic variants to protein level, we aimed to isolate the direct effect of metabolite level on protein level from potential confounders via Mendelian randomization (MR). Our MR analysis uses the genetic variants as the instrumental variable, metabolite level as the exposure, and protein level as the outcome variable. A two-stage analysis showed that the MR estimate for the effect of metabolite level on protein level (beta = −0.615, p < 2 × 10^−16^) is very similar to the estimate from a linear model (beta = −0.592, p < 2 × 10^−16^) without an instrument. This indicates that the relatively strong negative influence of metabolite levels on protein levels is genuine and unlikely to be the effect of confounding.

## Discussion

In this study, we describe an association study of rare genetic variants with blood plasma concentrations of 995 metabolites in almost 4,000 apparently healthy blood donors. We identified 40 gene-metabolite associations in 27 genes and 38 metabolites by using a windows-based approach. Of these, only one association driven by a rare variant has been already described (rs200305064 with orotate);^5^ for 11 additional genes, there was previous evidence for association at the same locus but driven by independent common genetic variants. Signals from 15 genes (seven new) were replicated with WGS data from the same study, while for the others, replication p values did not reach the predefined significance cutoff, most likely through a lack of the corresponding driver variants.

Our rare variant test strategy was designed to explore different allelic architectures through multiple approaches. Compared with the approach used by Long et al.,*^5^* we sought to increase statistical power by aggregating variants of different predicted functional effects within genomic windows defined by intron-exon boundaries. This allows us to detect associations where gene-wide associations could not be detected, for instance, in the case of the *ABCG5* gene associated with campesterol or *RGS3* with stearoyl sphingomyelin (d18:1/18:0). In the latter case, the third test window contained multiple functional domains more likely to harbor rare variants disrupting the sphingomyelin pathway. However, this strategy reduces power for cases where the contributing variants are spread across the gene, and indeed we found 37 associations that surpassed the significance threshold at gene level but not in the window scenario. Overall, the MLOF approach yielded a greater number of new discoveries and only one association was specific to the LOF approach. This most likely reflects an optimal number of functional variants included in the testing windows, for the current sample size, and underlying allelic architecture. As expected, multiple RVT signals were shared among different test types and especially between MLOF and CODING (eight genes in total). Most of the associations were identified by the aggregated contributions of many singletons and fewer variants with higher allele count. Interestingly, 21% of these variants presented with an effect size greater or equal to 2, demonstrating the power of WES to identify rare variants with large effect sizes within genes of pharmacological interest. Conditional analyses using nearby (<500 kb) common sentinel variants identified through mGWAS confirmed that RVT associations are mostly independent from proximal common sentinel variants.

Our algorithms to identify putative driver variants, i.e., variants that are more likely to contribute to the association signal, confirms different architectures underlying SKAT and burden signals, and SKAT tests are typically explained by small numbers of variants of greater allelic frequencies. Driver variants were enriched for variants of predicted functional impact, for instance causing a severe truncation, and missense variants predicted as deleterious by multiple approaches (Polyphen, SIFT, CADD, and REVEL), confirming the validity of this approach.

The new associations were enriched near genes causative for inborn error of metabolism (IEM) and genes associated in mGWASs of common variants. The allele frequencies and effects sizes of the new associations were intermediate between the two, confirming a continuum of genetic contributions to metabolic function mediated by the same genes. While as much as 55% of RVT associations were in IEM genes, only a handful were known pathological variants for recessive diseases (ClinVar or OMIM) for which no healthy homozygous carriers were present in our study participants.

For almost all of the gene-metabolite associations, we were able to identify the underlying biochemical function, where these functions implicated genes with important biomedical functions. For instance, *NPL*, which is associated with N-acetylneuraminate (sialic acid, NANA, Neu5Ac) belongs to an ancient pathway conserved in bacteria. N-acetylneuraminate is an essential component of complex carbohydrates, which play pivotal roles in a variety of cellular recognition and communication processes including host-parasite interactions. Another example is the association between *ABCG5/G8* and phytosterols (PSs). Exogenous sterols (including PS) have been shown to have cholesterol-lowering properties. Reduction of up to 15% achieved in human subjects^43^ may be mediated by competitive intestinal solubilization into mixed cholesterol/PS micelles, or increases in intestinal and hepatic-biliary secretion mediated by *ABCG5/G8* upregulation by PS. Consequently, several studies have reported correlations between phytosterol levels and cardiovascular health^44^ mediated by common variants in *ABCG8* and *ABO*.

Our hypothesis-free approach revealed new hypotheses on the mechanisms through which associations with metabolites may act. We compared our associations with a dataset of proteins and found concomitant associations with protein levels at the *ACY1* locus. Interestingly, we found that variants associated with an accumulation of N-acetylmethionine were also associated with a decrease of ACY1 protein levels. Through a mediation analysis, we infer a directional effect whereby protein levels are mediated by the accumulation of metabolite, which would suggest the existence of negative feedback of the metabolite onto the protein. A possible hypothesis is that rare LoF and missense variants, which are predicted in this study to cause steric clashes at the active site, may reduce the efficiency of the protein in clearing the substrate. The potential medical impact of this association extends beyond the neurological function described earlier. A recent study has described a strong positive correlation between ACY1 protein levels and type 2 diabetes (T2DM).^45^ Through *in vitro* and *in vivo* experiments, the authors showed that increasing amounts of ACY1 decreased the ratio of N-acetyl/free amino acids, with a consequent effect on glucose and insulin homeostasis, possibly leading to β-cell exhaustion, reduced β-cell mass, and ultimately insulin deficiency and T2DM. In another recent study in a subset of participants from the INTERVAL study, a polygenic risk score for T2DM was associated with ACY1 protein levels, thus strengthening the link between *ACY1* and T2D risk.^46^

Overall, our findings illustrate the value of endophenotypes including metabolites and proteins to enhance our understanding of previously known genetic risk factors for disease. This is even more evident when, as in our case, sequencing data were used for identification of rare coding variants with large effect sizes associated with metabolite/protein levels. These studies generate new hypotheses to support therapeutic target identification and validation.

## Data Availability

Whole-exome sequencing data for the INTERVAL cohort is available in EGA: https://www.ebi.ac.uk/ega/datasets/EGAD00001002221. All of the summary statistics are available at Sanger ftp site: ftp://ftp.sanger.ac.uk/pub/project/humgen/summary_statistics/INTERVAL_WES_metabolon. All of the codes for this study are publicly available at GitHub: https://github.com/teamsoranzo/MetabolomicsWorkflow.

## Web resources

Clinvar, https://www.ncbi.nlm.nih.gov/clinvar/
Global Biobank Engine, https://biobankengine.stanford.edu/
HGMD, https://www.hgmd.cf.ac.uk/
Loftee, https://github.com/konradjk/loftee
Open Targets, https://platform.opentargets.org/
Orphanet, https://www.orpha.net/
UK Biobank TOPMed-imputed PheWeb, https://pheweb.org/UKB-TOPMed/
Vep, https://www.ensembl.org/info/docs/tools/vep/index.html

## Supplemental information

Document S1. Supplemental note, supplemental acknowledgments, and Figures S1 and S2

Table S1. Study participant characteristics expressed in counts and percentages

Table S2. Linear regressions of the first five principal components against age, sex, BMI, current smoking, alcohol consumption frequency, center, batch, plate, appointment month and time between appointment and processing

Table S3. Study metabolite counts per pathway categories

Table S4. Summary statistics of different strategies used in the study such as number of genes tested in each strategy

Table S5. Table of all gene-metabolite associations including variants used in the test, type of test and strategy used, whether variants were drivers or not, recalculated Ps when including only driver variants, gene reported in previous mGWAS and OMIM annotation Also includes overall association results for window-based and gene-based analysis.

Table S6. WGS replications of discovery signals

Table S7. Summary statistics of associations of previously identified genes (Metabolon, this study) with NMR measured metabolites levels

Table S8. Conditional analysis of RV discoveries with sentinel variants from imputed data

Table S9. Biological annotation of rare variant test (RVT) signals

Table S10. Details of all associations discovered based on strategies and test categories

Document S2. Article plus supplemental information
