# Supplementary material for: Whole-exome sequencing identifies rare genetic variants associated with human plasma metabolites
Source: Am J Hum Genet. 2022 May 13;109(6):1038–54. doi: 10.1016/j.ajhg.2022.04.009 (PMC9247822; doi:10.1016/j.ajhg.2022.04.009)
Supplement: Document S1. Supplemental note, supplemental acknowledgments, and Figures S1 and S2 [file mmc1.pdf]

The American Journal of Human Genetics, Volume *109*

Supplemental information

Whole-exome sequencing identifies rare genetic variants associated with human plasma metabolites

Lorenzo Bomba, Klaudia Walter, Qi Guo, Praveen Surendran, Kousik Kundu, Suraj Nongmaithem, Mohd Anisul Karim, Isobel D. Stewart, Claudia Langenberg, John Danesh, Emanuele Di Angelantonio, David J. Roberts, Willem H. Ouwehand, INTERVAL study, Ian Dunham, Adam S. Butterworth, and Nicole Soranzo

Supplemental Note

In this section we provide an in-depth description of all the gene-metabolite associations with a biological and when possible clinical interpretation, they are ordered by approach (MLOF, LOF, CODING) and metabolite super pathways (amino acids, lipids, carbohydrates, nucleotides, energy, unknown)

MLOF test

Out of the 20 genes, 6 (*ACY1, IVD, KYNU, CCBL1, PAH, NAT8*) were associated with 13 metabolites classified as amino acids.

The association between *ACY1* and N-acetylmethionine is our top hit ($P=2.1\times10^{-24}$) and was identified by the variable threshold test. In total *ACY1* was associated with seven acetyl/formyl amino acids by all family burden tests used in the analysis. A total of 18 variants were included in the test and were associated with increased metabolite level. This gene has been described in OMIM as a gene-causing aminoacylase 1 deficiency (ACY1D), a rare form of inborn error of metabolism characterized by increased urinary excretion of specific N-acetyl amino acids. Most patients show neurologic abnormalities such as intellectual disability, seizures, hypotonia, and motor delay. ACY1D is an ultra-rare autosomal recessive condition with unknown prevalence of affected children and only 15 cases reported world-wide. Diagnosis is made by gas chromatography-mass spectrometry (GC-MS) analysis of urinary organic acids revealing increased levels of N-acetylated amino acids. The prognosis is unknown and the management is symptomatic only. *ACY1* is located on chromosome 3p21 that comprises 15 exons and encodes for aminoacylase-1. Aminoacylase-1 (EC 3.5.1.14) is a homodimeric zinc-binding metalloenzyme, localized in the cytoplasm and expressed in a wide range of tissues. Aminoacylase-1 is the most abundant of the aminoacylases, a class of enzymes involved in hydrolysis of N-acetylated proteins when they are no longer needed. Many proteins in the body have an acetyl group attached to one end, and this modification, called N-acetylation, helps to protect and to stabilize the protein. Aminoacylase 1 performs the final step in the breakdown of these proteins by removing the acetyl group from certain amino acids except L-aspartate. The amino acids can then be recycled and used to build other proteins. N-terminal acetylation of proteins is a widespread and highly conserved process that is involved in the protection and stability of proteins. However, ACY1 can also catalyze the reverse reaction, the synthesis of acetylated amino acids. ACY1 may also play a role in xenobiotic bioactivation as well as the inter-organ processing of amino acid-conjugated xenobiotic [1]derivatives (S-substituted-N-acetyl-L-cysteine). ACY1 appears to physically interact with Sphingosine kinase type 1 (SphK1) and may influence its physiological functions [1]; SphK1 and its product sphingosine-1-phosphate have been shown to promote cell growth and inhibit apoptosis of tumor cells. Overexpression of aminoacylase 1 is associated with colorectal cancer progression [2]. Enzyme knockdown inhibits cell proliferation and causes cell cycle perturbation. Strong expression of the human gene and its mouse orthologous Acy1 in the brain, liver, and kidney, suggest a role of the enzyme in amino acid metabolism of these organs.

The *IVD* gene was associated with an increase in isovalerylcarnitine levels ($P=1.1\times10^{-11}$) including 14 variants. Isovaleryl-CoA dehydrogenase (EC 1.3.99.10) is a member of the acyl-CoA dehydrogenase family and is involved in the catabolism of leucine.

Mutations in *IVD* are reported in OMIM to cause Isovaleric acidemia. It can present with severe neonatal ketoacidosis leading to death, but in milder cases recurrent episodes of ketoacidosis of varying degree occur later in infancy and childhood [3 4].Isovaleric acidemia is an autosomal recessive inborn error of leucine metabolism caused by a deficiency of the mitochondrial enzyme isovaleryl-CoA dehydrogenase (IVD) that results in the accumulation of derivatives of isovalerate, isovalerylglycine, and isovalerylcarnitine. IVD is an enzyme in the L-leucine degradation pathway that specifically catalyzes the conversion of isovaleryl-CoA into 3-methylcrotonyl-CoA. Thus, gene disruption or mutation results in accretion of plasma isovalerylcarnitine (as well as isovalerylglycine and isovalerate) to toxic levels and is the key phenotypic features associated with IVD gene function [5]. The rs28940889[6] variant is one of the variants included in the test and is also reported in OMIM and ClinVar (Isovaleryl-CoA dehydrogenase deficiency;not provided). This could potentially be of interest since this mutation was found in healthy adults.

The *KYNU* gene was associated with increased levels of xanthurenate which is a metabolite from tryptophan catabolism, aka 8-Hydroxykynurenate. This association was identified through a burden test ($P=1.4\times10^{-09}$) including a total of 12 rare variants. Kynureninase (EC 3.7.1.33) is a 3-hydroxykynureninase-type enzyme involved in the kynurenine pathway for the biosynthesis of NAD cofactors from tryptophan. It catalyzes the conversion of L-3-hydroxykynurenine and L-kynurenine to 3-hydroxyanthranilic acid and anthranilic acid, respectively. The reaction is pyridoxal-5-prime-dependent and is sensitive to nutritional vitamin B6 deprivation in mammals. Studies in mouse, rat, and pig suggest that kynureninase is a 95-kD homodimer predominantly located in the cytoplasm [7]. Kynureninase is also involved in the de novo NAD(H) synthesis pathway, using niacin from dietary input [8]. Mutations in *KYNU* have been described to cause hydroxykynureninuria. [9] described a female patient, an only child, who excreted large amounts of kynurenine, 3-hydroxykynurenine, and xanthurenic acid in the urine. Absence of kynureninase resulting in a block in the pathway from tryptophan to nicotinic acid, was postulated. Under these circumstances, tryptophan is no longer a source of nicotinic acid and deficiency of the vitamin can develop. The mother excreted 3 to 4 times the normal amounts of xanthurenic acid. The father's excretion was at the upper limit of normal. The *KYNU* mutation could lead to vertebral, cardiac, renal and limb defects syndrome 2 (VCRL2VCRL2) which is an autosomal recessive congenital malformation syndrome characterized by vertebral segmentation abnormalities, congenital cardiac defects, renal and distal mild limb defects. In two unrelated patients with VCRL2, [8] identified homozygous truncating mutations in the *KYNU* gene. The mutations, which were found by whole-exome sequencing and confirmed by Sanger sequencing, segregated with the disorder in the families. In vitro functional expression studies showed that the mutations essentially abolished any KYNU enzymatic activity. Analysis of plasma from one patient showed increased levels of the upstream metabolite L-3-hydroxykynurenine and decreased levels of the downstream metabolites NAD and NAH(H). Studies in mice, which have different niacin levels compared to humans, indicated that the congenital malformations found in humans resulted from deficient NAD levels rather than increased 3-hydroxyanthranilic acid. [8] noted that NAD is a cofactor with broad cellular effects, including ATP production, macromolecular biosynthesis, redox reactions, energy metabolism, DNA repair and modulation of transcription factors, all of which play an important role in embryogenesis.[8] theorized that niacin supplementation could be of benefit in such patients.

Kynureninase catalyzes the cleavage of L-kynurenine (L-Kyn) and L-3-hydroxykynurenine (L-3OHKyn) into anthranilic acid (AA) and 3-hydroxyanthranilic acid (3-OHAA), respectively in Tryptophan metabolism. It also has cysteine-conjugate-beta-lyase activity. *KYNU* was found to be associated with Encephalopathy due to hydroxykynureninuria in the Open Target platform. None of the included variants were reported previously in either OMIM or the mGWAS server. rs752759030 was found to be weakly associated in the UKBB PheWAS with traits such as ever had stillbirth, spontaneous miscarriage or termination.

CCBL1 was associated with increased levels of indolelactate in a burden test that included 17 rare variants. Indolelactate is a tryptophan metabolite found in human plasma and serum and normal urine. Tryptophan is metabolized by two major pathways in humans, either through kynurenine or via a series of indoles. CCBL1 (aka KYAT1) is part of the Tryptophan catabolism pathway. Cysteine conjugate beta-lyase 1 (CCBL1; glutamine transaminase K; kynurenine aminotransferase; EC 2.6.1.64) metabolizes cysteine conjugates of certain halogenated alkenes and alkanes to form reactive metabolites that can produce nephrotoxicity and neurotoxicity in experimental animals and human. Indolelactate is a regulator of the reaction catalyzed by CCBL1.

Both KYNU and CCBL1 are enzymes of the kynurenine pathway which is often systematically up-regulated when the immune response is activated. The biological significance is that 1) the depletion of tryptophan and generation of kynurenines play a key modulatory role in the immune response; and 2) some of the kynurenines, such as quinolinic acid, 3-hydroxykynurenine and kynurenic acid, are neuroactive.

The kynurenine pathway has been demonstrated to be involved in many diseases and disorders, including Alzheimer's disease, amyotrophic lateral sclerosis, Huntington's disease, AIDS dementia complex, malaria, cancer, depression and schizophrenia, where imbalances in tryptophan and kynurenines have been found [10].

PAH was associated with increased levels of phenylalanine through a Madsen-Browning test ($P=1.7\times10^{-10}$) that contains 11 variants. This is a well-known association and many reported mutations in *PAH* are

causing Phenylketonuria (PKU; 261600) an autosomal recessive inborn error of metabolism resulting from a deficiency of the PAH enzyme. Phenylalanine hydroxylase (PAH; EC 1.14.16.1) catalyzes the hydroxylation of phenylalanine to tyrosine, the rate-limiting step in the phenylalanine catabolism. The reaction is dependent on tetrahydrobiopterin (BH4), as a cofactor, molecular oxygen and iron. If undiagnosed and untreated, phenylketonuria can result in impaired postnatal cognitive development resulting from a neurotoxic effect of hyperphenylalaninemia [11]. Today newborn screening is available for this condition and it is usually treated, maintaining a good standard of living for the patient. Four variants included in the window tested were also reported in OMIM listed variants causing PKU (rs5030860, rs62508646, rs62642937 and rs62644499). All driver variants identified are causing a missense change in the protein, specifically rs5030860 is annotated as pathogenic in ClinVar, and in the homozygous state it causes mild PKU and non-PKU hyperphenylalaninemia (HPA).

NAT8 was associated with decreased levels of N-acetyl arginine through a burden test ($P=8.4\times10^{-10}$) that collapsed 13 variants in a window. *NAT8* was also associated with N-acetyltyrosine, another acetylated amino acid. NAT8 catalyzes the last step of mercapturic acid formation by acetylating cysteine S-conjugates to mercapturic acids [12]. This gene plays an important role in the development and maintenance of normal kidney and liver structure and function. Mercapturic acid synthesis allows detoxification and excretion of cysteinyl conjugates under the form of mercapturic acids. [13] identified an association between rs13391552 in the NAT8 gene and N-acetylornithine levels with a P of 5.4×10^{-252}) that has been also reported by [14]. The N-acetyltransferase function of NAT8 matches the associating metabolite N-acetylornithine, and [13] found associations with glomerular filtration rate (GFR) and chronic kidney disease, represented by association of N-acetylornithine with estimated GFR, in this study. An alternative NAT8 function may be lysine N-acetyltransferase activity catalyzing peptidyl-lysine N6-acetylation of various proteins.

Seven lipid metabolites were associated with 5 genes (*ABCG5, SLC16A9, CERS4, RGS3, ACADS*) making this metabolic class the second most represented in the MLOF approach.

ABCG5 was associated with increased campesterol level in a burden test ($P=1.6\times10^{-08}$) that included 14 variants. The *ABCG5* gene is tandemly arrayed on chromosome 2, in a head-to-head orientation with family member *ABCG8*. ATP binding cassette subfamily G member 5 (ABCG5) encoded by *ABCG5* gene is an ABC transporter involved in the lipid homeostasis pathway transporting sterols from the cytosol to the extracellular domain. ABCG5 functions as a half-transporter to limit intestinal absorption and promote biliary excretion of sterols. It is expressed in a tissue-specific manner in the liver, colon, and intestine. Mutations in this gene may contribute to sterol accumulation and atherosclerosis, and have been observed in patients with phytosterolemia.

Campesterol is a phytosterol (PS), meaning it is a steroid derived from plants. As a food additive, phytosterols have cholesterol-lowering properties (reducing cholesterol absorption in intestines), and may act in cancer prevention. Phytosterols naturally occur in small amounts in vegetable oils, especially soybean oil. One such phytosterol complex, isolated from vegetable oil, is cholestatin, composed of campesterol, stigmasterol, and brassicasterol, and is marketed as a dietary supplement. Sterols can reduce cholesterol in human subjects by up to 15%. The mechanism behind phytosterols and the lowering of cholesterol occurs as follows: the incorporation of cholesterol into micelles in the gastrointestinal tract is inhibited, decreasing the overall amount of cholesterol absorbed. This may in turn help to control body total cholesterol levels, as well as modify HDL, LDL and TAG levels. Many margarines, butters, breakfast cereals and spreads are now enriched with phytosterols and marketed towards people with high cholesterol and a wish to lower it. Moreover, there are different mechanisms of action on how PS can help decreasing levels of plasma cholesterol that are not only limited to competitive solubilization into mixed micelles between cholesterol and PS at the intestinal level as described above, but also i) Esterification of free cholesterol in the enterocyte is reduced by competition with PS for ACAT-2 enzyme; Upregulation of the heterodimer ABCG5/G8 by PS can increase intestinal and hepato-biliar secretion; Upregulation of ABCA1 by PS can increase the incorporation of sterols into nascent HDL; Increased cholesterol excretion via TICE; Although it is not directly mediated by PS, the lower levels of hepatic cholesterol can lead to a lower VLDL secretion and upregulation of LDL receptor, which improves the clearance of plasma cholesterol [15]. In healthy individuals a maximum of 5% of the plant sterol intake is absorbed, resulting in very low levels in plasma

(0.5 mg dL, representing less than 0.5% total neutral sterols in plasma). However, there is a restricted group of patients affected by phytosterolemia, a rare autosomal recessive disease, that can absorb up to 60% of the dietary plant sterols whereas the rate of absorption of cholesterol seems to be normal. Phytosterolemia is characterized by mutations occurring in ABCG genes controlling the efflux of PS at intestinal level and the delivery of PS by the liver. Several studies reported that these patients have PS plasma levels from 18 to 72 mg dL, which represents 7–30% of the total neutral sterols in plasma. With this disease, there is an accumulation of PS not only in plasma but also in adipose tissue, skin, aorta and other tissues. As a result, the main symptoms of this disease include xanthomatosis and atherosclerosis. This accumulation is not only related by hyperabsorption of PS but also by impaired biliary secretion. Some authors have found that phytosterolemia patients present around 20% reduction of PS biliary excretion and around 50% reduction of the whole-body cholesterol synthesis. It has been also found that in phytosterolemic patients, the hepatic conversion of cholesterol to bile acids is blocked, which can result in cholesterol accumulation and atherosclerosis. New interest in the development of phytosterolemia has arisen from the fact that several studies found relationships between anomalous high levels of PS in plasma and CHD in non-phytosterolemic subjects. For instance, Glueck et al. [16] found that plasma cholesterol levels of 7 mmol L and 40 mmol L of PS were associated with a higher deposition of PS in the aorta in seven subjects. Likewise, Salen et al. [17] reported that lethal atherosclerosis is related to increases in plasma PS levels. Therefore, PS levels in phytosterolemia patients must be carefully controlled. A genome-wide association study for serum phytosterols conducted by Teupser et al (2010) [18] identified common variants in ABCG8 and ABO to be strongly associated with serum phytosterol levels and showed concordant and previously unknown associations with coronary artery disease (CAD). Emerging evidence suggests that PS can consequently play an important role in the prevention of several types of cancer such as lung, stomach, prostate, ovarian and breast cancer. One of the first studies suggesting the preventive effect of PS on cancer showed that Seventh-Day Adventists, having a high dietary intake of PS, presented low rates of colon cancer. The PS intake in this population could reach 344 mg day 1, which was considerably higher in comparison with the average intake of the USA population. This preventive effect was mainly attributed to the reduced bile acid excretion of this population after PS intake, as it is known that high levels of bile acids in the bowel can increase the risk of colon cancer. In contrast, contradictory results have been reported with regard to decreased bile acid excretion due to PS intake. Moreover, these findings are limited by the possible modulating effect coming from other components of the diet. Ileostomy studies are more accurate to determine their effect on reducing bile acid levels in the bowel as the variability attributed to side factors can be minimized or even eliminated. Revision of the available studies on this subject reveal that the magnitude of the effect attributed to PS on the reduction of bile acid excretion can be highly dependent on other dietary factors that must be considered. In addition, the effect on bile acid excretion can vary according to the molecule of the PS studied. Some authors have proposed that other mechanisms could be involved in the cancer preventive effect of PS. Awad and Fink [19] proposed a hypothesis based on the inhibition of cell growth through stimulation of apoptosis (programmed cell death). In vitro studies have also shown the inhibitory effect of certain PS on breast and colon-cancer cell cultures and, to a lower extent, on prostate-cancer cells. Another proposed mechanism is based on the capacity of PS to stimulate the sphingomyelin cycle. For instance, sitosterol seems to have a clear in vitro modulatory effect on this cycle. The PS molecule can be incorporated into the cell wall, thus reducing sphingomyelin and increasing ceramide levels in the cell membrane, which can consequently increase cell apoptosis. Finally, changes in testosterone concentrations can also be accounted as an alternative mechanism involved in the prevention of prostate cancer. It has also been reported that diets containing 2% PS reduce the activity of 5 a-reductase in liver and prostate and thus the testosterone levels in plasma. However, this hypothesis has not yet been confirmed in human studies.

SLC16A9 was associated with decreased levels of carnitine in a variable threshold test (9.5×10^{-09}) aggregating 13 variants in a window. Solute carrier family 16, member 9 (SLC16A9) aka Monocarboxylate transporter 9 (MTC9) belongs to a family of monocarboxylate transporters that facilitate diffusion of monocarboxylate across the plasma membrane via a proton-linked transport along a pH gradient [20]. Monocarboxylates, such as lactate and pyruvate, play a central role in cellular metabolism and metabolic communication between tissues. SLC16A9 is involved in drug transport, bile salt and organic anion transport and has been previously shown to be associated with carnitine and uric acid levels. [13] identified rs7094971 in the *SLC16A9* gene as associated with carnitine levels with a P of 3.4 x 10(-14). To follow up this finding, they tested 3H-carnitine uptake by SLC16A9-expressing Xenopus oocytes and found that

SLC16A9 is a pH-independent carnitine efflux transporter, possibly responsible for carnitine efflux from absorptive epithelia into the blood. Insight in Genome-Wide Association of Metabolite Quantitative Traits by Exome Sequence Analyses from The Erasmus Rucphen Family (ERF) study showed that in the ERF population carnitine and uric acid are highly correlated (r = 0.25, P = 3.93×10^{-13}). They found that rs1171614, located in the 5′UTR of SLC16A9, influences the lymphoblast expression of *SLC16A9* in both the GTEX and GEUVADIS databases, indicating that the effect on carnitine level is possible through expression, rather than through the change in protein function. *SLC16A9* was associated with elevated serum uric acid (SUA) levels in recent genome-wide association studies (GWAS) [21]. A missense variant of *SLC16A9* (K258T), rs2242206, was investigated by [22] in relation to gout, a common disease caused by hyperuricemia, which shows elevated serum uric acid (SUA) levels. Gout patients can be divided into those with renal overload (ROL) gout with intestinal urate underexcretion, and those with renal underexcretion (RUE) gout. rs2242206 significantly increased the risk of ROL gout (P = 0.012), with an odds ratio (OR) of 1.28 indicating decrease in intestinal urate excretion and therefore providing clues to better understand the pathophysiology of gout. [22]. In another study conducted by Kolz et al. (2009), rs12356193 within *SLC16A9* was associated with DL-carnitine (P=4.0×10^{-26}) and propionyl-L-carnitine (P=5.0×10^{-8}) concentrations, which in turn were associated with serum UA levels (P=1.4×10^{-57} and P=8.1×10^{-54}, respectively), forming a triangle between SNP, metabolites, and UA levels. These associations highlight additional pathways that are important in the regulation of serum uric acid levels and point towards novel potential targets for pharmacological intervention to prevent or treat hyperuricemia. In addition, these findings strongly support the hypothesis that transport proteins are key in regulating serum uric acid levels [23]. *SLC16A9* amongst others was also reported to be associated with serum urate concentrations in a study that combined data from >140,000 individuals of European ancestry within the Global Urate Genetics Consortium (GUGC) [21].

CERS4 was associated with decreased sphingomyelin (d18:1/20:1, d18:2/20:0) level in a burden test that included 19 variants (P=6.2×10^{-14}). CERS4 was also found associated with sphingomyelin (d18:1/18:1, d18:2/18:0) and stearoyl sphingomyelin (d18:1/18:0). Ceramide, the structural backbone of sphingolipids, is also an important signaling molecule in apoptosis, differentiation, and the cell cycle. Ceramide synthases (EC 2.3.1.24), such as CERS4, are conserved from yeast to mammals and are essential for de novo ceramide synthesis, which involves the formation of an amide linkage between a fatty acyl-CoA and a sphingoid base [24,25]. When overexpressed in cells is involved in the production of sphingolipids containing different fatty acid donors (N-linked stearoyl-(C18) or arachidoyl-(C20) ceramides) in a fumonisin B1-independent manner. [26] provided evidence of an association between activity of phospholipid transfer protein (PLTP; 172425) and coding variation in the CERS4 gene using linkage study. In human studies, the activity of PLTP (PLTPa) is associated with total cholesterol, VLDL and LDL cholesterol, and apoB level. PLTPa is positively correlated with insulin and glycosylated hemoglobin (HbA1C) and decreases in response to insulin infusion [26]. In a genome-wide association studies of European ancestry *CERS4* variant at rs2100944 was associated with higher levels of long-chain saturated fatty acids (VLSFAs) 20:0 carbons (P = 2.6×10^{-40}) indicating an inter-relationship of circulating VLSFAs and sphingolipid synthesis.

RGS3 was associated with increase in stearoyl sphingomyelin (d18:1/18:0) level - already reported in this study to be associated with *CERS4* but with opposite direction of effect than *RGS3* - in a variable threshold test (P=1.6×10^{-08}) that included 10 variants. RGS3 is part of what is defined as a 'regulator of G protein signaling' (RGS) domain. Proteins containing the RGS domain constitute a family of molecules that appear to function as negative regulators of heterotrimeric G protein signaling. RGS3 Down-regulates signaling from heterotrimeric G-proteins by increasing the GTPase activity of the alpha subunit, thereby driving them into their inactive GDP-bound form. RGS3 inhibits signaling through the sphingosine 1-phosphate S1P receptor subtypes 1, 2, and 3 [27].

ACADS was associated with increased butyrylcarnitine and ethylmalonate levels in a SKAT test (P=1.2×10^{-08} and P=4.7×10^{-09}, respectively). Out of the 11 variants included in the test 2 and 4 were associated with a slight reduction in ethylmalonate and butyrylcarnitine indicating the value of using a regression-based model to identify cases where a window contains a number of non-causal or opposite directions of effect. ACADS is aka Butyryl-CoA dehydrogenase and has a biochemical consequence on butyrylcarnitine level that is elevated in patients with acyl-CoA dehydrogenase, short-chain (SCAD) deficiency. SCAD deficiency is an autosomal recessive metabolic disorder of fatty acid beta-oxidation with a prevalence of 1 in 35,000 to 50,000 new-borns. In infants, it causes acute acidosis and generalized muscle weakness and in middle-

aged patients, it causes chronic myopathy localized in muscle. SCAD deficiency is generalized in the former type and localized to skeletal muscles in the latter (OMIM 201470). SCAD prevents the body from converting certain fats into energy, especially during periods without food (fasting). Van Maldegem et al. (2006) found on at least two occasions, increased butyrylcarnitine (C4) concentrations in plasma or bloodspot, and/or increased ethylmalonic acid (EMA) concentrations in urine under non-stressed conditions [28]

Two carbohydrate metabolites were associated with 2 genes (*NPL, SLC5A10*) that recapitulates the biochemical reactions.

NPL was associated with increased N-acetylneuraminate level in a burden test ($P=3.2\times10^{-09}$) that included 15 variants. *NPL* encodes for N-acetylneuraminate pyruvate lyase (EC 4.1.3.3) that controls the cellular concentration of sialic acid by catalyzing the conversion of sialic acid into acylmannosamines and pyruvate [29]. The *NPL* gene encodes a member of the N-acetylneuraminate lyase sub-family that regulate cellular concentrations of N-acetylneuraminate by mediating the reversible aldol condensation between N-acetyl-d-mannosamine (ManNAc) and pyruvate to N-acetylneuraminate. This is an ancient pathway conserved to bacteria and indeed represents a therapeutic target for pathogenic bacteria in humans because of the ability of these species to utilize the carbon sources present in the mucus-rich surfaces. N-acetylneuraminate (sialic acid, NANA, Neu5Ac) is an essential component of complex carbohydrates, which play pivotal roles in recognition processes in a variety of cellular recognition and communication processes, including host-parasite interactions, where the oligosaccharide is often required for invasion, infectivity and survival of the invading organism in the host. N-acetylneuraminate analogues therefore represent attractive targets for novel chemotherapeutic agents against bacterial and viral infections. Thus, there is an obvious link between the *NPL* gene with N-acetylneuraminate availability and suggest that *NPL* genetic variation can have strong influences on susceptibility to bacterial and viral pathogenicity.

SLC5A10 was associated with decreased 1,5-anhydroglucitol (1,5-AG) levels in a burden test ($P=9.2\times10^{-09}$) that included 15 variants. *SLC5A10* encodes for a kidney-specific sodium-dependent sugar transporter that maintains in part 1,5-anhydroglucitol level by active renal uptake. 1,5-anhydroglucitol (1,5-AG) is a non-traditional biomarker of hyperglycemia that is of growing clinical interest. It is a naturally occurring monosaccharide found in nearly all foods and absorbed in the gut. Under normoglycemic conditions, its concentrations in blood are maintained constant through renal filtration followed by reabsorption in the proximal tubules. Glucose and 1,5-AG share some transport proteins for which they represent competing substrates. When blood glucose concentrations exceed the renal glucose threshold of approximately 180 mg/dL, glucose is excreted in the urine and inhibits tubular reabsorption of 1,5-AG, resulting in lower blood 1,5-AG concentrations. Consequently, glucose peaks can lead to decreased 1,5-AG serum concentrations, and 1,5-AG has been established as a marker of hyperglycemic excursions and postprandial glucose peaks. Recent studies have demonstrated robust associations of low serum 1,5-AG concentrations with long-term microvascular and macrovascular complications in persons with diabetes, and with major cardiovascular events in persons without diabetes. These observations are supported by complementary evidence linking daily glucose fluctuations to cardiovascular complications. Gaining insights into the genetic underpinnings of a glycemic marker with unique properties, such as 1,5-AG, may improve our understanding not only of the biology of the marker itself, but also of diabetes, hyperglycemia and glucose metabolism [30]. According to the Human Protein Atlas, *SLC5A10* transcript is exclusively found in the human kidney cortex. The protein is a Na+-dependent transporter of mannose, fructose, galactose and glucose, responsible for their reabsorption from urine in the brush border of renal proximal tubule cells. Because of its exclusive expression in the kidney, genetic variation in this gene is likely related to 1,5-AG concentrations either because it also transports 1,5-AG or because it influences the amount of urinary glucose that competes with 1,5-AG for renal reuptake through *SLC5A9*. The latter protein is thought to be the main renal re-uptake mechanism for 1,5-AG. Of note, we did not observe any association between variants in SLC5A9 and 1,5-AG concentrations in our study, suggesting that SLC5A9 may not be the main transporter for renal 1,5-AG reuptake or that variants impacting SLC5A9 function were not present or detectable in our population. SLC5A9 shows high similarity to SLC5A10, suggesting that SLC5A10 may represent a novel 1,5-AG transport protein [31].

Four nucleotide metabolites involved in purine and pyrimidine metabolism were associated with 3 genes (*PTER, ADSL, UMPS*).

PTER was associated with increased N-acetyl-beta-alanine levels in a Madsen and Browning test ($P=1.9\times10^{-14}$) that included 14 variants. PTER was also associated with N-acetyltaurine in a burden test (5.5×10^{-12}). Microbial phosphotriesterases are a group of zinc metalloenzymes that catalyze the hydrolysis of a range of phosphotriester compounds. By differential cDNA library screening, [32] found that Pter was significantly underexpressed in cystic kidneys of cpk mouse, a model of human autosomal recessive polycystic kidney disease (ARPKD; 263200). Pter expression was significantly decreased upon acute renal injury induced by a single intraperitoneal injection of folic acid, and normal Pter levels returned upon recovery of kidney function. It may be involved in hydrolysing bile acids.

ADSL was associated with increased N6-succinyladenosine levels in the burden test ($P=8.3\times10^{-11}$) that included 13 variants. Adenylosuccinate lyase (ADSL) catalyzes two non-sequential steps in de novo AMP synthesis: converts (S)-2-(5-amino-1-(5-phospho-D-ribosyl)imidazole-4-carboxamido)succinate (SAICAR) to fumarate plus 5-amino-1-(5-phospho-D-ribosyl)imidazole-4-carboxamide, and thereby also contributes to de novo IMP synthesis, and converts succinyladenosine monophosphate (SAMP) to AMP and fumarate. Succinyladenosine (SAdo) is one of the dephosphorylated enzyme substrates that accumulates in body fluids of patients with adenylosuccinate lyase (ADSL) deficiency, the other being 5-amino-4-imidazole-N-succinocarboxamide riboside (SAICAr). ADSL is an inherited metabolic disease characterized by various degrees of psychomotor retardation [33]. The severity of the clinical presentation correlates with a low SAdo/SAICAr ratio in body fluids [34]. Normally Succinyladenosine is not found in blood or CSF but may be detected in trace amounts in urine. Would be interested to know how we found it in blood. And wht is the ratio SAdo/SAICAr.

UMPS was associated with increased orotate level in a SKAT test ($P=1.4\times10^{-9}$) that included 16 variants. *UMPS* encode a bifunctional enzyme with orotate phosphoribosyltransferase (OPRT) and orotidylic decarboxylase (ODC) activity, on chromosome 3q13. In mammalian cells, the last step of pyrimidine nucleotide synthesis involves the conversion of orotate to uridine monophosphate (UMP) and is catalyzed by UMP synthase [35]. This bifunctional enzyme has 2 sequential activities, orotate phosphoribosyltransferase (OPRT; EC 2.4.2.10) and orotidine-5-monophosphate decarboxylase (ODC; EC 4.1.1.23). Compound heterozygous mutation in the UMPS gene can cause orotic aciduria. Orotic aciduria is a rare autosomal recessive disorder characterized by megaloblastic anemia and orotic acid crystalluria that is frequently associated with some degree of physical and mental retardation. These features respond to appropriate pyrimidine replacement therapy, and most cases appear to have a good prognosis. A minority of cases have additional features, particularly congenital malformations and immune deficiencies, which may adversely affect this prognosis. Webster et al. 2001 stated that only 2 cases of orotic aciduria without megaloblastic anemia (OAWA) had been reported [36]. Orotic acid is a minor dietary constituent. Indeed, until it was realized that it could be synthesized by humans, orotic acid was known as vitamin B13. The richest dietary sources are cow's milk and other dairy products as well as root vegetables such as carrots and beets. Dietary intake probably contributes to a basal rate of orotic acid excretion in urine because fasting decreases excretion by ~50%. However, it is now apparent that most urinary orotic acid is synthesized in the body, where it arises as an intermediate in the pathway for the synthesis of pyrimidine nucleotides. Orotic acid is converted to UMP by UMP synthase, a multifunctional protein with both orotate phosphoribosyltransferase and orotidylate decarboxylase activity. The most frequently observed inborn error of pyrimidine nucleotide synthesis is a mutation of the multifunctional protein UMP synthase. This disorder prevents the conversion of orotic acid to UMP and thus to other pyrimidines. As a result, plasma orotic acid accumulates to high concentrations, and increased quantities appear in the urine. Indeed, urinary orotic acid is so markedly increased in individuals harboring a mutation in UMP synthase that orotic acid crystals can form in the urine. The urinary concentration of orotic acid in homozygotes can be of the order of millimoles per millimole creatinine. By comparison, the urinary level in unaffected individuals is ~ 1 umol/mmol creatinine [37]. Potential for therapeutic uses. Pyrimidine synthesis inhibitors are used in active moderate to severe rheumatoid arthritis and psoriatic arthritis, as well as in multiple sclerosis. Examples include Leflunomide and Teriflunomide. One energy metabolite involved in the TCA cycle was associated with the LACTB gene.

LACTB was associated with increased succinylcarnitine level in a variable threshold test ($P=7.6\times10^{-13}$) that included 14 variants. LACTB, is a mitochondrial serine protease that acts as a regulator of mitochondrial lipid metabolism. Acts by decreasing protein levels of PISD, a mitochondrial enzyme that converts phosphatidylserine (PtdSer) to phosphatidylethanolamine (PtdEtn), thereby affecting mitochondrial lipid metabolism [38]. Succinylcarnitine (C4DC) arises from the TCA cycle intermediate succinyl-CoA in plasma and liver. Evidence of the association of LACTB with succinylcarnitine arise from a perturbed hepatic gene expression in transgenic LACTB mice that suggests a role of LACTB in the butanoate/succinate pathway. Moreover, transgenic LACTB mice are obese [39]. Common variants in LACTB have been previously associated with high-density lipoprotein cholesterol measurement in GWAS study and also with succinylcarnitine itself [13,40]. LACTB is also a tumor suppressor that modulates lipid metabolism and cell state [38].

Five unknown metabolites were associated with 3 genes (*COMT, ABCC2, ALB*)

COMT was associated with decreased X - 11593 levels in a SKAT test ($P=9.2\times10^{-09}$) that included 8 variants. Catechol-O-methyltransferase (COMT; EC 2.1.1.6) is one of the major mammalian enzymes involved in the metabolic degradation of catecholamines [41]. COMT catalyzes the transfer of a methyl group from S-adenosyl-methionine (SAM) to a hydroxyl group on a catechol nucleus (e.g., dopamine, norepinephrine, or catechol estrogen) [42]. Catechol-O-methyltransferase (COMT) is one of several enzymes that degrade catecholamines (such as dopamine, epinephrine, and norepinephrine), catecholestrogens, and various substances having a catechol structure. Heterozygous or homozygous variants for the COMT V158M methionine allele slow down the COMT enzyme, affecting the breakdown of dopamine and stress hormones, as well as estrogen metabolism. [43] found the COMT 158val/val genotype to confer a significant risk of worse response after 4–6 weeks of antidepressant treatment in patients with major depression suggesting a potentially beneficial effect of an antidepressant add-on therapy with substances increasing dopamine availability individually tailored according to COMT val158met genotype. The molecular formula of the unnamed compound is $C_7H_{10}O_6$ and is most likely O-methylascorbate based on some previous correlations, but we are still in the process of final confirmation. O-methylascorbate is a known product of ascorbate (vitamin C) O-methylation by COMT [44]. Thus, these observations establish a link between O-methylascorbate blood levels, common genetic variation in the COMT locus, and COMT-mediated liver detoxification processes.

ABCC2 was associated with an increased X - 21467 level in a SKAT test ($P=2\times10^{-09}$) that included 16 variants. *ABCC2* was also associated with X - 21441 in a burden test ($P=2\times10^{-08}$).

ABCC2 belongs to the ATP-binding cassette transporter superfamily and transports endogenous and exogenous anionic conjugates from hepatocytes to bile. ABCC2 is also associated with Dubin-Johnson syndrome, a recessively inherited disorder of bilirubin characterized by a build-up of bilirubin in the bloodstream (hyperbilirubinemia). Within this context, X-21441 is a large compound that has precluded identification of a definitive formula that has both sulfate and glucuronide signatures. There are two formulas which work, assuming a sulfate and glucuronide conjugation, but the most likely one is $C_{27}H_{42}SO_{12}$, that supports a steroid backbone (e.g. sulfate pregnenetriol glucuronide), and likely precludes a bilirubin-associated catabolite. However, there are many combinations and further work is required to elucidate final structural determinants for this biochemical.

ALB was associated with decreased X - 22771 levels in a SKAT test ($P=2\times10^{-09}$) that included 9 variants. *ALB* was also associated with X - 16964 in a SKAT test ($P=4.5\times10^{-09}$). *ALB* is encoding for albumin, which is the major protein of the blood plasma, amounting to 60 to 65% of its total protein. The principal functions of albumin are to support the oncotic pressure, which aids in keeping the blood within the circulation, and to sequester and transport many metabolites within the body, particularly less soluble, hydrophobic ones. It is also an important circulating antioxidant and possesses enzymatic properties [45]. We could speculate that the unknown metabolite could be a byproduct of albumin. Mutation on this gene could cause analbuminemia is a rare autosomal recessive disorder manifested by the presence of a very low amount of circulating serum albumin (Negative beta from our result = -2.443 (0.407)). Affected individuals have few clinical symptoms other than mild edema, hypotension, fatigue, and, occasionally, a peculiar lower body lipodystrophy (mainly in adult females). The most common biochemical finding is a gross hyperlipidemia, with a significant increase in the total and LDL cholesterol concentrations, but normal concentrations of

HDL cholesterol and triglycerides. Analbuminemia often leads to fetal or neonatal death in siblings in families of analbuminemic subjects, which may explain the rarity of the trait [46]. Heterozygotes showed intermediate levels of serum albumin. Familial dysalbuminemic hyperthyroxinemia is an autosomal dominant condition characterized by the presence of a variant serum albumin with preferential affinity for thyroxine (T4) in clinically euthyroid individuals. Individuals have consistently elevated total T4 and elevated or normal free T4 values with normal TSH levels. The condition may be confused with hyperthyroidism or thyroid hormone resistance syndromes, prompting repeated unnecessary laboratory testing and possibly even inappropriate treatment [47].

LOF tests

ERICH6 was associated with decreased glycerophosphorylcholine (GPC) level in a variable threshold test ($P=1.9\times10^{-08}$) that included 5 variants. This gene shows a much greater number of LoF variants compared to the window means for other genes (mean number of variants = 3.056). *ERICH6* is not a well characterized gene and the association with glycerophosphorylcholine (GPC) is not trivial to interpret. All the 5 variants are LoF High confidence from Loftee, but the gene is really tolerant to LoF mutation rvis score 1.82 (97.02%). GPC rises in renal medullary cells in vivo when the medullary interstitial levels of salt and urea to which they are exposed become elevated. High NaCl increases the rate of synthesis of GPC from PC, but high urea does not. PC synthesis in mammalian kidney cells occurs through the Kennedy pathway with choline as one of the starting materials. The synthesis of GPC from PC entails the removal of fatty acids by phospholipase activity. The synthesis of GPC could involve sequential activity of a phospholipase A and a lysophospholipase or, alternatively, activity of a single phospholipase B. The GPC that results is water soluble [48].

CODING tests

CIC was associated with decreased 1-(1-enyl-stearoyl)-2-linoleoyl-GPE (P-18:0/18:2) level in a burden test ($P=2.2\times10^{-08}$) that included 18 variants. CIC is an incompletely understood tissue-specific transcriptional repressor that is highly conserved among metazoan organisms and is required for the normal development of multiple adult structures. CIC is an orthologue of the Drosophila melanogaster capicua gene and functions to transduce receptor tyrosine kinase (RTK) and ERK signaling into gene expression changes through a mechanism termed default repression, wherein CIC protein is bound to target gene promoters or enhancers and inhibits transcription in the absence of signal. Activation of RTK signaling results in the accumulation of activated ERK, which directly binds to and phosphorylates CIC protein [49]. ERK-mediated CIC phosphorylation leads to a rapid relief of repression of CIC target genes, followed by cytoplasmic CIC degradation. CIC is well conserved in mammals and recent evidence suggests that human CIC mediates RTK-dependent responses linked to deregulated growth (cell cycle control), ATP-citrate lyase phosphorylation, reactive oxygen species homeostasis, and bile acid homeostasis [50]. CIC encodes a transcriptional repressor that interacts with ATXN1 [51]. [52] reported 5 patients from 4 families with a neurodevelopmental disorder characterized by delayed development apparent in infancy or the first years of life, variable intellectual disability, often with language delay, and behavioral disorders, including autistic features, attention deficit, and hyperactivity. Some patients showed developmental regression early in life and/or learning difficulties. Three patients had seizures, including blinking and staring episodes, myoclonic seizures, complex partial seizures, and absence seizures. Two patients had hypotonia, and 3 had nonspecific T2-weighted white matter abnormalities on brain imaging. Since this gene encodes a DNA binding protein and not an enzyme, the genetic association with the plasmalogen 1-(1-enyl-stearoyl)-2-linoleoyl-GPE (P-18:0/18:2)* is less tractable and can involve regulation of a number of genes in the synthesis and degradation of plasmalogens [53]. However, future work can focus on the specific set of genes that CIC could potentially regulate in plasmalogen synthesis. Perhaps it could be due to the neighboring gene platelet activating factor acetylhydrolase 1b catalytic subunit 3 which has a role in hydrolyzing phospholipids at the golgi.

TYMP was associated with increased 5-methyluridine level in a SKAT test ($P=3.4\times10^{-09}$) that included 10 variants. *TYMP* encodes thymidine phosphorylase (EC 2.4.2.4), a cytosolic enzyme that catalyzes the phosphorylation of thymidine or deoxyuridine to thymine or uracil, and is thus essential for the nucleotide salvage pathway [54]. The protein product was originally identified as platelet-derived endothelial cell growth

factor (PDECGF), an angiogenic factor distinct from the previously described endothelial cell mitogens of the fibroblast growth factor family [55]. PDECGF is stored in platelets as a 45-kD single polypeptide chain and has a highly restricted target cell specificity acting only on endothelial cells. It promotes angiogenesis in vivo, and stimulates the in vitro growth of a variety of endothelial cells. Homozygous or compound heterozygous mutation in the nuclear-encoded TYMP on chromosome 22q13 causes mitochondrial DNA depletion syndrome-1 (MTDPS1), which manifests as a neurogastrointestinal encephalopathy (MNGIE). Mitochondrial DNA depletion syndrome-1 (MTDPS1) is an autosomal recessive progressive multisystem disorder clinically characterized by onset between the second and fifth decades of life of ptosis, progressive external ophthalmoplegia (PEO), gastrointestinal dysmotility (often pseudo obstruction), cachexia, diffuse leukoencephalopathy, peripheral neuropathy, and mitochondrial dysfunction. Mitochondrial DNA abnormalities can include depletion, deletion, and point mutations [56].

CHKB was associated with an increased 5-methyluridine level in a SKAT test ($P=4.8\times10^{-09}$) that included 14 variants. CHKB encoded for choline kinases (EC 2.7.1.32) that catalyze phosphorylation of choline by ATP in the presence of Mg(2+), yielding phosphocholine and ADP. This step commits choline to the enzymatic pathway for biosynthesis of phosphatidylcholine [57,58]. Choline kinase in mammals is encoded by two genes, CHKA and CHKB. These enzymes catalyze the phosphorylation of choline and ethanolamine to phosphocholine and phosphoethanolamine. As a class, these are the first enzymes in the biosynthesis of phosphatidylcholine and phosphatidylethanolamine in all animal cells. Disruption of murine CHKA leads to embryonic lethality, whereas a spontaneous genomic deletion in murine CHKB results in neonatal forelimb bone deformity and hindlimb muscular dystrophy. Studies have found that CHKB is the major isoform in murine hindlimb muscle and contributes more to choline kinase activity. 5-methyluridine, also called ribothymidine, is an endogenous methylated nucleoside resulting from the m5U methyltransferase from uridine substrate with SAM as the methyl donor. Choline and pyrimidine metabolism are required components of phospholipid metabolism, thus genetic association of CHKB with 5-methyluridine may be due to perturbation of cytidine and CTP utilization in phosphatidylcholine synthesis.

CR1L was associated with decreased X - 21444 level in a Madsen and Browning test ($P=1.8\times10^{-08}$) that included 11 variants. The C3b/C4b receptor (CR1) is important in immune complex processing. On phagocytic cells it promotes the adherence and sometimes the endocytosis of C3b- and C4b-coated particles. CR1 is composed largely of a tandemly repeated motif of approximately 60 amino acids, the short consensus repeat (SCR). In the course of cloning genomic CR1 sequences, [59] identified a related gene, CR1L. Analysis of subclones identified 10 exons encoding a signal peptide and SCR1 through SCR6 and SCR9, which are 91% homologous to the corresponding N-terminal regions of the CR1 protein. [59] concluded that CR1L arose by a gene duplication event and has undergone mutation at the beginning of SCR1. The unnamed metabolite has a very tentative formula of C4H6O3 with a compound at 102m/z. We will use the information of an association with the complement component (3b/4b) receptor 1-like gene to guide us in retiring this compound, but there is insufficient information to come up with rational annotation for this molecule.

SLC25A15 was associated with decreased X - 15728 levels in a burden test ($P=4.8\times10^{-09}$) that included 18 variants. SLC25A15 encodes the mitochondrial ornithine transporter, which transports ornithine across the inner mitochondrial membrane from the cytosol to the mitochondrial matrix. This is a vital step in the urea cycle, which serves to eliminate toxic ammonium ions from the breakdown of nitrogen [60]. [61] reported a child with mental retardation and myoclonic seizures associated with hyperornithinemia, hyperammonemia, and homocitrullinemia. The findings were consistent with an inherited disorder of amino acid metabolism. The protein transports ornithine across the inner mitochondrial membrane, from the cytoplasm to the matrix. Metabolite likely to be related to ornithine, citrulline of urea cycle.

DPRC1 was associated with an increased 2-aminobutyrate level in a burden test ($P=2.5\times10^{-09}$) that included 16 variants. The *DPCR1* gene was mapped to the HLA class I region and subsequently linked among other 8 genes to diffuse panbronchiolitis (DPB). Diffuse panbronchiolitis (DPB) is a rare chronic inflammatory obstructive pulmonary disease primarily affecting the respiratory bronchioles [62].

In summary, we identified a total of 6 genes associated with 13 metabolites classified as amino acids, 3 as nucleotides with 4 metabolites, 5 as lipids with 7 metabolites, 2 as carbohydrate 2 metabolites, 1 as energy, 3 as unknown 5 metabolites.

Acknowledgements

This research was supported by (1) Wellcome Trust Grant number 206194. For the purpose of Open Access, the author has applied a CC BY public copyright license to any Author Accepted Manuscript version arising from this submission; (2) Open Targets; (3) Participants in the INTERVAL randomized controlled trial were recruited with the active collaboration of NHS Blood and Transplant England (www.nhsbt.nhs.uk [nhsbt.nhs.uk]), which has supported field work and other elements of the trial. DNA extraction and genotyping were co-funded by the National Institute for Health Research (NIHR), the NIHR BioResource (http://bioresource.nihr.ac.uk [bioresource.nihr.ac.uk]) and the NIHR Cambridge Biomedical Research Center (BRC-1215-20014) [*]. Sequencing of the INTERVAL data was supported by the Wellcome Trust grant number 206194. The academic coordinating center for INTERVAL was supported by core funding from the: NIHR Blood and Transplant Research Unit in Donor Health and Genomics (NIHR BTRU-2014-10024), UK Medical Research Council (MR/L003120/1), British Heart Foundation (SP/09/002; RG/13/13/30194; RG/18/13/33946) and NIHR Cambridge BRC (BRC-1215-20014) [*]. A complete list of the investigators and contributors to the INTERVAL trial is provided in reference [23]**. The academic coordinating center would like to thank blood donor center staff and blood donors for participating in the INTERVAL trial.; (4) Health Data Research UK, which is funded by the UK Medical Research Council, Engineering and Physical Sciences Research Council, Economic and Social Research Council, Department of Health and Social Care (England), Chief Scientist Office of the Scottish Government Health and Social Care Directorates, Health and Social Care Research and Development Division (Welsh Government), Public Health Agency (Northern Ireland), British Heart Foundation and Wellcome. (5) Metabolon Metabolomics assays were funded by the NIHR BioResource and the NIHR Cambridge Biomedical Research Center (BRC-1215-20014) [*]. (6) SomaLogic assays were funded by Merck and the NIHR Cambridge Biomedical Research Center (BRC-1215-20014) [*]. (7) Nightingale Health NMR assays were funded by the European Commission Framework Programme 7 (HEALTH-F2-2012-279233). (8) Professor John Danesh holds a British Heart Foundation Professorship and a NIHR Senior Investigator Award [*].
*The views expressed are those of the author(s) and not necessarily those of the NIHR or the Department of Health and Social Care.
**Di Angelantonio E, Thompson SG, Kaptoge SK, Moore C, Walker M, Armitage J, Ouwehand WH, Roberts DJ, Danesh J, INTERVAL Trial Group. Efficiency and safety of varying the frequency of whole blood donation (INTERVAL): a randomized trial of 45,000 donors. Lancet. 2017 Nov 25;390(10110):2360-2371.[63]
We would like to thank Lorenz Wernisch for useful discussions and statistical advice, and we would like to thank three anonymous reviewers for insightful suggestions.

Supplemental Figures and Legends

Figure S1: Distribution of number of variants per window in each strategy.

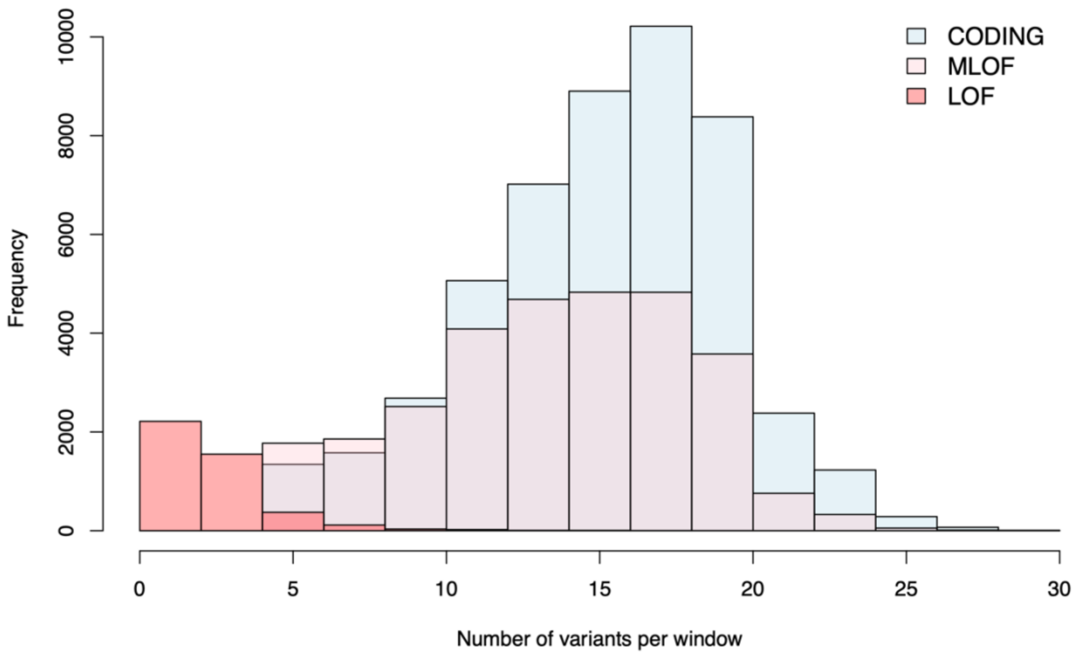

Figure S2: a) Number of driver variants shared using a forward selection approach and Lasso and b) percentage of total number of variants.

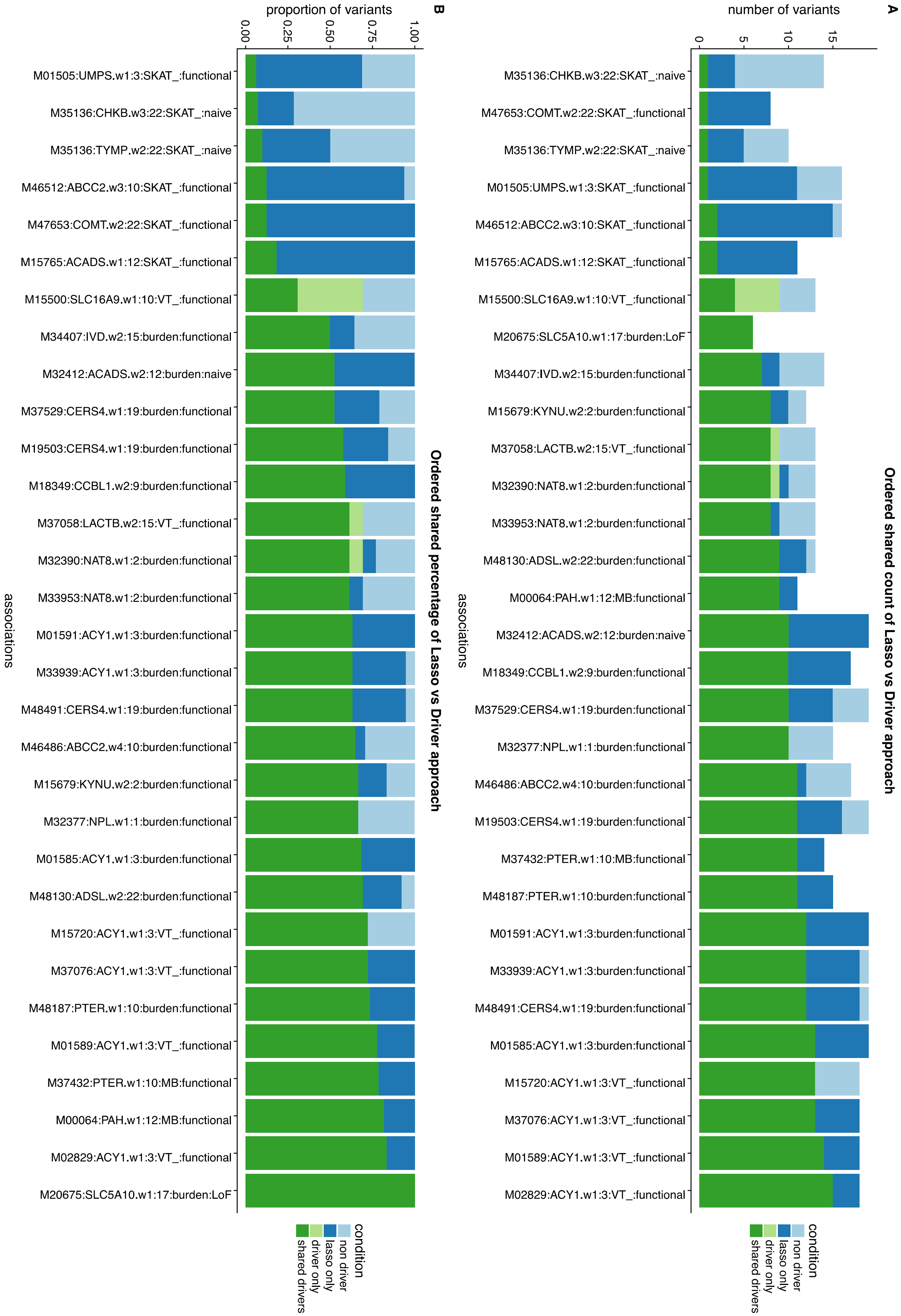
A
Ordered shared count of Lasso vs Driver approach
number of variants
0
5
10
15
associations
M35136:CHKB.w3:22:SKAT_:naive
M47653:COMT.w2:22:SKAT_:functional
M35136:TYMP.w2:22:SKAT_:naive
M01505:UMPS.w1:3:SKAT_:functional
M46512:ABCC2.w3:10:SKAT_:functional
M15765:ACADS.w1:12:SKAT_:functional
M15500:SLC16A9.w1:10:VT_:functional
M20675:SLC5A10.w1:17:burden:LoF
M34407:IVD.w2:15:burden:functional
M15679:KYNU.w2:2:burden:functional
M37058:LACTB.w2:15:VT_:functional
M32390:NAT8.w1:2:burden:functional
M33953:NAT8.w1:2:burden:functional
M48130:ADSL.w2:22:burden:functional
M00064:PAH.w1:12:MB:functional
M32412:ACADS.w2:12:burden:naive
M18349:CCBL1.w2:9:burden:functional
M37529:CERS4.w1:19:burden:functional
M32377:NPL.w1:1:burden:functional
M46486:ABCC2.w4:10:burden:functional
M19503:CERS4.w1:19:burden:functional
M37432:PTER.w1:10:MB:functional
M48187:PTER.w1:10:burden:functional
M01591:ACY1.w1:3:burden:functional
M33939:ACY1.w1:3:burden:functional
M48491:CERS4.w1:19:burden:functional
M01585:ACY1.w1:3:burden:functional
M15720:ACY1.w1:3:VT_:functional
M37076:ACY1.w1:3:VT_:functional
M01589:ACY1.w1:3:VT_:functional
M02829:ACY1.w1:3:VT_:functional
condition
non driver
lasso only
driver only
shared drivers
B
Ordered shared percentage of Lasso vs Driver approach
proportion of variants
0.00
0.25
0.50
0.75
1.00
associations
M01505:UMPS.w1:3:SKAT_:functional
M35136:CHKB.w3:22:SKAT_:naive
M35136:TYMP.w2:22:SKAT_:naive
M46512:ABCC2.w3:10:SKAT_:functional
M47653:COMT.w2:22:SKAT_:functional
M15765:ACADS.w1:12:SKAT_:functional
M15500:SLC16A9.w1:10:VT_:functional
M34407:IVD.w2:15:burden:functional
M32412:ACADS.w2:12:burden:naive
M37529:CERS4.w1:19:burden:functional
M19503:CERS4.w1:19:burden:functional
M18349:CCBL1.w2:9:burden:functional
M37058:LACTB.w2:15:VT_:functional
M32390:NAT8.w1:2:burden:functional
M33953:NAT8.w1:2:burden:functional
M01591:ACY1.w1:3:burden:functional
M33939:ACY1.w1:3:burden:functional
M48491:CERS4.w1:19:burden:functional
M46486:ABCC2.w4:10:burden:functional
M15679:KYNU.w2:2:burden:functional
M32377:NPL.w1:1:burden:functional
M01585:ACY1.w1:3:burden:functional
M48130:ADSL.w2:22:burden:functional
M15720:ACY1.w1:3:VT_:functional
M37076:ACY1.w1:3:VT_:functional
M48187:PTER.w1:10:burden:functional
M01589:ACY1.w1:3:VT_:functional
M37432:PTER.w1:10:MB:functional
M00064:PAH.w1:12:MB:functional
M02829:ACY1.w1:3:VT_:functional
M20675:SLC5A10.w1:17:burden:LoF
condition
non driver
lasso only
driver only
shared drivers

Supplemental tables

Table S1: Study participant characteristics expressed in counts and percentages.

Table S2: Linear regressions of the first five principal components against age, sex, BMI, current smoking, alcohol consumption frequency, center, batch, plate, appointment month and time between appointment and processing.

Table S3: Study metabolite counts per pathway categories.

Table S4: Summary statistics of different strategies used in the study such as number of genes tested in each strategy.

Table S5: Table of all gene-metabolite associations including variants used in the test, type of test and strategy used, whether variants were drivers or not, recalculated Ps when including only driver variants, gene reported in previous mGWAS and OMIM annotation. Also includes overall association results for window-based and gene-based analysis.

Table S6: WGS replications of discovery signals.

Table S7: Summary statistics of associations of previously identified genes (Metabolon, this study) with NMR measured metabolites levels.

Table S8: Conditional analysis of RV discoveries with sentinel variants from imputed data.

Table S9: Biological annotation of rare variant test (RVT) signals

Table S10: Details of all associations discovered based on strategies and test categories.

Supplemental References

1. Maceyka, M., Nava, V.E., Milstien, S., and Spiegel, S. (2004). Aminoacylase 1 is a sphingosine kinase 1-interacting protein. FEBS Lett. *568*, 30–34.

2. Shi, H., Hayes, M.T., Kirana, C., Miller, R.J., Keating, J.P., and Stubbs, R.S. (2013). Overexpression of aminoacylase 1 is associated with colorectal cancer progression. Hum. Pathol. *44*, 1089–1097.

3. Vockley, J., Parimoo, B., and Tanaka, K. (1991). Molecular characterization of four different classes of mutations in the isovaleryl-CoA dehydrogenase gene responsible for isovaleric acidemia. Am. J. Hum. Genet. *49*, 147–157.

4. Kılıç, M., Kaymaz, N., and Özgül, R.K. (2014). Isovaleric acidemia presenting as diabetic ketoacidosis: a case report. J. Clin. Res. Pediatr. Endocrinol. *6*, 59–61.

5. Vockley, J., and Ensenauer, R. (2006). Isovaleric acidemia: new aspects of genetic and phenotypic heterogeneity. Am. J. Med. Genet. C Semin. Med. Genet. *142C*, 95–103.

6. Vockley, J., and Ensenauer, R. (2006). Isovaleric acidemia: new aspects of genetic and phenotypic heterogeneity. Am. J. Med. Genet. C Semin. Med. Genet. *142C*, 95–103.

7. Toma, S., Nakamura, M., Toné, S., Okuno, E., Kido, R., Breton, J., Avanzi, N., Cozzi, L.,

Speciale, C., Mostardini, M., et al. (1997). Cloning and recombinant expression of rat and human kynureninase. FEBS Lett. *408*, 5–10.

8. Shi, H., Enriquez, A., Rapadas, M., Martin, E.M.M.A., Wang, R., Moreau, J., Lim, C.K., Szot, J.O., Ip, E., Hughes, J.N., et al. (2017). NAD Deficiency, Congenital Malformations, and Niacin Supplementation. N. Engl. J. Med. *377*, 544–552.

9. Komrower, G.M., Wilson, V., Clamp, J.R., and Westall, R.G. (1964). HYDROXYKYNURENINURIA: A CASE OF ABNORMAL TRYPTOPHAN METABOLISM PROBABLY DUE TO A DEFICIENCY OF KYNURENINASE. Arch. Dis. Child. *39*, 250–256.

10. Chen, Y., and Guillemin, G.J. (2009). Kynurenine pathway metabolites in humans: disease and healthy States. Int. J. Tryptophan Res. *2*, 1–19.

11. Zurflüh, M.R., Zschocke, J., Lindner, M., Feillet, F., Chery, C., Burlina, A., Stevens, R.C., Thöny, B., and Blau, N. (2008). Molecular genetics of tetrahydrobiopterin-responsive phenylalanine hydroxylase deficiency. Hum. Mutat. *29*, 167–175.

12. Veiga-da-Cunha, M., Tyteca, D., Stroobant, V., Courtoy, P.J., Opperdoes, F.R., and Van Schaftingen, E. (2010). Molecular identification of NAT8 as the enzyme that acetylates cysteine S-conjugates to mercapturic acids. J. Biol. Chem. *285*, 18888–18898.

13. Suhre, K., Shin, S.-Y., Petersen, A.-K., Mohney, R.P., Meredith, D., Wägele, B., Altmaier, E., CARDIoGRAM, Deloukas, P., Erdmann, J., et al. (2011). Human metabolic individuality in biomedical and pharmaceutical research. Nature *477*, 54–60.

14. Shin, S.-Y., Fauman, E.B., Petersen, A.-K., Krumsiek, J., Santos, R., Huang, J., Arnold, M., Erte, I., Forgetta, V., Yang, T.-P., et al. (2014). An atlas of genetic influences on human blood metabolites. Nat. Genet. *46*, 543–550.

15. Santas, J., Codony, R., and Rafecas, M. (2013). Phytosterols: Beneficial Effects. In Natural Products, K.G. Ramawat, and J.-M. Mérillon, eds. (Berlin, Heidelberg: Springer Berlin Heidelberg), pp. 3437–3464.

16. Glueck, C.J., Streicher, P., and Illig, E. (1992). Serum and dietary phytosterols, cholesterol, and coronary heart disease in hyperphytosterolemic probands. Clin. Biochem. *25*, 331–334.

17. Salen, G., Horak, I., Rothkopf, M., Cohen, J.L., Speck, J., Tint, G.S., Shore, V., Dayal, B., Chen, T., and Shefer, S. (1985). Lethal atherosclerosis associated with abnormal plasma and tissue sterol composition in sitosterolemia with xanthomatosis. J. Lipid Res. *26*, 1126–1133.

18. Teupser, D., Baber, R., Ceglarek, U., Scholz, M., Illig, T., Gieger, C., Holdt, L.M., Leichtle, A., Greiser, K.H., Huster, D., et al. (2010). Genetic regulation of serum phytosterol levels and risk of coronary artery disease. Circ. Cardiovasc. Genet. *3*, 331–339.

19. Awad, A.B., and Fink, C.S. (2000). Phytosterols as anticancer dietary components: evidence and mechanism of action. J. Nutr. *130*, 2127–2130.

20. Halestrap, A.P., and Price, N.T. (1999). The proton-linked monocarboxylate transporter (MCT) family: structure, function and regulation. Biochem. J *343 Pt 2*, 281–299.

21. Köttgen, A., Albrecht, E., Teumer, A., Vitart, V., Krumsiek, J., Hundertmark, C., Pistis, G., Ruggiero, D., O'Seaghdha, C.M., Haller, T., et al. (2013). Genome-wide association analyses

identify 18 new loci associated with serum urate concentrations. Nat. Genet. *45*, 145–154.

22. Nakayama, A., Matsuo, H., Shimizu, T., Ogata, H., Takada, Y., Nakashima, H., Nakamura, T., Shimizu, S., Chiba, T., Sakiyama, M., et al. (2013). Common missense variant of monocarboxylate transporter 9 (MCT9/SLC16A9) gene is associated with renal overload gout, but not with all gout susceptibility. Hum. Cell *26*, 133–136.

23. Kolz, M., Johnson, T., Sanna, S., Teumer, A., Vitart, V., Perola, M., Mangino, M., Albrecht, E., Wallace, C., Farrall, M., et al. (2009). Meta-analysis of 28,141 individuals identifies common variants within five new loci that influence uric acid concentrations. PLoS Genet. *5*, e1000504.

24. Riebeling, C., Allegood, J.C., Wang, E., Merrill, A.H., Jr, and Futerman, A.H. (2003). Two mammalian longevity assurance gene (LAG1) family members, trh1 and trh4, regulate dihydroceramide synthesis using different fatty acyl-CoA donors. J. Biol. Chem. *278*, 43452–43459.

25. Venkataraman, K., and Futerman, A.H. (2002). Do longevity assurance genes containing Hox domains regulate cell development via ceramide synthesis? FEBS Lett. *528*, 3–4.

26. Rosenthal, E.A., Ronald, J., Rothstein, J., Rajagopalan, R., Ranchalis, J., Wolfbauer, G., Albers, J.J., Brunzell, J.D., Motulsky, A.G., Rieder, M.J., et al. (2011). Linkage and association of phospholipid transfer protein activity to LASS4. J. Lipid Res. *52*, 1837–1846.

27. Brinkmann, V. (2007). Sphingosine 1-phosphate receptors in health and disease: mechanistic insights from gene deletion studies and reverse pharmacology. Pharmacol. Ther. *115*, 84–105.

28. van Maldegem, B.T., Duran, M., Wanders, R.J.A., Niezen-Koning, K.E., Hogeveen, M., Ijlst, L., Waterham, H.R., and Wijburg, F.A. (2006). Clinical, biochemical, and genetic heterogeneity in short-chain acyl-coenzyme A dehydrogenase deficiency. JAMA *296*, 943–952.

29. Wu, M., Gu, S., Xu, J., Zou, X., Zheng, H., Jin, Z., Xie, Y., Ji, C., and Mao, Y. (2005). A novel splice variant of human gene NPL, mainly expressed in human liver, kidney and peripheral blood leukocyte. DNA Seq. *16*, 137–142.

30. Dungan, K.M. (2008). 1,5-anhydroglucitol (GlycoMark) as a marker of short-term glycemic control and glycemic excursions. Expert Rev. Mol. Diagn. *8*, 9–19.

31. Long, T., Hicks, M., Yu, H.-C., Biggs, W.H., Kirkness, E.F., Menni, C., Zierer, J., Small, K.S., Mangino, M., Messier, H., et al. (2017). Whole-genome sequencing identifies common-to-rare variants associated with human blood metabolites. Nat. Genet. *49*, 568–578.

32. Hou, X., Maser, R.L., Magenheimer, B.S., and Calvet, J.P. (1996). A mouse kidney- and liver-expressed cDNA having homology with a prokaryotic parathion hydrolase (phosphotriesterase)-encoding gene: abnormal expression in injured and polycystic kidneys. Gene *168*, 157–163.

33. Zikánová, M., Krijt, J., Hartmannová, H., and Kmoch, S. (2005). Preparation of 5-amino-4-imidazole-N-succinocarboxamide ribotide, 5-amino-4-imidazole-N-succinocarboxamide riboside and succinyladenosine, compounds usable in diagnosis and research of adenylosuccinate lyase deficiency. J. Inherit. Metab. Dis. *28*, 493–499.

34. Marinaki, A.M., Champion, M., Kurian, M.A., Simmonds, H.A., Marie, S., Vincent, M.F., van den Berghe, G., Duley, J.A., and Fairbanks, L.D. (2004). Adenylosuccinate lyase deficiency--first British case. Nucleosides Nucleotides Nucleic Acids *23*, 1231–1233.

35. McClard, R.W., Black, M.J., Livingstone, L.R., and Jones, M.E. (1980). Isolation and initial characterization of the single polypeptide that synthesizes uridine 5'-monophosphate from orotate in Ehrlich ascites carcinoma. Purification by tandem affinity chromatography of uridine-5'-monophosphate synthase. Biochemistry *19*, 4699–4706.

36. Bailey, C.J. (2009). Orotic aciduria and uridine monophosphate synthase: a reappraisal. J. Inherit. Metab. Dis. *32 Suppl 1*, S227–S233.

37. Brosnan, M.E., and Brosnan, J.T. (2007). Orotic acid excretion and arginine metabolism. J. Nutr. *137*, 1656S – 1661S.

38. Keckesova, Z., Donaher, J.L., De Cock, J., Freinkman, E., Lingrell, S., Bachovchin, D.A., Bierie, B., Tischler, V., Noske, A., Okondo, M.C., et al. (2017). LACTB is a tumour suppressor that modulates lipid metabolism and cell state. Nature *543*, 681–686.

39. Yang, X., Deignan, J.L., Qi, H., Zhu, J., Qian, S., Zhong, J., Torosyan, G., Majid, S., Falkard, B., Kleinhanz, R.R., et al. (2009). Validation of candidate causal genes for obesity that affect shared metabolic pathways and networks. Nat. Genet. *41*, 415–423.

40. Willer, C.J., Schmidt, E.M., Sengupta, S., Peloso, G.M., Gustafsson, S., Kanoni, S., Ganna, A., Chen, J., Buchkovich, M.L., Mora, S., et al. (2013). Discovery and refinement of loci associated with lipid levels. Nat. Genet. *45*, 1274–1283.

41. Gogos, J.A., Morgan, M., Luine, V., Santha, M., Ogawa, S., Pfaff, D., and Karayiorgou, M. (1998). Catechol-O-methyltransferase-deficient mice exhibit sexually dimorphic changes in catecholamine levels and behavior. Proc. Natl. Acad. Sci. U. S. A. *95*, 9991–9996.

42. Chen, J., Lipska, B.K., Halim, N., Ma, Q.D., Matsumoto, M., Melhem, S., Kolachana, B.S., Hyde, T.M., Herman, M.M., Apud, J., et al. (2004). Functional analysis of genetic variation in catechol-O-methyltransferase (COMT): effects on mRNA, protein, and enzyme activity in postmortem human brain. Am. J. Hum. Genet. *75*, 807–821.

43. Baune, B.T., Hohoff, C., Berger, K., Neumann, A., Mortensen, S., Roehrs, T., Deckert, J., Arolt, V., and Domschke, K. (2008). Association of the COMT val158met variant with antidepressant treatment response in major depression. Neuropsychopharmacology *33*, 924–932.

44. Bowers-Komro, D.M., McCormick, D.B., King, G.A., Sweeny, J.G., and Iacobucci, G.A. (1982). Confirmation of 2-O-methyl ascorbic acid as the product from the enzymatic methylation of L-ascorbic acid by catechol-O-methyltransferase. Int. J. Vitam. Nutr. Res. *52*, 186–193.

45. Minchiotti, L., Galliano, M., Kragh-Hansen, U., and Peters, T., Jr (2008). Mutations and polymorphisms of the gene of the major human blood protein, serum albumin. Hum. Mutat. *29*, 1007–1016.

46. Caridi, G., Maout, A., Artan, R., Campagnoli, M., Lugani, F., Abada, M.E.A., Sayar, E., Galliano, M., and Minchiotti, L. (2018). Congenital Analbuminemia in Unrelated Algerian and Turkish Families is Caused by the Same Molecular Defect in the Albumin Gene. Ann. Lab. Med.

38, 185–188.

47. Heufelder, A.E., Klee, G.G., Wynne, A.G., and Gharib, H. (1995). Familial dysalbuminemic hyperthyroxinemia: cumulative experience in 29 consecutive patients. Endocr. Pract. *1*, 4–8.

48. Gallazzini, M., and Burg, M.B. (2009). What's new about osmotic regulation of glycerophosphocholine. Physiology *24*, 245–249.

49. Astigarraga, S., Grossman, R., Díaz-Delfín, J., Caelles, C., Paroush, Z. 'ev, and Jiménez, G. (2007). A MAPK docking site is critical for downregulation of Capicua by Torso and EGFR RTK signaling. EMBO J. *26*, 668–677.

50. Jiménez, G., Shvartsman, S.Y., and Paroush, Z. 'ev (2012). The Capicua repressor--a general sensor of RTK signaling in development and disease. J. Cell Sci. *125*, 1383–1391.

51. Bergeron, D., Lapointe, C., Bissonnette, C., Tremblay, G., Motard, J., and Roucou, X. (2013). An out-of-frame overlapping reading frame in the ataxin-1 coding sequence encodes a novel ataxin-1 interacting protein. J. Biol. Chem. *288*, 21824–21835.

52. Lu, H.-C., Tan, Q., Rousseaux, M.W.C., Wang, W., Kim, J.-Y., Richman, R., Wan, Y.-W., Yeh, S.-Y., Patel, J.M., Liu, X., et al. (2017). Disruption of the ATXN1-CIC complex causes a spectrum of neurobehavioral phenotypes in mice and humans. Nat. Genet. *49*, 527–536.

53. Braverman, N.E., and Moser, A.B. (2012). Functions of plasmalogen lipids in health and disease. Biochim. Biophys. Acta *1822*, 1442–1452.

54. Suomalainen, A., and Isohanni, P. (2010). Mitochondrial DNA depletion syndromes--many genes, common mechanisms. Neuromuscul. Disord. *20*, 429–437.

55. Ishikawa, F., Miyazono, K., Hellman, U., Drexler, H., Wernstedt, C., Hagiwara, K., Usuki, K., Takaku, F., Risau, W., and Heldin, C.H. (1989). Identification of angiogenic activity and the cloning and expression of platelet-derived endothelial cell growth factor. Nature *338*, 557–562.

56. Taanman, J.-W., Daras, M., Albrecht, J., Davie, C.A., Mallam, E.A., Muddle, J.R., Weatherall, M., Warner, T.T., Schapira, A.H.V., and Ginsberg, L. (2009). Characterization of a novel TYMP splice site mutation associated with mitochondrial neurogastrointestinal encephalomyopathy (MNGIE). Neuromuscul. Disord. *19*, 151–154.

57. Ishidate, K. (1997). Choline/ethanolamine kinase from mammalian tissues. Biochim. Biophys. Acta *1348*, 70–78.

58. Aoyama, C., Yamazaki, N., Terada, H., and Ishidate, K. (2000). Structure and characterization of the genes for murine choline/ethanolamine kinase isozymes alpha and beta. J. Lipid Res. *41*, 452–464.

59. Hourcade, D., Miesner, D.R., Bee, C., Zeldes, W., and Atkinson, J.P. (1990). Duplication and divergence of the amino-terminal coding region of the complement receptor 1 (CR1) gene. An example of concerted (horizontal) evolution within a gene. J. Biol. Chem. *265*, 974–980.

60. Camacho, J.A., Obie, C., Biery, B., Goodman, B.K., Hu, C.A., Almashanu, S., Steel, G., Casey, R., Lambert, M., Mitchell, G.A., et al. (1999). Hyperornithinaemia-hyperammonaemia-homocitrullinuria syndrome is caused by mutations in a gene encoding a mitochondrial ornithine transporter. Nat. Genet. *22*, 151–158.

61. Shih, V.E., Laframboise, R., Mandell, R., and Pichette, J. (1992). Neonatal form of the hyperornithinaemia, hyperammonaemia, and homocitrullinuria (HHH) syndrome and prenatal diagnosis. Prenat. Diagn. *12*, 717–723.

62. Matsuzaka, Y., Tounai, K., Denda, A., Tomizawa, M., Makino, S., Okamoto, K., Keicho, N., Oka, A., Kulski, J.K., Tamiya, G., et al. (2002). Identification of novel candidate genes in the diffuse panbronchiolitis critical region of the class I human MHC. Immunogenetics *54*, 301–309.

63. Di Angelantonio, E., Thompson, S.G., Kaptoge, S., Moore, C., Walker, M., Armitage, J., Ouwehand, W.H., Roberts, D.J., Danesh, J., and INTERVAL Trial Group (2017). Efficiency and safety of varying the frequency of whole blood donation (INTERVAL): a randomised trial of 45 000 donors. Lancet *390*, 2360–2371.
