# Supplementary material for: Whole-exome sequencing identifies rare genetic variants associated with human plasma metabolites
Source: Am J Hum Genet. 2022 May 13;109(6):1038–54. doi: 10.1016/j.ajhg.2022.04.009 (PMC9247822; doi:10.1016/j.ajhg.2022.04.009)
Supplement: Document S2. Article plus supplemental information [file mmc12.pdf]

Whole-exome sequencing identifies rare genetic variants associated with human plasma metabolites

Graphical abstract

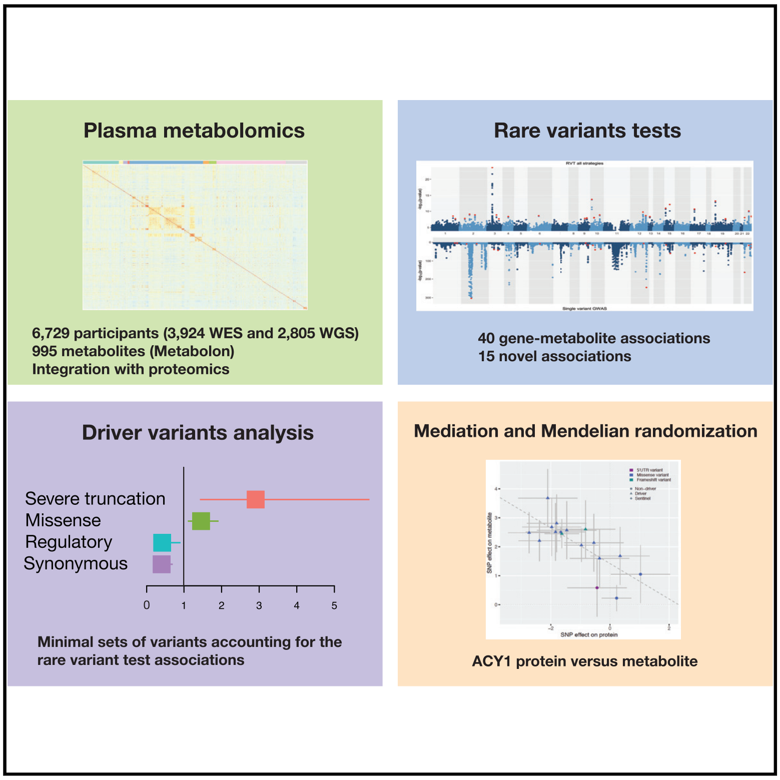

Authors

Lorenzo Bomba, Klaudia Walter, Qi Guo, ..., Ian Dunham, Adam S. Butterworth, Nicole Soranzo

Correspondence

ns6@sanger.ac.uk

Bomba et al., 2022, The American Journal of Human Genetics *109*, 1038–1054
June 2, 2022 © 2022 The Authors.
https://doi.org/10.1016/j.ajhg.2022.04.009

ARTICLE

Whole-exome sequencing identifies rare genetic variants associated with human plasma metabolites

Lorenzo Bomba,[1,2] Klaudia Walter,[1] Qi Guo,[3,8] Praveen Surendran,[3,4] Kousik Kundu,[1,4] Suraj Nongmaithem,[1] Mohd Anisul Karim,[1,2] Isobel D. Stewart,[5] Claudia Langenberg,[5,6] John Danesh,[1,3,7,8,9] Emanuele Di Angelantonio,[3,7,8,9,10] David J. Roberts,[8,11,12] Willem H. Ouwehand,[1,4] INTERVAL study, Ian Dunham,[1,2,13] Adam S. Butterworth,[3,7,8,9] and Nicole Soranzo[1,2,4,7,8,10,*]

Summary

Metabolite levels measured in the human population are endophenotypes for biological processes. We combined sequencing data for 3,924 (whole-exome sequencing, WES, discovery) and 2,805 (whole-genome sequencing, WGS, replication) donors from a prospective cohort of blood donors in England. We used multiple approaches to select and aggregate rare genetic variants (minor allele frequency [MAF] < 0.1%) in protein-coding regions and tested their associations with 995 metabolites measured in plasma by using ultra-high-performance liquid chromatography–tandem mass spectrometry. We identified 40 novel associations implicating rare coding variants (27 genes and 38 metabolites), of which 28 (15 genes and 28 metabolites) were replicated. We developed algorithms to prioritize putative driver variants at each locus and used mediation and Mendelian randomization analyses to test directionality at associations of metabolite and protein levels at the *ACY1* locus. Overall, 66% of reported associations implicate gene targets of approved drugs or bioactive drug-like compounds, contributing to drug targets' validating efforts.

Introduction

Variability of metabolite levels in the human population is influenced by both extrinsic and intrinsic factors. Genetic variation can affect metabolite levels by regulating the expression of enzyme-coding genes, modifying the structure of the enzyme or completely inactivating the enzyme in the case of protein-truncating variants. This could lead to a disruption of a particular metabolic pathway and, depending on the severity of this disruption, to the development of disease.[1] Metabolites are intermediate phenotypes between genes and clinical outcomes, and thus studying metabolites can aid the interpretation of effector genes of genome-wide association studies (GWASs) of complex traits and diseases. Genetic variants associated with metabolites are enriched near genes of pharmacological interest, aiding the evaluation of potential drug targets.[2] Often, genes involved in inborn errors of metabolism also harbor genetic variants associated with metabolite levels related to the disorder, and those same genetic variants may also be associated with complex traits and diseases.[2]

GWASs have identified hundreds of common (minor allele frequency [MAF] > 0.1%) genetic variants associated with metabolite levels.[2,3] Relatively less is known about the contribution to metabolites across the rare spectrum of genetic variation.[4–6] Studying variants with a predicted severe impact on proxies of protein function can inform drug discovery efforts[7] by aiding the interpretation of the phenotypic consequences of partial or complete gene "knockouts" in humans. A genetic variant that inactivates a gene encoding a drug target may mimic the pharmacological modulation of a drug, providing an "experiment of nature" to inform drug development. Metabolite levels associated with such variants could be used as readouts to infer clinical and therapeutic effects of a drug.

To expand our knowledge of rare high-impact (i.e., predicted loss-of-function and missense) variants associated with levels of nearly 1,000 plasma metabolites, we interrogated a cohort of apparently healthy blood donors recruited in the INTERVAL study[8] with whole-exome sequencing (WES) or whole-genome sequencing (WGS). We used a robust approach to aggregate loss-of-function and/or missense rare variants in test windows to improve the statistical power to detect signals. For comparison, we also explored synonymous variants with no predicted functional impact. Moreover, we developed a novel

[1]Wellcome Sanger Institute, Wellcome Genome Campus, Hinxton CB10 1SA, UK; [2]Open Targets, Wellcome Genome Campus, Hinxton CB10 1SD, UK; [3]British Heart Foundation Cardiovascular Epidemiology Unit, Department of Public Health and Primary Care, University of Cambridge, Cambridge CB1 8RN, UK; [4]Department of Haematology, University of Cambridge, Cambridge Biomedical Campus, Puddicombe Way, Cambridge CB2 0AW, UK; [5]MRC Epidemiology Unit, Institute of Metabolic Science, University of Cambridge, Cambridge CB2 0SL, UK; [6]Computational Medicine, Berlin Institute of Health at Charité – Utniversitätsmedizin Berlin, Berlin 10117, Germany; [7]British Heart Foundation Centre of Research Excellence, University of Cambridge, Cambridge CB2 0QQ, UK; [8]National Institute for Health Research Blood and Transplant Research Unit in Donor Health and Genomics, University of Cambridge, Cambridge CB1 8RN, UK; [9]Health Data Research UK Cambridge, Wellcome Genome Campus and University of Cambridge, Cambridge CB10 1SA, UK; [10]Human Technopole, Palazzo Italia, Viale Rita Levi-Montalcini 1, 20157 Milan, Italy; [11]NHS Blood and Transplant-Oxford Centre, Level 2, John Radcliffe Hospital, Oxford OX3 9BQ, UK; [12]Radcliffe Department of Medicine, University of Oxford, John Radcliffe Hospital, Oxford OX3 9BQ, UK; [13]European Molecular Biology Laboratory, European Bioinformatics Institute, Wellcome Genome Campus, Hinxton CB10 1SD, UK
*Correspondence: ns6@sanger.ac.uk
https://doi.org/10.1016/j.ajhg.2022.04.009.
© 2022 The Authors. This is an open access article under the CC BY license (http://creativecommons.org/licenses/by/4.0/).

method to assess the contributions of individual rare variants to the aggregated association signals. We also investigated whether any of the genes found to be associated with metabolite levels were also associated with protein levels measured in the same cohort. Overall, this study demonstrates the value of a densely phenotyped cohort for dissecting changes associated with metabolite and protein levels.

Subjects and methods

Study description

The INTERVAL study[8] comprises approximately 45,000 apparently healthy blood donors nested within a randomized trial of blood donation intervals. The trial has received ethics committee approval from the National Research Ethics Service Committee East of England - Cambridge East (Research Ethics Committee [REC] reference 11/EE/0538). Between mid-2012 and mid-2014, whole-blood donors aged 18 years and older were consented and recruited at 25 centers of England's National Health Service Blood and Transplant. All participants completed an online questionnaire including questions about demographic characteristics (e.g., age, sex, ethnic group), anthropometry (height, weight), lifestyle (e.g., alcohol and tobacco consumption), and diet. Participants were generally in good health because blood donation criteria exclude people with a history of major disease (such as myocardial infarction, stroke, cancer, HIV, and hepatitis B or C) and those who have had recent illness or infection. Study participants were randomly selected into two non-overlapping sub-cohorts of 4,502 and 3,762 participants, which were both screened with the Metabolon platform. The former sub-cohort was whole-exome sequenced, while the latter was whole-genome sequenced. Both were genotyped with the UK Biobank Axiom Array and imputed with a combined UK10K-1000G Phase III imputation panel.

Metabolite measurements

The non-targeted metabolomics analysis was performed at Metabolon (Durham, North Carolina, USA) on a platform consisting of four independent ultra-high-performance liquid chromatography–tandem mass spectrometry (UPLC–MS/MS) instruments.

Raw data were extracted, peaks identified, and quality control (QC) processed via Metabolon's hardware and software. Compounds were identified by comparison to library entries of purified standards or recurrent unknown entities. Metabolon maintains a library based on authenticated standards that contains the retention time/index (RI), mass-to-charge ratio (m/z), and chromatographic data (including MS/MS spectral data) on all molecules present in the library. Furthermore, biochemical identifications are based on three criteria: retention index within a narrow RI window of the proposed identification, accurate mass match to the library ± 10 ppm, and MS/MS forward and reverse scores between the experimental data and authentic standards. MS/MS scores are based on a comparison of the ions present in the experimental spectrum to the ions present in the library spectrum. While there may be similarities between these molecules based on one of these factors, the use of all three data points can distinguish and differentiate biochemicals. More than 3,300 commercially available purified standard compounds have been acquired and registered into LIMS for analysis on all platforms for determination of their analytical characteristics. Additional mass spectral entries have been created for structurally unnamed biochemicals, which have been identified by virtue of their recurrent nature (both chromatographic and mass spectral). These compounds have the potential to be identified by future acquisition of a matching purified standard or by classical structural analysis.

A variety of curation procedures were carried out to ensure that a high-quality dataset was made available for statistical analysis and data interpretation. The quality control and curation processes were designed to ensure accurate and consistent identification of true chemical entities and to remove those representing system artifacts, misassignments, and background noise. Metabolon data analysts used proprietary visualization and interpretation software to confirm the consistency of peak identification among the various samples. Library matches for each compound were checked for each sample and corrected if necessary.

Quality control of metabolites

Plasma samples from 8,536 INTERVAL participants that passed WES QC (Table S1) were sent to Metabolon for metabolite profiling. Plasma samples were sent in two batches and were thus processed at different times. Because of the potential for batch effects, QC of the metabolite data was done by batch. Metabolites with >100 missing values were excluded, and in total, 995 metabolites were available after QC. Metabolites were log-transformed by taking the natural logarithm. A metabolite value was defined as an outlier and winsorized where the value was 5 or more standard deviations away from the mean metabolite value. A principal-component analysis (PCA) was done with nonlinear iterative partial least squares because of the sparsity of the dataset. To help identify any multivariate outliers, we performed biplots comparing the first principal component to each of the next nine principal components. We undertook linear regressions of the first five principal components against age, sex, BMI, current smoking, alcohol consumption frequency, center, batch, plate (as a proxy for run day), appointment month (proxy for possible seasonal effects), and time between appointments (when blood samples were taken) and processing (measured in two ways because of varying levels of missing data: as hours and by days) to determine whether these factors were significantly associated with variability of the metabolites. As shown in Table S2, age, sex, BMI, current smoking, alcohol, INTERVAL center, plate, appointment month, and lag time between appointment and processing account for some of the variability in the metabolites. Adjusting the metabolite levels for some of these variables may be appropriate, however as a result of high levels of missingness, not all variables were considered. For this reason, log-transformed and winsorized metabolite values were adjusted in a linear regression only for age, sex, BMI, center, batch, plate, appointment month, time between appointment and processing, and the first five principal components of ancestry from multidimensional scaling. The metabolite residuals from this linear regression were then rank-inverse normalized and used as phenotype for association testing. To give a more detailed description of the covariates: age was calculated as the participant's age (in years) at the time at which the blood sample was collected. BMI was estimated as self-reported weight (in kilograms) divided by the square of self-reported height (in meters). Current smoking was assigned on the basis of the information provided by the participant about their tobacco smoking status at baseline, with adjustment for "current smoker" versus "never" or "former" smoker combined. More

specifically, current smoking was based on participants who responded "yes" to the question "Do you currently smoke?" while never/former smoking status was defined as those who responded "no" to that question and responded either "yes" (former) or "no" (never) to the question "Have you ever smoked?" Alcohol consumption frequency was also collected by self-report in the baseline questionnaire. Appointment month was taken as the month of the year in which the blood sample was collected and was used as a 12-factor categorical variable to account for potential seasonal effects. Finally, we also adjusted for the length of time between the blood collection (as proxied by the time of the appointment at the recruitment center) and the time at which the blood sample was fractionated at the processing laboratory after it had been shipped from the recruitment center. For the majority of samples (>95%), the processing happened the next day, so we used a binary variable (1 day versus >1 day) to account for those samples for which samples were not processed the following day. To account for more subtle effects, we also adjusted for the number of hours between the sample collection and sample processing as a continuous variable.

A total of 230 metabolic biomarkers were produced by the serum nuclear magnetic resonance (NMR) metabolomics platform (Nightingale Health)[9] on 46,097 samples in the INTERVAL cohort. Glucose, lactose, pyruvate, and acetate were excluded initially because of unreliable measurements. Conjugated linoleic acid and conjugated linoleic acid to total fatty acid ratio were set to missing for 3,585 samples showing signs of peroxidation. Creatinine levels were set to missing for 1,993 samples with isopropyl alcohol signals. Glutamine levels were set to missing for 347 samples that showed signs of glutamine to glutamate degradation. Samples with more than 30% missingness or identified as EDTA plasma were removed.

Protein measurements and quality control

We used a multiplexed, aptamer-based approach (SOMAscan assay) to measure the relative concentrations of 3,622 plasma proteins or protein complexes assayed via 4,034 modified aptamers in plasma. The proteins cover a wide range of molecular functions. Details of the protein measurements have been described previously.[10] After quality control and excluding samples with missing protein measurements, 3,301 participants overlapping with WES data remained for analysis.

Sequencing and quality control

WES and WGS were performed at the Wellcome Sanger Institute (WSI) sequencing facility.

For WES, sheared DNA was prepared for Illumina paired-end sequencing and enriched for target regions with Agilent's SureSelect Human All Exon V5 capture technology (Agilent Technologies; Santa Clara, California, USA). The exome-captured library preparation was sequenced with the Illumina HiSeq platform as paired-end 75 bp reads, reaching an average depth of approximately 50×. Reads were aligned to the GRCh37 human reference genome wiyh BWA (v0.5.10).[11] GATK HaplotypeCaller v3.4 [12] was used for variant calling and recalibration. Samples were excluded on the basis of the following criteria: (1) withdrawn consent; (2) estimated contamination >3% according to the software VerifyBamID;[13] (3) sex inferred from genetic data different from sex supplied; (4) non-European samples after manual inspection of clustering in 1000G PCA and choosing cutoffs on the first two PCs; (5) heterozygosity outliers (samples +/− 3 SDs away from the mean number of heterozygous counts); (6) non-reference homozygosity outliers (samples +/− 3 SDs away from the mean number of non-reference homozygous counts); (7) outlier Ti/Tv ratio (transition to transversion ratio +/− 3 SDs away from the mean ratio); and (8) excess singletons (number of singleton variants >3 SDs from the cohort mean). After QC, 4,070 samples were kept in the final release. Genetic variants with MAF > 1% were excluded with the following thresholds: (1) variant quality score recalibration (VQSR): 99.90% tranche; (2) missi ngness > 3%; and (3) Hardy Weinberg Equilibrium (HWE) $p < 1 \times 10^{-5}$. Genetic variants with MAF ≤ 1% were excluded with the following thresholds: (1) VQSR: 99.90% tranche; (2) genotype quality (GQ): <20 for SNPs and <60 for Indels; (3) sequencing depth (DP) < 2; and (4) allelic balance (AB) > 15 and < 80 for heterozygous variants. After genotype-level QC (GQ, DP, AB), only variants with <3% missingness were kept. A total of 1,716,946 variants were kept in the final release. Out of the 4,070 samples passing the QC, metabolite data were available for 3,924.

For WGS, sheared DNA was prepared for Illumina paired-end sequencing. Sequencing was performed with the Illumina HiSeq X platform as paired-end 75 bp reads, reaching an average depth of 15×. Reads were aligned to the GRCh38 human reference genome with mostly BWA (v.0.7.12) although a subset of samples was aligned with v.0.7.13 or v.0.7.15. GATK HaplotypeCaller v3.5 was used for variant calling and recalibration. We extracted coordinates from the VCF files that mapped to regions targeted in the WES. We then used custom scripts to transform coordinates of variants to the GRCh37 human reference. We filtered out samples on the basis of the following criteria: (1) estimated contamination > 2% according to the software VerifyBamID; (2) non-reference discordance (NRD) with genotype data on the same samples >4%; (3) population outliers from PCA (PC1 > 0 and minimum PC2); (4) heterozygosity outliers (samples +/− 3 SDs away from the mean number of heterozygous counts); (5) number of third-degree relatives (proportion IBD [PI-HAT)] > 0.125) > 18; and (6) overlap with WES. After quality control, 3,670 WGS samples were kept. Out of the 3,670 samples passing the QC, metabolite data were available for 2,805.

All the genetic variants reported in the text and in the tables were lifted to GRCh38.

Single-variant association test

Single-variant association tests were performed for each variant (all QC'ed whole-exome sequence variants) via an additive genetic model for all 995 metabolites. The association tests were carried out with RAREMETALWORKER v4.14.[14] The analysis software returns the summary statistics for each variant and each specific metabolite and a covariance matrix that reports the pairwise LD of variants in 1 MB regions. These statistics were subsequently used in RAREMETAL to perform rare variant aggregation tests as described in the next section. Genomic control values ranged from 0.98 to 1.04, indicating no substantial inflation or deflation due to population stratification.

Rare-variant aggregation tests

We used a total of four different rare-variant tests (RVTs) to investigate the aggregated effect of multiple rare variants with MAF < 0.1% on each trait, exploring two types of allelic architecture: (1) we used burden family tests, such as burden test, Madsen and Browning (MB), and variable threshold (VT), to discover

signals where variants with the same direction and magnitude of effects were tested together. They mostly vary on how they use weighted and unweighted functions with a fixed or variable frequency threshold. (2) SKAT is a variance-component multiple regression test that retains power in settings where neutral variants or variants with opposite direction of effects could result in loss of power. For the rare variant analyses, we used RAREMETAL v4.14,[14] which allows us to perform RVT by using single-variant test statistics and their correlations.

One of the biggest challenges of rare variant aggregation is to define sets of variants that identify domains encoding for a biological function. Our selection included all rare variants within coding exons, splice sites, or UTR regions of known genes (52,912 genes on autosomes) based on GENCODE v24 lifted over to build 37, only some types of pseudogenes (IG_C, IG_J, IG_V, TR_J, TR_V) were removed. Overlapping exons (528,874 exons) were merged within each gene, resulting in 301,736 exonic regions. Windows were generated by keeping the exon structure intact as far as possible and allowing between ~5 and ~20 variants per window. If there were less than five variants within an exon or more than 20 variants per gene, then windows were created by combining neighboring exons so that the number of variants was similar between windows. More specifically, the algorithm procedure is as follows. (1) If the first or last exon has fewer than five variants, the variants in this exon are combined with the variants in the neighboring exon. (2) If there is still exactly one exon with fewer than five variants, it is combined with its neighbor. (3) If an exon has more than 20 variants, it is split into roughly equal numbers of variants (e.g., 21 variants will be split into 11 and 10 variants). (4) The overall number of variants is calculated for each gene and the number of windows needed. (5) Then the average number of variants per window is calculated as a target so that the variants can be split equally between windows. (6) The number of variants per window is computed iteratively by adding the number of variants for each exon, starting with the first exon. (7) The optimal distribution is the one where the number of variants per window has the minimum difference to the target number.

Three different strategies in selecting variants were used. (1) CODING tests of all rare exonic variants, splice sites, and variants residing in UTRs. In the CODING approach, we tested 23,864 genes in 52,024 windows with 15 variants per window on average and a minimum of five and a maximum of 30 variants. (2) MLOF tests of LoF and missense variants combined; In total we analyzed 20,835 genes in 32,534 windows with 14 variants per window on average, and at least 5 variants and maximal 28 variants per window. iii) LOF variant tests; In the LOF approach we tested only LoF variants within each window predicted as high confidence (HC) by LOFTEE. LOFTEE (loss-of-function transcript effect estimator) is a plugin to the Variant Effect Predictor (VEP) that considers all stop-gained, splice-disrupting, and frameshift variants and filters out many known false-positive modes, such as variants near the end of transcripts and in non-canonical splice sites, as described in the code documentation. In total, we analyzed 9,385 genes in 9,428 windows with three variants per window on average and minimum two and maximum 19 variants per window. The distribution of the number of variants per window in each method are reported in Figure S1.

Multiple correction testing was performed with false discovery rate (FDR). We included p values tested in all analysis approaches to calculate q values by using the core R package function "p.adjust," that implements the Benjamini and Hochberg (1995) FDR method.

The approach described above could lead to false negative signals if variants associated with a given trait are distributed across different windows. For this reason, we also tested associations by combining variants across entire genes. Overall, this gene-based approach tested up to 23,864 genes under the same variant selection models described above, resulting in a greater average number of variants per gene compared to the window-based approach (range 5–2,024 under the CODING scenario).

Conditional analysis

We tested whether our RVT signals were independent from sentinel variants identified in the metabolite genome-wide association study (mGWAS) meta-analysis of INTERVAL and EPIC-Norfolk (P.S. and I.S.D., unpublished data). The sentinel variants were selected 500 kb upstream or downstream of the window of interest. To test for conditional independence, we included genotypes of the sentinel variants in the RVT as covariates. We calculated the difference of −log10(p values) before and after conditional analysis and we called it "delta". We arbitrarily set a delta threshold of 1 and called all the RVT signals having a delta value below the threshold independent from sentinel variants.

Forward selection procedure

We developed a forward selection procedure to identify a minimum set of variants that could explain the association in each test unit. This procedure is as follows. In the first step each variant is dropped one at a time and the test statistics recalculated. The new test statistics could either result in an increased or roughly unchanged p, which means that either the variant contributed considerably to the association p or not much. We define this p difference as "delta," and to verify the cumulative effect of the variants with high impact, we rank all the variants by the magnitude of delta. Finally, we apply a forward selection procedure, calculating the test statistics by adding each of the ranked variants until we reach the lowest test p. The set of variants that are necessary to achieve the lowest p are called "driver" variants because they are driving the association identified with the full test unit. We found that associations detected by the burden test family were driven by many contributing variants while associations detected by SKAT had only few driver variants. In those cases where deltas are quite similar, the final set of variants might be interchangeable.

Mediation and Mendelian randomization analysis

We used a traditional approach to mediation analysis consisting of comparing two regression models, one with and one without conditioning on the mediator. We used protein levels and metabolite levels, each as mediator, in two analyses to assess the direction of the associations between the genetic signals, protein levels, and metabolite levels.

If the dependent variable is regressed on the mediator and the genetic variants, and the size of the effect of the genetic signal remains similar, then this indicates that the mediator is unlikely to be on the causal path from the genetic variants to the dependent variable and is therefore not a mediator. However, when the effect of the genetic signal is much reduced compared to a regression model with the genetic variants as sole covariates, then this demonstrates a mediating effect on the path from the genetic variants to the dependent variable.[15]

After the mediation analysis, which established that metabolite level acts as mediator for protein level, we also carried out a two-stage least squares Mendelian randomization (MR) analysis, which

uses genetic variants as the instrumental variables, metabolite level as the exposure, and protein level as the outcome variable.[16] However, one has to keep in mind that an MR makes (among others) the assumptions that the instrument is independent of confounders and that the outcome is independent of the instrument conditioned on exposure and confounders. The latter assumption, for example, might not be fulfilled when protein level is taken as the exposure and metabolite level as the outcome variable because metabolite level is not conditionally independent of the genetic variants given protein level as seen in the above mediation analysis.

External data sources

We searched for the rare variants identified through our analysis in the UK Biobank (UKB) summary statistics by using two sources available: (1) Global Biobank Engine (GBE) with meta-analysis of array data, including White British, European, African, South Asian, East Asian, admixed, and related,[17] and (2) pheWEB - UKB pheWAS imputed with TOPMed.[18]

Annotation of missense variants

We explored the deleteriousness of missense variants with a number of functional prediction scores derived from the variant effect predictor (VEP v.85): sorting intolerant from tolerant (SIFT),[19] polymorphism phenotyping (PolyPhen),[20] combined annotation-dependent depletion (CADD),[21] and rare exome variant ensemble learner (REVEL).[21,22]

Results

Dataset and study design

We analyzed 995 metabolites measured with a non-targeted Metabolon HD4 metabolomics platform in plasma samples from 3,924 apparently healthy European-ancestry participants recruited in the INTERVAL study[23] (see subjects and methods and Table S1). 672 metabolites (68%) were chemically identified and assigned to eight biochemical super-pathways (i.e., amino acids, carbohydrates, cofactors and vitamins, energy, lipids, nucleotides, peptides, and xenobiotics). These broad categories can be further subdivided into 79 biochemical pathways (Table S3). The remaining 323 (32%) metabolites were of unknown chemical structure. To identify rare (MAF $< 0.1\%$) coding variants associated with metabolite levels, we accessed whole-exome sequencing (WES) of 3,924 participants (mean sequencing depth of 50×; subjects and methods), resulting in 1.72 million variants after strict QC.[24,25] To test associations with metabolites, we applied three variant selection strategies and two classes of statistical models in order to capture a broad spectrum of possible allelic architectures. As detailed in the methods, our primary analysis was based on the definition of windows of <20 variants each, but results were compared to a whole-gene-based analysis. For all scenarios, we applied three variant selection strategies: (1) variants predicted by LOFTEE to be loss-of-function with high confidence (LOF; i.e., essential splice site changes, stop codon gain, or frameshifts);[26] (2) missense + loss-of-function (MLOF); and (3) all coding region variants plus untranslated regions (UTRs) and essential splice sites (CODING). To achieve a comparable number of variants in each test region, we split genes into test windows of up to 20 variants on average while preserving intron-exon boundaries (GENCODE v24, Figure 1, Figure S1, Table S4, subjects and methods). For each test, we applied (1) three different implementations of burden tests (burden [BU],[27,28] Madsen and Browning [MB],[27,28] and variable threshold [VT][29]), which capture associations driven by variants with similar direction and magnitude of effect (see subjects and methods) and (2) a regression-based test, i.e., the sequence kernel association test [SKAT][30] to capture regions that include variants with opposite direction of effects, testing for both protective and risk alleles. We applied a p values cutoff ($p = 2.28 \times 10^{-8}$) to declare genome-wide significance, corresponding to a 5% global FDR (gFDR) value correcting for all models and phenotypes tested. We tested associations surpassing this significance threshold in an independent replication sample of 2,805 whole-genome sequences from non-overlapping INTERVAL participants by applying identical test window boundaries (mean sequencing depth of 15×, subjects and methods).[27,28]

Rare coding variants in 27 genes are associated with metabolite levels

Overall, using the window-based approach, our RVTs identified 40 signals in 27 genes associated with 38 metabolites, of which LOF, MLOF, and CODING tests identified 4, 33, and 17 gene-metabolite associations, respectively (gFDR $\leq 5\%$; Table 1, Table S5, Figure 2). We compared our results to published metabolite GWASs based on common or low-frequency variants captured by SNP array technology (mGWAS)[2,31,32] or rare variants reported in genome sequencing studies.[4,5,33] Of the 27 genes with an association in this study, two (*UMPS* and *SLC5A10*) had reported association with rare variants in a WGS-based study,[5] ten had associations involving common variants in SNP-based studies, and 15 identified new associations (described later). Overall, 4% of all metabolites were associated with at least one gene (mean 1.5 metabolites per gene, 1–7 range). Associations implicated rare genetic variants (median variant frequency = 0.013%, 148 singletons and $1 \geq$ allele count [AC] ≥ 7) compared to available signals from most recent mGWAS based on SNP array imputation. The rare variant associations included between five and 19 variants/window and had minor allele count (MAC) between one and seven. Variants had large effect sizes (e.g., 21% with beta > 2 SD standardized phenotype, range −3.67–3.68 SD). The whole-gene-based testing strategy discovered 37 additional associations, 2, 30, and 15 respectively for LOF, MLOF, and CODING, and 34 associations (21 genes) matched discoveries under the window-based model (Table S5).

We attempted to replicate the associations by using WGS data from 2,805 participants from the INTERVAL study not overlapping with the WES dataset. For 15 genes associated

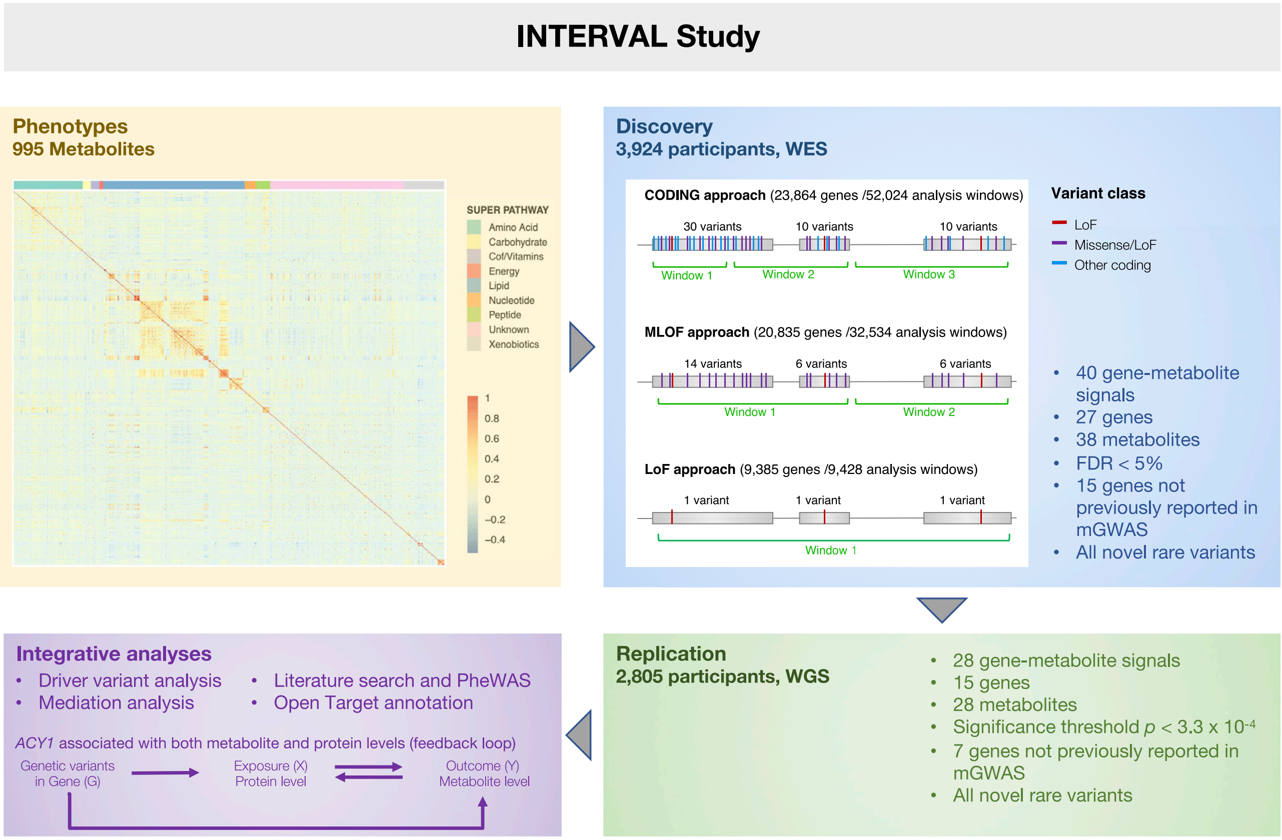

Figure 1. Study design, including data, methods, and results summary
Data —INTERVAL study description and correlation of metabolite levels ordered by super-pathways; rare variant test strategies —analysis windows (in green) were defined to be exons containing at most 20 rare variants (MAF $\leq$ 0.1%), three variant selection strategies were applied (CODING, MLOF, and LOF; variant classes are color coded), and for each strategy, four rare variant aggregation tests were used to explore different allelic architectures; results —results of WES RVT analysis at 5% FDR threshold and replication of discovery signals using WGS RVT analysis. Cof/Vitamins represent the super pathway cofactors and vitamins.

with 28 metabolites, we replicated associations between the reported window and the reported metabolite at a stringent Bonferroni correction ($p \leq 0.05/149 = 3.3 \times 10^{-4}$, Table S6), implicating ACY1, PTER, ADSL, NPL, KYNU, PAH, ACADS, LACTB, ABCC2, NAT8, CCBL1, UMPS, SLC5A10, ALB, and CERS4. The remaining associations did not reach this stringent level of significance because of absence of the corresponding rare variants from the WGS data.

We also attempted to replicate associations by using an independent metabolomic platform (Nightingale Health) based on NMR,[9] which includes 226 metabolites of different classes (ketone bodies, glycolysis related metabolites, amino acids, fluid balance, inflammation, fatty acids and saturation, cholesterol, glycerides and phospholipids, apolipoproteins, lipoprotein subclasses, and lipoprotein particle sizes). We tested the 27 genes by using models identical to the Metabolon-based discovery. Two of the relevant metabolites were shared between the metabolomics platforms, and associations were confirmed at two of the gene-metabolite associations, *PAH*-phenylalanine and *RGS3*-sphingomyelin ($p < 4.3 \times 10^{-5}$). Additionally, we scanned the phenotypes by searching for associations of the significant 27 genes with any of the 226 NMR metabolites, but we did not find any additional associations (Bonferroni $p < 8.19 \times 10^{-6}$) (Table S7).

Finally, we carried out conditional analyses to test whether the RVT associations may be explained by the presence of nearby common variants. We modeled the RVT associations while conditioning for nearby (<500 kb) common sentinel variants identified through a meta-analysis of INTERVAL and EPIC-Norfolk studies (P.S. and I.S.D., unpublished data) and found that most (93%) of the RV associations were independent from the proximal sentinel common variants, while *ACADS*-ethylmalonate and *NAT8*-N-acetyltyrosine narrowly missed the cutoff for independence (Table S8 and subjects and methods). We also used the WES data to search for novel metabolite associations with common coding genetic variants (MAF > 0.1%, n = 44,135) and we detected 1,836 signals in 580 genes at exome-wide significance level ($p < 2.63 \times 10^{-9}$) that had all been previously reported in mGWASs.

Biological, biochemical, and functional interpretation of associations

We next investigated the genetic architecture of each locus and their biochemical characteristics in detail. We assessed

Table 1. Associations of metabolites with 27 genes that are enriched for inborn errors of metabolism and drug targets

Gene symbol	Strongest metabolite	Super pathway	N driver variants	Variant selection strategy	RVT-WES p	RVT-WGS p	Replicated signal	mGWAS	OMIM disorder	Drug	Test
ABCC2	X - 21467	–	2	MLOF	1.5×10^{-9}	8.6×10^{-6}	yes	–	Dubin–Johnson syndrome (DJS)	bioactive compound	SKAT
ABCG5	campesterol	lipid	9	MLOF	1.6×10^{-8}	3.7×10^{-4}	no	–	sitosterolemia	bioactive compound	burden
ACADS	butyrylcarnitine	lipid	10	CODING	2.3×10^{-9}	1.2×10^{-7}	yes	yes	ACYL-CoA dehydrogenase, short-chain, deficiency of (ACADSD)	bioactive compound	burden
ACY1	N-acetylmethionine	amino acid	14	MLOF	2.1×10^{-24}	5.3×10^{-17}	yes	yes	aminoacylase 1 deficiency (ACY1D)	bioactive compound	variable threshold
ADSL	N6-succinyladenosine	nucleotide	9	MLOF	8.3×10^{-11}	2.8×10^{-13}	yes	–	adenylosuccinase deficiency (ADSLD)	N/A	burden
ALB	X - 22771	–	1	MLOF	4.5×10^{-9}	1.3×10^{-4}	yes	–	analbuminaemia (ANALBA), familial dysalbuminemic hyperthyroxinemia (FDAH)	bioactive compound	SKAT
CCBL1	indolelactate	amino acid	10	MLOF	1.2×10^{-8}	1.7×10^{-5}	yes	yes	–	bioactive compound	burden
CERS4	sphingomyelin (d18:1/20:1, d18:2/20:0)*	lipid	12	MLOF	6.2×10^{-14}	2.9×10^{-4}	yes	–	–	N/A	burden
CHKB	5-methyluridine (ribothymidine)	nucleotide	1	CODING	4.8×10^{-9}	7.2×10^{-1}	no	–	congenital Muscular dystrophy, megaconial type	bioactive compound	SKAT
CIC	1-(1-enyl-stearoyl)-2-linoleoyl-GPE (P-18:0/18:2)*	lipid	14	CODING	2.2×10^{-8}	9.5×10^{-1}	no	–	mental retardation, autosomal dominant 45 (MRD45)	N/A	burden
COMT	X - 11593	–	1	MLOF	9.2×10^{-9}	1.2×10^{-1}	no	yes	panic disorder 1 (PAND1), schizophrenia (SCZD)	approved drug	SKAT
CR1L	X - 21444	–	10	CODING	1.8×10^{-8}	9.5×10^{-1}	no	–	–	N/A	Madsen and Browning
DPCR1	2-aminobutyrate	amino acid	14	CODING	2.5×10^{-9}	3.5×10^{-1}	no	–	–	N/A	burden
ERICH6	glycerophosphorylcholine (GPC)	lipid	4	LOF	1.9×10^{-8}	2.3×10^{-1}	no	–	–	N/A	variable threshold
IVD	isovalerylcarnitine	amino acid	7	MLOF	1.1×10^{-11}	3.5×10^{-3}	no	yes	isovaleric acidemia (IVA)	bioactive compound	burden
KYNU	xanthurenate	amino acid	8	MLOF	1.4×10^{-9}	4.3×10^{-9}	yes	–	hydroxykynureninuria; Vertebral, cardiac, renal and limb defects syndrome 2 (VCRL2)	bioactive compound	burden

(*Continued on next page*)

Table 1. *Continued*

Gene symbol	Strongest metabolite	Super pathway	N driver variants	Variant selection strategy	RVT-WES p	RVT-WGS p	Replicated signal	mGWAS	OMIM disorder	Drug	Test
LACTB	succinylcarnitine	energy	9	MLOF	7.6×10^{-13}	5.5×10^{-6}	yes	yes	–	bioactive compound	variable threshold
NAT8	N-acetylarginine	amino acid	8	MLOF	8.4×10^{-10}	1.3×10^{-5}	yes	yes	–	N/A	burden
NPL	N-acetylneuraminate	carbohydrate	10	MLOF	3.3×10^{-9}	1.5×10^{-9}	yes	–	–	N/A	burden
PAH	phenylalanine	amino acid	9	MLOF	1.7×10^{-10}	5.2×10^{-8}	yes	yes	phenylketonuria (PKU) and hyperphenylalaninemia, non-PKU	approved drug	Madsen and Browning
PTER	N-acetyl-beta-alanine	nucleotide	11	MLOF	1.9×10^{-14}	3.3×10^{-8}	yes	–	–	N/A	Madsen and Browning
RGS3	stearoyl sphingomyelin (d18:1/18:0)	lipid	8	MLOF	1.6×10^{-8}	3.1×10^{-1}	no	–	–	N/A	variable threshold
SLC16A9	carnitine	lipid	9	MLOF	9.5×10^{-9}	4.3×10^{-1}	no	yes	–	N/A	variable threshold
SLC25A15	X - 15728	–	12	CODING	4.8×10^{-9}	5.3×10^{-1}	no	–	hyperornithemia-hyperammonemia-homocitrullonuria syndrome	bioactive compound	burden
SLC5A10	1,5-anhydroglucitol (1,5-AG)	carbohydrate	6	LOF	2.0×10^{-11}	5.4×10^{-5}	yes	rare	–	N/A	burden
TYMP	5-methyluridine (ribothymidine)	nucleotide	1	CODING	3.5 × 10-9	6.9×10^{-3}	no	yes	mitochondrial DNA depletion syndrome-1 (MTDPS1)	approved drug	SKAT
UMPS	Orotate	nucleotide	1	MLOF	1.4×10^{-9}	2.4×10^{-5}	yes	rare	orotic aciduria	bioactive compound	SKAT

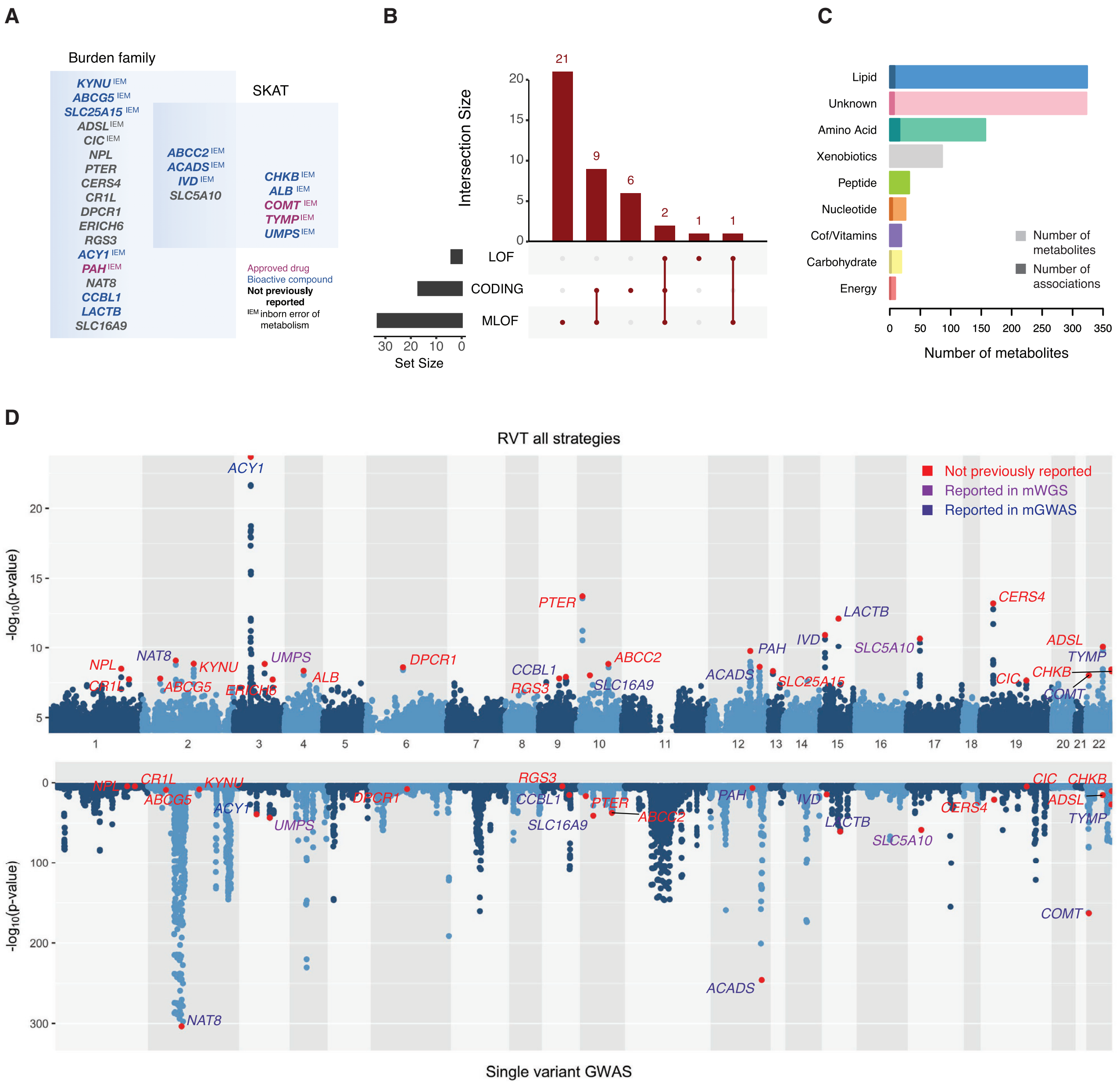

Figure 2. WES association results

(A) List of genes discovered by type of test (burden family and/or SKAT).

(B) UpSet plot of associations by approach. On the left, bar plot of total number of associations by approach and on top, bar plot of number of shared associations in multiple approaches. The number of associations in each set appears above the column, while approaches shared are indicated in the graphic below the column.

(C) Bar plot of all metabolites used in the analysis split by pathway and number of associated metabolites shown in darker color.

(D) Mirrored Manhattan plot showing −log10 Ps for WES single-variant tests (bottom) and WES rare-variant tests (top). Strongest gene-metabolite associations are highlighted in red. All genetic associations derived from any approach or aggregation test are reported in the RVT Manhattan plot. All 27 genes found to be associated with metabolites in RVT are labeled in the plot. Gene label color code highlights genes as not previously reported (red), reported in mWGS (purple), or reported in mGWAS (blue).

the proteins and diseases that are reportedly linked to each gene-metabolite pair, employing a range of bioinformatic tools and data repositories.[34,35] Overall, the majority of associations had a clear biochemical rationale and could plausibly be explained by protein-coding variants altering the efficiency of the enzyme reactions (Table S9). To inform the genetic structure of each locus, we used two alternative approaches based on either leave-one-out/forward selection or Lasso (subjects and methods) to identify minimal sets of variants with the greatest probability of accounting for the RVT association signal ("driver" variants, which are not necessarily all causal variants).

Using the forward selection approach, we found that the majority of SKAT signals could be accounted for by only one or two driver variants, whereas the number of driver variants in burden tests was greater (4–14). Overall, the

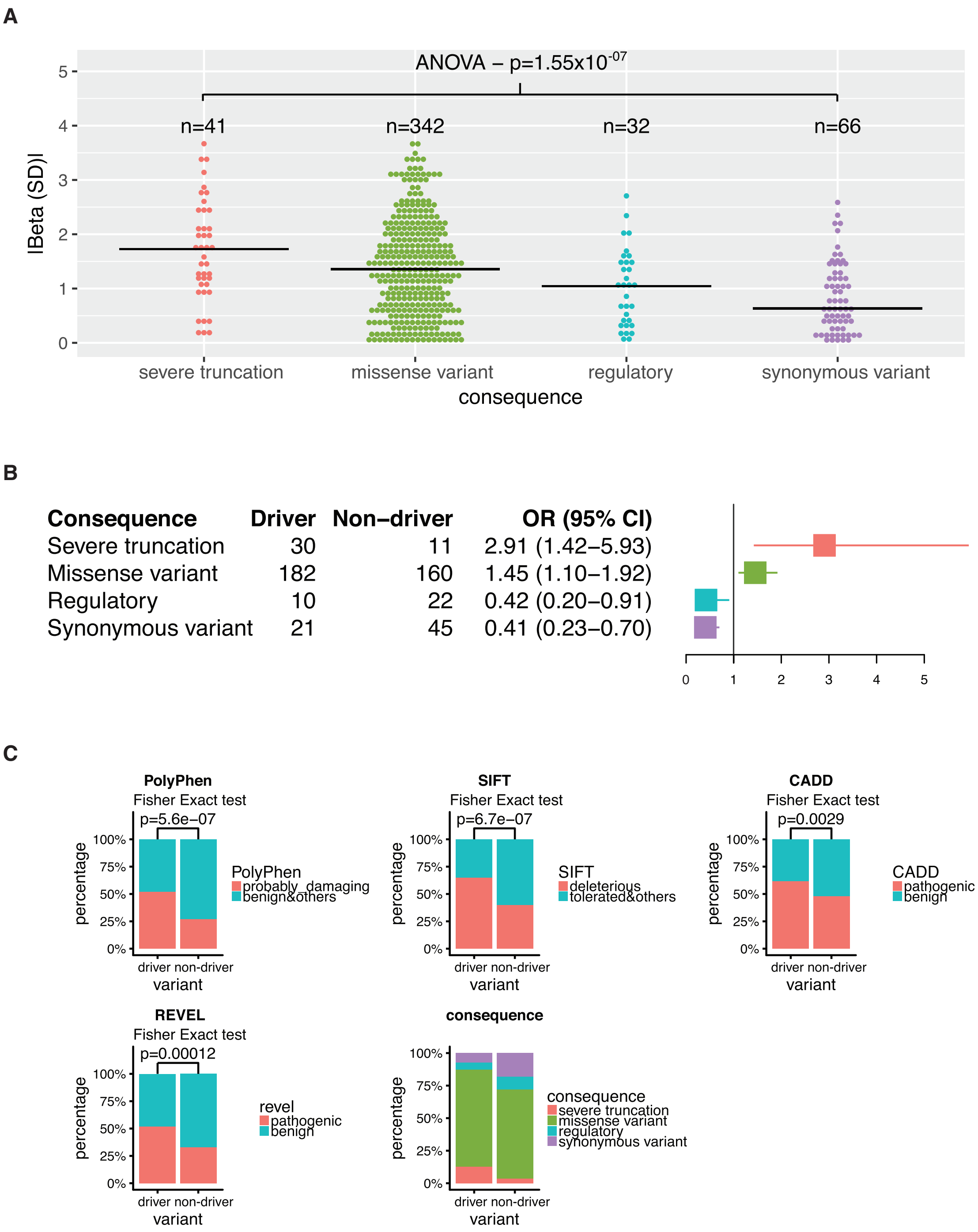

Figure 3. Driver variants analyses
(A) Absolute effect size (beta in SD) of driver variants split by their predicted consequence on protein.
(B) Enrichment of driver variants split by their predicted consequence on protein.
(C) Enrichment of driver variants by different functional prediction methods: Polyphen, SIFT, CADD, REVEL, and predicted consequences.

proportion of driver variants identified by Lasso and forward selection was 55%, and 66% if SKAT tests were excluded from the comparison (Figure S2). We found that driver variants predicted to cause severe protein truncation tended to have higher effect sizes when compared to other functional classes (Figure 3). Driver variants were enriched for protein-truncating variants (i.e., splice donor, splice acceptor, frameshift, and stop gained variants) with 3-fold increased odds. However, missense was the most represented category among all rare variants and we show that missense driver variants are significantly enriched for being likely deleterious (Figure 3 and subjects and methods). For 12 genes, we were able to confirm for each variant the biological consistency of the direction of

effect between gene and metabolite. This means that in some cases the substrate metabolites accumulate if missense or LoF variants are decreasing the efficiency of the enzyme or transporter, while in other cases disrupted gene function results in reduced levels of the metabolite product (Table S5). In the following sections we focus on results generated from the window-based approach.

Neurological function

We observed associations of 18 rare variants in *ACY1* (14 drivers) with seven metabolites, including N-acetyl methionine (VT test, $p_{WES} = 2.1 \times 10^{-24}$, $p_{WGS} = 5.3 \times 10^{-17}$), five N-terminal acetylated amino acids (N-formylmethionine, N-acetylserine, N-acetylglutamate, N-acetylthreonine, N-acetylvaline, and N-acetylalanine), and N-formylmethionine (Table S10). *ACY1* encodes aminoacylase-1, a homodimeric zinc-binding metalloenzyme involved in the hydrolysis of N-acetylated proteins. The two most significant encoded non-synonymous changes are predicted to disrupt protein function: frameshift p.Ser192fs (rs770702363, MAF = 0.051%, beta(SE) = 2.449(0.4997), $p = 9.45 \times 10^{-7}$) and missense p.Asp174Gly (rs200314495, MAF = 0.013%, beta(SE) = 3.679(0.999), $p = 2.30 \times 10^{-4}$, SIFT = deleterious, PolyPhen = probably damaging). rs770702363 is predicted to be pathogenic and located within the M20 peptidase domain, in the proximity of metal ion binding (protein position: 175). The remaining driver variants were all missense changes, with the exception of another frameshift variant 3:51987362:C:CT (Table S5).

A possible interpretation of the association is that the combined effect of missense variants may reduce the overall enzymatic activity of aminoacylase 1, leading to an increase of their substrate (in this case N-acetylated amino acids). *ACY1* has been described in OMIM as a gene causing aminoacylase 1 deficiency (ACY1D), a rare inborn error of metabolism characterized by increased urinary excretion of specific N-acetyl amino acids, and most affected individuals show neurologic abnormalities such as intellectual disability, seizures, hypotonia, and motor delay.[36] We therefore searched two UKB PheWAS databases, GBE[17] and PheWeb,[18] for associations with complex neurological phenotypes (subjects and methods). Driver variant rs6804746 (MAF = 0.02%) was associated with ICD10 code G31 ("other degenerative diseases of the nervous system not elsewhere classified", GBE; $p = 4.6 \times 10^{-8}$) and with chronic fatigue syndrome (PheWeb; $p = 3.5 \times 10^{-4}$). Two other driver variants associated with mental disorders in PheWeb, namely rs1164299165 with pervasive developmental disorders (MAF = 0.005%; $p = 9.7 \times 10^{-4}$) and rs887540 with major depressive disorder (MAF = 0.03%; $p = 3 \times 10^{-4}$).

Complex carbohydrates and cellular infectivity

Rare variants in *NPL* were associated with increased N-acetylneuraminate (burden test, $p_{WES} = 3.2 \times 10^{-9}$, $p_{WGS} = 1.5 \times 10^{-9}$). *NPL* encodes a member of the N-acetylneuraminate lyase subfamily, which regulates cellular concentrations of N-acetylneuraminate by mediating the reversible aldol condensation between N-acetyl-d-mannosamine (ManNAc) and pyruvate to N-acetylneuraminate.

The signal included 15 variants (ten drivers), including a missense (rs141892236, MAF = 0.090%, beta(SE) = 1.552(0.378), $p = 4.13 \times 10^{-5}$) and a splice donor variant (rs757256606, MAF = 0.026%, beta(SE) = 2.005(0.707), $p = 4.62 \times 10^{-3}$) and several other missense changes close to the active protein sites at positions 143 and 173 (rs146355388, p.Pro146Thr, MAF = 0.039%, beta(SE) = 1.550(0.578), $p = 7.32 \times 10^{-3}$; rs148306247, p.Glu156Ala, MAF = 0.090%, beta(SE) = 0.654(0.378), $p = 8.43 \times 10^{-2}$; rs138338286, p.Glu157Val, MAF = 0.026%, beta(SE) = 0.685(0.707), $p = 3.33 \times 10^{-1}$). We can hypothesize that reduced enzymatic activity of NPL driven by rare variation may lead to the accumulation of N-acetylneuraminate, which is then recycled and returned to the cell surface, potentially increasing susceptibility to bacterial and viral pathogenicity. Interestingly, rs148306247, rs146355388, and rs138338286 were all associated with different infection-related traits in both GBE and PheWeb, reflecting the importance of the N-acetylneuraminate in host-parasite interactions. Finally, rs141892236 was associated with septicemia in GBE ($p = 2.3 \times 10^{-11}$), while a weaker signal for Streptococcus infection was found in PheWeb ($p = 3 \times 10^{-3}$), reinforcing the role of this gene in viral and bacterial infection.

Lipids homeostasis and phytosterols

We observed an association between variants in *ABCG5* and an increase in campesterol levels (burden test, $p_{WES} = 1.6 \times 10^{-8}$, $p_{WGS} = 3.7 \times 10^{-4}$). *ABCG5* encodes ATP-binding cassette subfamily G member 5 (ABCG5), an ABC transporter involved in the lipid homeostasis pathway transporting sterols from the cytosol to the extracellular domain, limiting intestinal absorption and promoting biliary excretion of sterols. Campesterol is a phytosterol (PS), or a steroid derived from plants. As a food additive, phytosterols have cholesterol-lowering properties (by reducing cholesterol absorption in intestines) and may act in cancer prevention. The signal included 14 variants (nine drivers), of which the two most significant associations encode for missense changes (rs755523464, p.Tyr487Cys, MAF = 0.025%, beta(SE) = 3.491(1.002), $p = 4.94 \times 10^{-4}$ and 2:43822798:A:G, p.Trp488Arg, MAF = 0.025%, beta(SE) = 3.185(1.002), $p = 1.48 \times 10^{-3}$) (Table S5).

We hypothesize that rare variants in *ABCG5* could reduce the efficiency in transporting sterols, therefore increasing plasma dietary campesterol. The missense variant rs150401285 (MAF = 0.1%) was associated with cholesterol ($p = 4.9 \times 10^{-14}$), LDL cholesterol ($p = 1.1 \times 10^{-13}$), and apolipoprotein B ($p = 3.9 \times 10^{-9}$) in GBE and with cholelithiasis and cholecystitis in PheWeb ($p = 4.6 \times 10^{-4}$), indicating that loss of *ABCG5* activity

due to rs150401285 results in a protective effect on gallstones. Interestingly, a recent GWAS of gallstone disease[37] showed that the missense p.Asp19His variant in *ABCG5/ABCG8* (gain-of-activity) increases the risk for gallstone disease through increases in biliary cholesterol secretion and decreases in dietary cholesterol intake in the gut. It remains to be determined whether this association is mediated by decreased cholesterol levels.

Kynurenine pathway

Variants in *KYNU* were associated with increased levels of xanthurenate, a metabolite from the tryptophan catabolism (burden test, $p_{WES} = 1.4 \times 10^{-9}$, $p_{WGS} = 4.3 \times 10^{-9}$). *KYNU* encodes for Kynureninase (EC 3.7.1.3), a 3-hydroxykynureninase-type enzyme involved in the kynurenine pathway for the biosynthesis of NAD cofactors from tryptophan. It catalyzes the conversion of L-3-hydroxykynurenine and L-kynurenine to 3-hydroxyant hranilic acid and anthranilic acid, respectively. The signal included 12 variants (eight drivers), of which two missense variants with the strongest association (rs137982021, MAF = 0.065%, beta(SE) = 2.172(0.447), $p = 1.20 \times 10^{-6}$ and 2:142985130:A:G, MAF = 0.013%, beta(SE) = 3.065(0.999), $p = 2.17 \times 10^{-3}$) encode changes predicted to impact the catalytic efficiency of Kynureninase, leading to an increase in the levels of L-3-hydroxykynurenine (substrate) that is converted to xanthurenate by kynurenine aminotransferase (KATs). The amino acid substitutions that we identified span protein positions 212 to 432, corresponding to the aminotransferase domain. Further, they are all located downstream of the reported homozygous mutation (encoding p.Thr198Ala) that causes hydroxykynureninuria, an inborn error of metabolism characterized by accumulation of kynurenine, 3-hydroxykynurenine, and xanthurenic acid excreted in the urine.[38] The absence of kynureninase results in a block in the pathway from tryptophan to nicotinic acid, and can result in niacin (vitamin B3) deficiency. The clinical phenotype has a wide range from asymptomatic to severe, characterized by intellectual disability, cerebellar ataxia, pellagra, progressive encephalopathy with muscular hypotonia, global developmental delay, stereotyped gestures, and/or congenital deafness.

CCBL1 is another signal that is part of the tryptophan pathway and was associated with increased levels of indolelactate (burden test, $p_{WES} = 1.2 \times 10^{-8}$, $p_{WGS} = 1.7 \times 10^{-5}$). *CCBL1* encodes for kynurenine aminotransferase (KYAT1; EC 2.6.1.7) and it is part of the tryptophan catabolism pathway that converts L-kynurenine and L-3-hydroxykynurenine into kynurenate and xanthurenate, respectively. Indolelactate is also part of the tryptophan catabolism pathway metabolized via a series of indoles. This process is mainly enabled by gut microbiota and in particular *Clostridia*. Though the direct biochemical mechanism of this association is still unclear, it is interesting to note that serum levels of indolelactate were found to be significantly lower in adults with multiple sclerosis, as well as the bacteria producing it.[39]

KYNU and CCBL1 are enzymes of the kynurenine pathway that have multiple biological implications, such as an active role in the immune response; some kynurenins are neuroactive and the kynurenine pathway is involved in many diseases such as Alzheimer's disease, amyotrophic lateral sclerosis, Huntington's disease, AIDS dementia complex, malaria, cancer, depression, and schizophrenia, where imbalances in tryptophan and kynurenines have been found.[40] Specifically, the missense variant rs137982021 (MAF = 0.03%) was associated with cholecystitis without cholelithiasis ($p = 2.6 \times 10^{-4}$), renal failure NOS ($p = 1.7 \times 10^{-3}$), and other specified cardiac dysrhythmias ($p = 1.8 \times 10^{-3}$) in PheWeb, confirming its implication in a broad range of disease categories.

Signals overlapping with drug targets

In total, 66% of the rare variant associations identified in our study were found within genes of pharmacological interest. Three genes (*COMT*, *TYMP*, and *PAH*) discovered in our study overlap drug targets for four approved drugs (entacapone, tolcapone, tipiracil, and sapropterin). In addition, 12 of the genes (*CHKB*, *UMPS*, *ALB*, *ABCC2*, *IVD*, *KYNU*, *LACTB*, *ABCG5*, *CCBL1*, *ACADS*, *SLC25A15*, and *ACY1*) are targets for bioactive drug-like compounds that were experimentally validated in ChEMBL. Entacapone and tolcapone are both inhibitors of catechol-O-methyltransferase (COMT), used in the treatment of Parkinson's disease as an adjunct to levodopa/carbidopa therapy. COMT eliminates biologically active catechols and other hydroxylated metabolites. In the presence of a decarboxylase inhibitor, COMT becomes the major metabolizing enzyme for levodopa, catalyzing the metabolism to 3-methoxy-4-hydroxy-L-phenylalanine (3-OMD) in the brain and periphery. The mechanism of action of entacapone is believed to be through its ability to inhibit COMT and alter the plasma pharmacokinetics of levodopa. The gene target and two inhibiting drugs are also associated with several other brain-related diseases and addictions, such as schizophrenia, cocaine dependence, gambling behavior (phase II), and epilepsy. Tipiracil is a small molecule first approved in 2015 and indicated for the treatment of adults with metastatic colorectal cancer. Tipiracil selectively inhibits thymidine phosphorylase (TYMP), a cytosolic enzyme essential for the nucleotide salvage pathway. Sapropterin was approved in 2007 and is today a well-established drug for the treatment of phenylketonuria. It is a small molecule targeting phenylalanine hydroxylase (PAH) to activate the hydroxylation of L-phenylalanine to L-tyrosine. Sapropterin has recently been associated with other indications in phase III and IV, including hyperphenylalaninemia and peripheral arterial disease.

Mediation and Mendelian randomization analysis via protein level

For a subset of the participants included in this study (n = 3,301), we also interrogated the plasma proteome, using an

expanded version of an aptamer-based multiplex protein assay (SOMAscan, Somalogic)[41] to quantify 3,622 plasma proteins.[10] We conducted RVT for all our metabolite-associated genes, searching for *cis* and *trans* associations with available protein levels by using the same analysis strategy as described previously (the same variants, the same grouping within windows, and the same selection strategy). We identified a significant ($p = 5.9 \times 10^{-9}$; beta = −1.09; MLOF approach) *cis* protein level association within *ACY1*—our strongest signal in the Metabolon analysis. The same rare genetic variants in *ACY1* were associated with metabolite and protein levels, implying highly concordant allelic architectures.

To investigate the predicted functional impact of rare *ACY1* variants, we performed a structural analysis of this protein by using UCSF Chimera[42] and evaluated the impact of substituting the different rotamers on the protein structure. The p.Gln26Pro substitution had a potential to create steric clashes in the proximity of the active site of the enzyme. To investigate the relationship between the protein and metabolite levels associated with *ACY1*, we performed a mediation analysis, testing two alternative models where N-acetylmethionine and ACY1 protein levels were fit as covariates in a model testing associations of rare variants with protein and N-acetylmethionine levels, respectively (Figure 4). The results were consistent with a scenario where N-acetylmethionine level may mediate associations of genetic variants to ACY1 protein levels.

Once we established that the metabolite level lies on the causal path from the genetic variants to protein level, we aimed to isolate the direct effect of metabolite level on protein level from potential confounders via Mendelian randomization (MR). Our MR analysis uses the genetic variants as the instrumental variable, metabolite level as the exposure, and protein level as the outcome variable. A two-stage analysis showed that the MR estimate for the effect of metabolite level on protein level (beta = −0.615, $p < 2 \times 10^{-16}$) is very similar to the estimate from a linear model (beta = −0.592, $p < 2 \times 10^{-16}$) without an instrument. This indicates that the relatively strong negative influence of metabolite levels on protein levels is genuine and unlikely to be the effect of confounding.

Discussion

In this study, we describe an association study of rare genetic variants with blood plasma concentrations of 995 metabolites in almost 4,000 apparently healthy blood donors. We identified 40 gene-metabolite associations in 27 genes and 38 metabolites by using a windows-based approach. Of these, only one association driven by a rare variant has been already described (rs200305064 with orotate);[5] for 11 additional genes, there was previous evidence for association at the same locus but driven by independent common genetic variants. Signals from 15 genes (seven new) were replicated with WGS data from the same study, while for the others, replication p values did not reach the predefined significance cutoff, most likely through a lack of the corresponding driver variants.

Our rare variant test strategy was designed to explore different allelic architectures through multiple approaches. Compared with the approach used by Long et al.,[5] we sought to increase statistical power by aggregating variants of different predicted functional effects within genomic windows defined by intron-exon boundaries. This allows us to detect associations where gene-wide associations could not be detected, for instance, in the case of the *ABCG5* gene associated with campesterol or *RGS3* with stearoyl sphingomyelin (d18:1/18:0). In the latter case, the third test window contained multiple functional domains more likely to harbor rare variants disrupting the sphingomyelin pathway. However, this strategy reduces power for cases where the contributing variants are spread across the gene, and indeed we found 37 associations that surpassed the significance threshold at gene level but not in the window scenario. Overall, the MLOF approach yielded a greater number of new discoveries and only one association was specific to the LOF approach. This most likely reflects an optimal number of functional variants included in the testing windows, for the current sample size, and underlying allelic architecture. As expected, multiple RVT signals were shared among different test types and especially between MLOF and CODING (eight genes in total). Most of the associations were identified by the aggregated contributions of many singletons and fewer variants with higher allele count. Interestingly, 21% of these variants presented with an effect size greater or equal to 2, demonstrating the power of WES to identify rare variants with large effect sizes within genes of pharmacological interest. Conditional analyses using nearby (<500 kb) common sentinel variants identified through mGWAS confirmed that RVT associations are mostly independent from proximal common sentinel variants.

Our algorithms to identify putative driver variants, i.e., variants that are more likely to contribute to the association signal, confirms different architectures underlying SKAT and burden signals, and SKAT tests are typically explained by small numbers of variants of greater allelic frequencies. Driver variants were enriched for variants of predicted functional impact, for instance causing a severe truncation, and missense variants predicted as deleterious by multiple approaches (Polyphen, SIFT, CADD, and REVEL), confirming the validity of this approach.

The new associations were enriched near genes causative for inborn error of metabolism (IEM) and genes associated in mGWASs of common variants. The allele frequencies and effects sizes of the new associations were intermediate between the two, confirming a continuum of genetic contributions to metabolic function mediated by the same genes. While as much as 55% of RVT associations were in IEM genes, only a handful were known pathological variants for recessive diseases (ClinVar or OMIM) for which

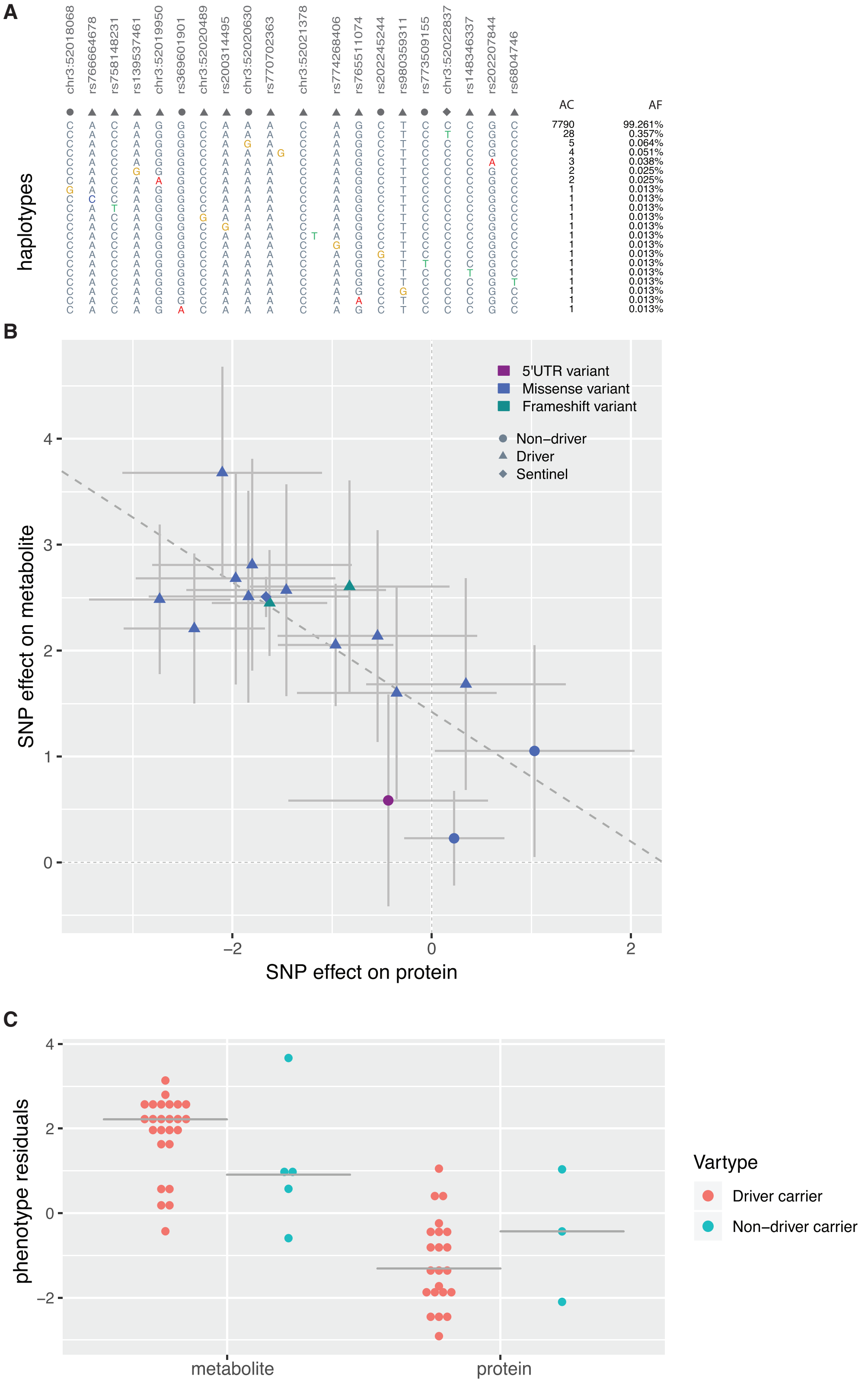

Figure 4. ***ACY1*/N-acetylmethionine in depth analysis**
(A) Proportions of haplotypes composed of non-driver, driver, and sentinel variants that are represented by their point shape in (B).
(B) Scatter plot of effect sizes and relative standard errors of shared protein versus metabolite associations; color coding indicates predicted consequence on protein and point shape indicates type of variants.
(C) Dot plots of phenotype residuals in carriers of driver and non-driver variants for both metabolite and protein levels.

no healthy homozygous carriers were present in our study participants.

For almost all of the gene-metabolite associations, we were able to identify the underlying biochemical function, where these functions implicated genes with important biomedical functions. For instance, *NPL*, which is associated with N-acetylneuraminate (sialic acid, NANA, Neu 5Ac) belongs to an ancient pathway conserved in bacteria. N-acetylneuraminate is an essential component of complex carbohydrates, which play pivotal roles in a variety of cellular recognition and communication processes including host-parasite interactions. Another example is the association between *ABCG5/G8* and phytosterols (PSs). Exogenous sterols (including PS) have been shown to have cholesterol-lowering properties. Reduction of up to 15% achieved in human subjects[43] may be mediated by competitive intestinal solubilization into mixed cholesterol/PS micelles, or increases in intestinal and hepatic-biliary secretion mediated by *ABCG5/G8* upregulation by PS. Consequently, several studies have reported correlations between phytosterol levels and cardiovascular health[44] mediated by common variants in *ABCG8* and *ABO*.

Our hypothesis-free approach revealed new hypotheses on the mechanisms through which associations with metabolites may act. We compared our associations with a dataset of proteins and found concomitant associations with protein levels at the *ACY1* locus. Interestingly, we found that variants associated with an accumulation of N-ace tylmethionine were also associated with a decrease of ACY1 protein levels. Through a mediation analysis, we infer a directional effect whereby protein levels are mediated by the accumulation of metabolite, which would suggest the existence of negative feedback of the metabolite onto the protein. A possible hypothesis is that rare LoF and missense variants, which are predicted in this study to cause steric clashes at the active site, may reduce the efficiency of the protein in clearing the substrate. The potential medical impact of this association extends beyond the neurological function described earlier. A recent study has described a strong positive correlation between ACY1 protein levels and type 2 diabetes (T2DM).[45] Through *in vitro* and *in vivo* experiments, the authors showed that increasing amounts of ACY1 decreased the ratio of N-acetyl/free amino acids, with a consequent effect on glucose and insulin homeostasis, possibly leading to β-cell exhaustion, reduced β-cell mass, and ultimately insulin deficiency and T2DM. In another recent study in a subset of participants from the INTERVAL study, a polygenic risk score for T2DM was associated with ACY1 protein levels, thus strengthening the link between *ACY1* and T2D risk.[46]

Overall, our findings illustrate the value of endophenotypes including metabolites and proteins to enhance our understanding of previously known genetic risk factors for disease. This is even more evident when, as in our case, sequencing data were used for identification of rare coding variants with large effect sizes associated with metabolite/protein levels. These studies generate new hypotheses to support therapeutic target identification and validation.

Data and code availability

Whole-exome sequencing data for the INTERVAL cohort is available in EGA: https://www.ebi.ac.uk/ega/datasets/EGAD00001002221. All of the summary statistics are available at Sanger ftp site: ftp://ftp.sanger.ac.uk/pub/project/humgen/summary_statistics/INTERVAL_WES_metabolon. All of the codes for this study are publicly available at GitHub: https://github.com/teamsoranzo/MetabolomicsWorkflow.

Supplemental information

Supplemental information can be found online at https://doi.org/10.1016/j.ajhg.2022.04.009.

Declaration of interests

John Danesh reports grants, personal fees, and non-financial support from Merck Sharp & Dohme (MSD); grants, personal fees, and non-financial support from Novartis; grants from Pfizer; and grants from AstraZeneca outside the submitted work. John Danesh sits on the International Cardiovascular and Metabolic Advisory Board for Novartis (since 2010); the Steering Committee of UK Biobank (since 2011); the MRC International Advisory Group (ING) member, London (since 2013); the MRC High Throughput Science 'Omics Panel Member, London (since 2013); the Scientific Advisory Committee for Sanofi (since 2013); the International Cardiovascular and Metabolism Research and Development Portfolio Committee for Novartis; and the AstraZeneca Genomics Advisory Board (2018). Adam Butterworth reports institutional grants from AstraZeneca, Bayer, Biogen, BioMarin, Bioverativ, Merk and Sanofi. During the course of the project Praveen Surendran became an employee of GSK, Lorenzo Bomba became an employee of BioMarin, Mohd Karim became an employee of Variant Bio and Qi Guo became an employee of BenevolentAI.

Received: August 19, 2021
Accepted: April 13, 2022
Published: May 13, 2022

Web resources

Clinvar, https://www.ncbi.nlm.nih.gov/clinvar/
Global Biobank Engine, https://biobankengine.stanford.edu/
HGMD, https://www.hgmd.cf.ac.uk/
Loftee, https://github.com/konradjk/loftee
Open Targets, https://platform.opentargets.org/
Orphanet, https://www.orpha.net/
UK Biobank TOPMed-imputed PheWeb, https://pheweb.org/UKB-TOPMed/
Vep, https://www.ensembl.org/info/docs/tools/vep/index.html

References

1. Kastenmüller, G., Raffler, J., Gieger, C., and Suhre, K. (2015). Genetics of human metabolism: an update. Hum. Mol. Genet. *24*, R93–R101.

2. Shin, S.-Y., Fauman, E.B., Petersen, A.-K., Krumsiek, J., Santos, R., Huang, J., Arnold, M., Erte, I., Forgetta, V., Yang, T.-P., et al. (2014). An atlas of genetic influences on human blood metabolites. Nat. Genet. *46*, 543–550.
3. Suhre, K., Shin, S.-Y., Petersen, A.-K., Mohney, R.P., Meredith, D., Wägele, B., Altmaier, E., CARDIoGRAM, Deloukas, P., Deloukas, J., Erdmann, J., et al. (2011). Human metabolic individuality in biomedical and pharmaceutical research. Nature *477*, 54–60.
4. Yousri, N.A., Fakhro, K.A., Robay, A., Rodriguez-Flores, J.L., Mohney, R.P., Zeriri, H., Odeh, T., Kader, S.A., Aldous, E.K., Thareja, G., et al. (2018). Whole-exome sequencing identifies common and rare variant metabolic QTLs in a Middle Eastern population. Nat. Commun. *9*, 333.
5. Long, T., Hicks, M., Yu, H.-C., Biggs, W.H., Kirkness, E.F., Menni, C., Zierer, J., Small, K.S., Mangino, M., Messier, H., et al. (2017). Whole-genome sequencing identifies common-to-rare variants associated with human blood metabolites. Nat. Genet. *49*, 568–578.
6. Feofanova, E.V., Yu, B., Metcalf, G.A., Liu, X., Muzny, D., Below, J.E., Wagenknecht, L.E., Gibbs, R.A., Morrison, A.C., and Boerwinkle, E. (2018). Sequence-based analysis of lipid-related metabolites in a multiethnic study. Genetics *209*, 607–616.
7. Finan, C., Gaulton, A., Kruger, F.A., Lumbers, R.T., Shah, T., Engmann, J., Galver, L., Kelley, R., Karlsson, A., Santos, R., et al. (2017). The druggable genome and support for target identification and validation in drug development. Sci. Transl. Med. *9*, eaag1166.
8. Moore, C., Sambrook, J., Walker, M., Tolkien, Z., Kaptoge, S., Allen, D., Mehenny, S., Mant, J., Di Angelantonio, E., Thompson, S.G., et al. (2014). The INTERVAL trial to determine whether intervals between blood donations can be safely and acceptably decreased to optimise blood supply: study protocol for a randomised controlled trial. Trials *15*, 363.
9. Riveros-Mckay, F., Oliver-Williams, C., Karthikeyan, S., Walter, K., Kundu, K., Ouwehand, W.H., Roberts, D., Di Angelantonio, E., Soranzo, N., Danesh, J., et al. (2020). The influence of rare variants in circulating metabolic biomarkers. PLoS Genet. *16*, e1008605.
10. Sun, B.B., Maranville, J.C., Peters, J.E., Stacey, D., Staley, J.R., Blackshaw, J., Burgess, S., Jiang, T., Paige, E., Surendran, P., et al. (2018). Genomic atlas of the human plasma proteome. Nature *558*, 73–79.
11. Li, H., and Durbin, R. (2009). Fast and accurate short read alignment with Burrows-Wheeler transform. Bioinformatics *25*, 1754–1760.
12. McKenna, A., Hanna, M., Banks, E., Sivachenko, A., Cibulskis, K., Kernytsky, A., Garimella, K., Altshuler, D., Gabriel, S., Daly, M., and DePristo, M.A. (2010). The Genome Analysis Toolkit: a MapReduce framework for analyzing next-generation DNA sequencing data. Genome Res. *20*, 1297–1303.
13. Jun, G., Flickinger, M., Hetrick, K.N., Romm, J.M., Doheny, K.F., Abecasis, G.R., Boehnke, M., and Kang, H.M. (2012). Detecting and estimating contamination of human DNA samples in sequencing and array-based genotype data. Am. J. Hum. Genet. *91*, 839–848.
14. Liu, D.J., Peloso, G.M., Zhan, X., Holmen, O.L., Zawistowski, M., Feng, S., Nikpay, M., Auer, P.L., Goel, A., Zhang, H., et al. (2014). Meta-analysis of gene-level tests for rare variant association. Nat. Genet. *46*, 200–204.
15. Richiardi, L., Bellocco, R., and Zugna, D. (2013). Mediation analysis in epidemiology: methods, interpretation and bias. Int. J. Epidemiol. *42*, 1511–1519.
16. Burgess, S., and Thompson, S.G. (2015). Mendelian Randomization: Methods for Using Genetic Variants in Causal Estimation (CRC Press).
17. McInnes, G., Tanigawa, Y., DeBoever, C., Lavertu, A., Olivieri, J.E., Aguirre, M., and Rivas, M.A. (2019). Global Biobank Engine: enabling genotype-phenotype browsing for biobank summary statistics. Bioinformatics *35*, 2495–2497.
18. Taliun, D., Harris, D.N., Kessler, M.D., Carlson, J., Szpiech, Z.A., Torres, R., Taliun, S.A.G., Corvelo, A., Gogarten, S.M., Kang, H.M., et al. (2021). Sequencing of 53,831 diverse genomes from the NHLBI TOPMed Program. Nature *590*, 290–299.
19. Kumar, P., Henikoff, S., and Ng, P.C. (2009). Predicting the effects of coding non-synonymous variants on protein function using the SIFT algorithm. Nat. Protoc. *4*, 1073–1081.
20. Adzhubei, I., Jordan, D.M., and Sunyaev, S.R. (2013). Predicting functional effect of human missense mutations using PolyPhen-2. Curr. Protoc. Hum. Genet. *Chapter 7*, Unit7.20.
21. Kircher, M., Witten, D.M., Jain, P., O'Roak, B.J., Cooper, G.M., and Shendure, J. (2014). A general framework for estimating the relative pathogenicity of human genetic variants. Nat. Genet. *46*, 310–315.
22. Ioannidis, N.M., Rothstein, J.H., Pejaver, V., Middha, S., McDonnell, S.K., Baheti, S., Musolf, A., Li, Q., Holzinger, E., Karyadi, D., et al. (2016). REVEL: an ensemble method for predicting the pathogenicity of rare missense variants. Am. J. Hum. Genet. *99*, 877–885.
23. Di Angelantonio, E., Thompson, S.G., Kaptoge, S., Moore, C., Walker, M., Armitage, J., Ouwehand, W.H., Roberts, D.J., Danesh, J.; and INTERVAL Trial Group (2017). Efficiency and safety of varying the frequency of whole blood donation (INTERVAL): a randomised trial of 45 000 donors. Lancet *390*, 2360–2371.
24. Singh, T., Kurki, M.I., Curtis, D., Purcell, S.M., Crooks, L., McRae, J., Suvisaari, J., Chheda, H., Blackwood, D., Breen, G., et al. (2016). Rare loss-of-function variants in SETD1A are associated with schizophrenia and developmental disorders. Nat. Neurosci. *19*, 571–577.
25. Singh, T., Walters, J.T.R., Johnstone, M., Curtis, D., Suvisaari, J., Torniainen, M., Rees, E., Iyegbe, C., Blackwood, D., McIntosh, A.M., et al. (2017). The contribution of rare variants to risk of schizophrenia in individuals with and without intellectual disability. Nat. Genet. *49*, 1167–1173.
26. MacArthur, D.G., Balasubramanian, S., Frankish, A., Huang, N., Morris, J., Walter, K., Jostins, L., Habegger, L., Pickrell, J.K., Montgomery, S.B., et al. (2012). A systematic survey of loss-of-function variants in human protein-coding genes. Science *335*, 823–828.
27. Morris, A.P., and Zeggini, E. (2010). An evaluation of statistical approaches to rare variant analysis in genetic association studies. Genet. Epidemiol. *34*, 188–193.
28. Madsen, B.E., and Browning, S.R. (2009). A groupwise association test for rare mutations using a weighted sum statistic. PLoS Genet. *5*, e1000384.
29. Price, A.L., Kryukov, G.V., de Bakker, P.I.W., Purcell, S.M., Staples, J., Wei, L.-J., and Sunyaev, S.R. (2010). Pooled association tests for rare variants in exon-resequencing studies. Am. J. Hum. Genet. *86*, 832–838.

30. Wu, M.C., Lee, S., Cai, T., Li, Y., Boehnke, M., and Lin, X. (2011). Rare-variant association testing for sequencing data with the sequence kernel association test. Am. J. Hum. Genet. *89*, 82–93.
31. Lotta, L.A., Pietzner, M., Stewart, I.D., Wittemans, L.B.L., Li, C., Bonelli, R., Raffler, J., Biggs, E.K., Oliver-Williams, C., Auyeung, V.P.W., et al. (2021). A cross-platform approach identifies genetic regulators of human metabolism and health. Nat. Genet. *53*, 54–64.
32. Luo, S., Feofanova, E.V., Tin, A., Tung, S., Rhee, E.P., Coresh, J., Arking, D.E., Surapaneni, A., Schlosser, P., Li, Y., et al. (2021). Genome-wide association study of serum metabolites in the african American study of kidney disease and hypertension. Kidney Int. *100* (2), 430–439.
33. Yazdani, A., Yazdani, A., Elsea, S.H., Schaid, D.J., Kosorok, M.R., Dangol, G., and Samiei, A. (2019). Genome analysis and pleiotropy assessment using causal networks with loss of function mutation and metabolomics. BMC Genomics *20*, 395.
34. Carvalho-Silva, D., Pierleoni, A., Pignatelli, M., Ong, C., Fumis, L., Karamanis, N., Carmona, M., Faulconbridge, A., Hercules, A., McAuley, E., et al. (2019). Open Targets Platform: new developments and updates two years on. Nucleic Acids Res. *47*, D1056–D1065.
35. Stenson, P.D., Ball, E.V., Mort, M., Phillips, A.D., Shiel, J.A., Thomas, N.S.T., Abeysinghe, S., Krawczak, M., and Cooper, D.N. (2003). Human gene mutation database (HGMD): 2003 update. Hum. Mutat. *21*, 577–581.
36. Ferri, L., Funghini, S., Fioravanti, A., Biondi, E.G., la Marca, G., Guerrini, R., Donati, M.A., and Morrone, A. (2014). Aminoacylase I deficiency due to ACY1 mRNA exon skipping. Clin. Genet. *86*, 367–372.
37. Ferkingstad, E., Oddsson, A., Gretarsdottir, S., Benonisdottir, S., Thorleifsson, G., Deaton, A.M., Jonsson, S., Stefansson, O.A., Norddahl, G.L., Zink, F., et al. (2018). Genome-wide association meta-analysis yields 20 loci associated with gallstone disease. Nat. Commun. *9*, 5101.
38. KOMROWER, G.M., WILSON, V., CLAMP, J.R., and WESTALL, R.G. (1964). Hydroxykynureninuria: A case of ABNORMAL tryptophan metabolism probably due to a deficiency OF kynureninase. Arch. Dis. Child. *39*, 250–256.
39. Levi, I., Gurevich, M., Perlman, G., Magalashvili, D., Menascu, S., Bar, N., Godneva, A., Zahavi, L., Chermon, D., Kosower, N., et al. (2021). Potential role of indolelactate and butyrate in multiple sclerosis revealed by integrated microbiome-metabolome analysis. Cell Rep. Med. *2*, 100246.
40. Chen, Y., and Guillemin, G.J. (2009). Kynurenine pathway metabolites in humans: disease and healthy States. Int. J. Tryptophan Res. *2*, 1–19.
41. Rohloff, J.C., Gelinas, A.D., Jarvis, T.C., Ochsner, U.A., Schneider, D.J., Gold, L., and Janjic, N. (2014). Nucleic acid ligands with protein-like side chains: modified aptamers and their use as diagnostic and therapeutic agents. Mol. Ther. Nucleic Acids *3*, e201.
42. Pettersen, E.F., Goddard, T.D., Huang, C.C., Couch, G.S., Greenblatt, D.M., Meng, E.C., and Ferrin, T.E. (2004). UCSF Chimera–a visualization system for exploratory research and analysis. J. Comput. Chem. *25*, 1605–1612.
43. Santas, J., Codony, R., and Rafecas, M. (2013). Phytosterols: beneficial effects. In Natural Products, K.G. Ramawat and J.-M. Mérillon, eds. (Springer Berlin Heidelberg), pp. 3437–3464.
44. Teupser, D., Baber, R., Ceglarek, U., Scholz, M., Illig, T., Gieger, C., Holdt, L.M., Leichtle, A., Greiser, K.H., Huster, D., et al. (2010). Genetic regulation of serum phytosterol levels and risk of coronary artery disease. Circ. Cardiovasc. Genet. *3*, 331–339.
45. Ngo, D., Benson, M.D., Long, J.Z., Chen, Z.-Z., Wang, R., Nath, A.K., Keyes, M.J., Shen, D., Sinha, S., Kuhn, E., et al. (2021). Proteomic profiling reveals biomarkers and pathways in type 2 diabetes risk. JCI Insight *6*, 144392.
46. Ritchie, S.C., Lambert, S.A., Arnold, M., Teo, S.M., Lim, S., Scepanovic, P., Marten, J., Zahid, S., Chaffin, M., Liu, Y., et al. (2021). Integrative analysis of the plasma proteome and polygenic risk of cardiometabolic diseases. Nat. Metab. *3*, 1476–1483.

The American Journal of Human Genetics, Volume *109*

Supplemental information

Whole-exome sequencing identifies rare genetic variants associated with human plasma metabolites

Lorenzo Bomba, Klaudia Walter, Qi Guo, Praveen Surendran, Kousik Kundu, Suraj Nongmaithem, Mohd Anisul Karim, Isobel D. Stewart, Claudia Langenberg, John Danesh, Emanuele Di Angelantonio, David J. Roberts, Willem H. Ouwehand, INTERVAL study, Ian Dunham, Adam S. Butterworth, and Nicole Soranzo

Supplemental Note

In this section we provide an in-depth description of all the gene-metabolite associations with a biological and when possible clinical interpretation, they are ordered by approach (MLOF, LOF, CODING) and metabolite super pathways (amino acids, lipids, carbohydrates, nucleotides, energy, unknown)

MLOF test

Out of the 20 genes, 6 (*ACY1, IVD, KYNU, CCBL1, PAH, NAT8*) were associated with 13 metabolites classified as amino acids.

The association between *ACY1* and N-acetylmethionine is our top hit ($P=2.1\times10^{-24}$) and was identified by the variable threshold test. In total *ACY1* was associated with seven acetyl/formyl amino acids by all family burden tests used in the analysis. A total of 18 variants were included in the test and were associated with increased metabolite level. This gene has been described in OMIM as a gene-causing aminoacylase 1 deficiency (ACY1D), a rare form of inborn error of metabolism characterized by increased urinary excretion of specific N-acetyl amino acids. Most patients show neurologic abnormalities such as intellectual disability, seizures, hypotonia, and motor delay. ACY1D is an ultra-rare autosomal recessive condition with unknown prevalence of affected children and only 15 cases reported world-wide. Diagnosis is made by gas chromatography-mass spectrometry (GC-MS) analysis of urinary organic acids revealing increased levels of N-acetylated amino acids. The prognosis is unknown and the management is symptomatic only. *ACY1* is located on chromosome 3p21 that comprises 15 exons and encodes for aminoacylase-1. Aminoacylase-1 (EC 3.5.1.14) is a homodimeric zinc-binding metalloenzyme, localized in the cytoplasm and expressed in a wide range of tissues. Aminoacylase-1 is the most abundant of the aminoacylases, a class of enzymes involved in hydrolysis of N-acetylated proteins when they are no longer needed. Many proteins in the body have an acetyl group attached to one end, and this modification, called N-acetylation, helps to protect and to stabilize the protein. Aminoacylase 1 performs the final step in the breakdown of these proteins by removing the acetyl group from certain amino acids except L-aspartate. The amino acids can then be recycled and used to build other proteins. N-terminal acetylation of proteins is a widespread and highly conserved process that is involved in the protection and stability of proteins. However, ACY1 can also catalyze the reverse reaction, the synthesis of acetylated amino acids. ACY1 may also play a role in xenobiotic bioactivation as well as the inter-organ processing of amino acid-conjugated xenobiotic [1]derivatives (S-substituted-N-acetyl-L-cysteine). ACY1 appears to physically interact with Sphingosine kinase type 1 (SphK1) and may influence its physiological functions [1]; SphK1 and its product sphingosine-1-phosphate have been shown to promote cell growth and inhibit apoptosis of tumor cells. Overexpression of aminoacylase 1 is associated with colorectal cancer progression [2]. Enzyme knockdown inhibits cell proliferation and causes cell cycle perturbation. Strong expression of the human gene and its mouse orthologous Acy1 in the brain, liver, and kidney, suggest a role of the enzyme in amino acid metabolism of these organs.

The *IVD* gene was associated with an increase in isovalerylcarnitine levels ($P=1.1\times10^{-11}$) including 14 variants. Isovaleryl-CoA dehydrogenase (EC 1.3.99.10) is a member of the acyl-CoA dehydrogenase family and is involved in the catabolism of leucine.

Mutations in *IVD* are reported in OMIM to cause Isovaleric acidemia. It can present with severe neonatal ketoacidosis leading to death, but in milder cases recurrent episodes of ketoacidosis of varying degree occur later in infancy and childhood [3 4].Isovaleric acidemia is an autosomal recessive inborn error of leucine metabolism caused by a deficiency of the mitochondrial enzyme isovaleryl-CoA dehydrogenase (IVD) that results in the accumulation of derivatives of isovalerate, isovalerylglycine, and isovalerylcarnitine. IVD is an enzyme in the L-leucine degradation pathway that specifically catalyzes the conversion of isovaleryl-CoA into 3-methylcrotonyl-CoA. Thus, gene disruption or mutation results in accretion of plasma isovalerylcarnitine (as well as isovalerylglycine and isovalerate) to toxic levels and is the key phenotypic features associated with IVD gene function [5]. The rs28940889[6] variant is one of the variants included in the test and is also reported in OMIM and ClinVar (Isovaleryl-CoA dehydrogenase deficiency;not provided). This could potentially be of interest since this mutation was found in healthy adults.

The *KYNU* gene was associated with increased levels of xanthurenate which is a metabolite from tryptophan catabolism, aka 8-Hydroxykynurenate. This association was identified through a burden test ($P=1.4\times10^{-09}$) including a total of 12 rare variants. Kynureninase (EC 3.7.1.33) is a 3-hydroxykynureninase-type enzyme involved in the kynurenine pathway for the biosynthesis of NAD cofactors from tryptophan. It catalyzes the conversion of L-3-hydroxykynurenine and L-kynurenine to 3-hydroxyanthranilic acid and anthranilic acid, respectively. The reaction is pyridoxal-5-prime-dependent and is sensitive to nutritional vitamin B6 deprivation in mammals. Studies in mouse, rat, and pig suggest that kynureninase is a 95-kD homodimer predominantly located in the cytoplasm [7]. Kynureninase is also involved in the de novo NAD(H) synthesis pathway, using niacin from dietary input [8]. Mutations in *KYNU* have been described to cause hydroxykynureninuria. [9] described a female patient, an only child, who excreted large amounts of kynurenine, 3-hydroxykynurenine, and xanthurenic acid in the urine. Absence of kynureninase resulting in a block in the pathway from tryptophan to nicotinic acid, was postulated. Under these circumstances, tryptophan is no longer a source of nicotinic acid and deficiency of the vitamin can develop. The mother excreted 3 to 4 times the normal amounts of xanthurenic acid. The father's excretion was at the upper limit of normal. The *KYNU* mutation could lead to vertebral, cardiac, renal and limb defects syndrome 2 (VCRL2VCRL2) which is an autosomal recessive congenital malformation syndrome characterized by vertebral segmentation abnormalities, congenital cardiac defects, renal and distal mild limb defects. In two unrelated patients with VCRL2, [8] identified homozygous truncating mutations in the *KYNU* gene. The mutations, which were found by whole-exome sequencing and confirmed by Sanger sequencing, segregated with the disorder in the families. In vitro functional expression studies showed that the mutations essentially abolished any KYNU enzymatic activity. Analysis of plasma from one patient showed increased levels of the upstream metabolite L-3-hydroxykynurenine and decreased levels of the downstream metabolites NAD and NAH(H). Studies in mice, which have different niacin levels compared to humans, indicated that the congenital malformations found in humans resulted from deficient NAD levels rather than increased 3-hydroxyanthranilic acid. [8] noted that NAD is a cofactor with broad cellular effects, including ATP production, macromolecular biosynthesis, redox reactions, energy metabolism, DNA repair and modulation of transcription factors, all of which play an important role in embryogenesis.[8] theorized that niacin supplementation could be of benefit in such patients.

Kynureninase catalyzes the cleavage of L-kynurenine (L-Kyn) and L-3-hydroxykynurenine (L-3OHKyn) into anthranilic acid (AA) and 3-hydroxyanthranilic acid (3-OHAA), respectively in Tryptophan metabolism. It also has cysteine-conjugate-beta-lyase activity. *KYNU* was found to be associated with Encephalopathy due to hydroxykynureninuria in the Open Target platform. None of the included variants were reported previously in either OMIM or the mGWAS server. rs752759030 was found to be weakly associated in the UKBB PheWAS with traits such as ever had stillbirth, spontaneous miscarriage or termination.

CCBL1 was associated with increased levels of indolelactate in a burden test that included 17 rare variants. Indolelactate is a tryptophan metabolite found in human plasma and serum and normal urine. Tryptophan is metabolized by two major pathways in humans, either through kynurenine or via a series of indoles. CCBL1 (aka KYAT1) is part of the Tryptophan catabolism pathway. Cysteine conjugate beta-lyase 1 (CCBL1; glutamine transaminase K; kynurenine aminotransferase; EC 2.6.1.64) metabolizes cysteine conjugates of certain halogenated alkenes and alkanes to form reactive metabolites that can produce nephrotoxicity and neurotoxicity in experimental animals and human. Indolelactate is a regulator of the reaction catalyzed by CCBL1.

Both KYNU and CCBL1 are enzymes of the kynurenine pathway which is often systematically up-regulated when the immune response is activated. The biological significance is that 1) the depletion of tryptophan and generation of kynurenines play a key modulatory role in the immune response; and 2) some of the kynurenines, such as quinolinic acid, 3-hydroxykynurenine and kynurenic acid, are neuroactive.

The kynurenine pathway has been demonstrated to be involved in many diseases and disorders, including Alzheimer's disease, amyotrophic lateral sclerosis, Huntington's disease, AIDS dementia complex, malaria, cancer, depression and schizophrenia, where imbalances in tryptophan and kynurenines have been found [10].

PAH was associated with increased levels of phenylalanine through a Madsen-Browning test ($P=1.7\times10^{-10}$) that contains 11 variants. This is a well-known association and many reported mutations in *PAH* are

causing Phenylketonuria (PKU; 261600) an autosomal recessive inborn error of metabolism resulting from a deficiency of the PAH enzyme. Phenylalanine hydroxylase (PAH; EC 1.14.16.1) catalyzes the hydroxylation of phenylalanine to tyrosine, the rate-limiting step in the phenylalanine catabolism. The reaction is dependent on tetrahydrobiopterin (BH4), as a cofactor, molecular oxygen and iron. If undiagnosed and untreated, phenylketonuria can result in impaired postnatal cognitive development resulting from a neurotoxic effect of hyperphenylalaninemia [11]. Today newborn screening is available for this condition and it is usually treated, maintaining a good standard of living for the patient. Four variants included in the window tested were also reported in OMIM listed variants causing PKU (rs5030860, rs62508646, rs62642937 and rs62644499). All driver variants identified are causing a missense change in the protein, specifically rs5030860 is annotated as pathogenic in ClinVar, and in the homozygous state it causes mild PKU and non-PKU hyperphenylalaninemia (HPA).

NAT8 was associated with decreased levels of N-acetyl arginine through a burden test ($P=8.4\times10^{-10}$) that collapsed 13 variants in a window. *NAT8* was also associated with N-acetyltyrosine, another acetylated amino acid. NAT8 catalyzes the last step of mercapturic acid formation by acetylating cysteine S-conjugates to mercapturic acids [12]. This gene plays an important role in the development and maintenance of normal kidney and liver structure and function. Mercapturic acid synthesis allows detoxification and excretion of cysteinyl conjugates under the form of mercapturic acids. [13] identified an association between rs13391552 in the NAT8 gene and N-acetylornithine levels with a P of 5.4×10^{-252}) that has been also reported by [14]. The N-acetyltransferase function of NAT8 matches the associating metabolite N-acetylornithine, and [13] found associations with glomerular filtration rate (GFR) and chronic kidney disease, represented by association of N-acetylornithine with estimated GFR, in this study. An alternative NAT8 function may be lysine N-acetyltransferase activity catalyzing peptidyl-lysine N6-acetylation of various proteins.

Seven lipid metabolites were associated with 5 genes (*ABCG5, SLC16A9, CERS4, RGS3, ACADS*) making this metabolic class the second most represented in the MLOF approach.

ABCG5 was associated with increased campesterol level in a burden test ($P=1.6\times10^{-08}$) that included 14 variants. The *ABCG5* gene is tandemly arrayed on chromosome 2, in a head-to-head orientation with family member *ABCG8*. ATP binding cassette subfamily G member 5 (ABCG5) encoded by *ABCG5* gene is an ABC transporter involved in the lipid homeostasis pathway transporting sterols from the cytosol to the extracellular domain. ABCG5 functions as a half-transporter to limit intestinal absorption and promote biliary excretion of sterols. It is expressed in a tissue-specific manner in the liver, colon, and intestine. Mutations in this gene may contribute to sterol accumulation and atherosclerosis, and have been observed in patients with phytosterolemia.

Campesterol is a phytosterol (PS), meaning it is a steroid derived from plants. As a food additive, phytosterols have cholesterol-lowering properties (reducing cholesterol absorption in intestines), and may act in cancer prevention. Phytosterols naturally occur in small amounts in vegetable oils, especially soybean oil. One such phytosterol complex, isolated from vegetable oil, is cholestatin, composed of campesterol, stigmasterol, and brassicasterol, and is marketed as a dietary supplement. Sterols can reduce cholesterol in human subjects by up to 15%. The mechanism behind phytosterols and the lowering of cholesterol occurs as follows: the incorporation of cholesterol into micelles in the gastrointestinal tract is inhibited, decreasing the overall amount of cholesterol absorbed. This may in turn help to control body total cholesterol levels, as well as modify HDL, LDL and TAG levels. Many margarines, butters, breakfast cereals and spreads are now enriched with phytosterols and marketed towards people with high cholesterol and a wish to lower it. Moreover, there are different mechanisms of action on how PS can help decreasing levels of plasma cholesterol that are not only limited to competitive solubilization into mixed micelles between cholesterol and PS at the intestinal level as described above, but also i) Esterification of free cholesterol in the enterocyte is reduced by competition with PS for ACAT-2 enzyme; Upregulation of the heterodimer ABCG5/G8 by PS can increase intestinal and hepato-biliar secretion; Upregulation of ABCA1 by PS can increase the incorporation of sterols into nascent HDL; Increased cholesterol excretion via TICE; Although it is not directly mediated by PS, the lower levels of hepatic cholesterol can lead to a lower VLDL secretion and upregulation of LDL receptor, which improves the clearance of plasma cholesterol [15]. In healthy individuals a maximum of 5% of the plant sterol intake is absorbed, resulting in very low levels in plasma

(0.5 mg dL, representing less than 0.5% total neutral sterols in plasma). However, there is a restricted group of patients affected by phytosterolemia, a rare autosomal recessive disease, that can absorb up to 60% of the dietary plant sterols whereas the rate of absorption of cholesterol seems to be normal. Phytosterolemia is characterized by mutations occurring in ABCG genes controlling the efflux of PS at intestinal level and the delivery of PS by the liver. Several studies reported that these patients have PS plasma levels from 18 to 72 mg dL, which represents 7–30% of the total neutral sterols in plasma. With this disease, there is an accumulation of PS not only in plasma but also in adipose tissue, skin, aorta and other tissues. As a result, the main symptoms of this disease include xanthomatosis and atherosclerosis. This accumulation is not only related by hyperabsorption of PS but also by impaired biliary secretion. Some authors have found that phytosterolemia patients present around 20% reduction of PS biliary excretion and around 50% reduction of the whole-body cholesterol synthesis. It has been also found that in phytosterolemic patients, the hepatic conversion of cholesterol to bile acids is blocked, which can result in cholesterol accumulation and atherosclerosis. New interest in the development of phytosterolemia has arisen from the fact that several studies found relationships between anomalous high levels of PS in plasma and CHD in non-phytosterolemic subjects. For instance, Glueck et al. [16] found that plasma cholesterol levels of 7 mmol L and 40 mmol L of PS were associated with a higher deposition of PS in the aorta in seven subjects. Likewise, Salen et al. [17] reported that lethal atherosclerosis is related to increases in plasma PS levels. Therefore, PS levels in phytosterolemia patients must be carefully controlled. A genome-wide association study for serum phytosterols conducted by Teupser et al (2010) [18] identified common variants in ABCG8 and ABO to be strongly associated with serum phytosterol levels and showed concordant and previously unknown associations with coronary artery disease (CAD). Emerging evidence suggests that PS can consequently play an important role in the prevention of several types of cancer such as lung, stomach, prostate, ovarian and breast cancer. One of the first studies suggesting the preventive effect of PS on cancer showed that Seventh-Day Adventists, having a high dietary intake of PS, presented low rates of colon cancer. The PS intake in this population could reach 344 mg day 1, which was considerably higher in comparison with the average intake of the USA population. This preventive effect was mainly attributed to the reduced bile acid excretion of this population after PS intake, as it is known that high levels of bile acids in the bowel can increase the risk of colon cancer. In contrast, contradictory results have been reported with regard to decreased bile acid excretion due to PS intake. Moreover, these findings are limited by the possible modulating effect coming from other components of the diet. Ileostomy studies are more accurate to determine their effect on reducing bile acid levels in the bowel as the variability attributed to side factors can be minimized or even eliminated. Revision of the available studies on this subject reveal that the magnitude of the effect attributed to PS on the reduction of bile acid excretion can be highly dependent on other dietary factors that must be considered. In addition, the effect on bile acid excretion can vary according to the molecule of the PS studied. Some authors have proposed that other mechanisms could be involved in the cancer preventive effect of PS. Awad and Fink [19] proposed a hypothesis based on the inhibition of cell growth through stimulation of apoptosis (programmed cell death). In vitro studies have also shown the inhibitory effect of certain PS on breast and colon-cancer cell cultures and, to a lower extent, on prostate-cancer cells. Another proposed mechanism is based on the capacity of PS to stimulate the sphingomyelin cycle. For instance, sitosterol seems to have a clear in vitro modulatory effect on this cycle. The PS molecule can be incorporated into the cell wall, thus reducing sphingomyelin and increasing ceramide levels in the cell membrane, which can consequently increase cell apoptosis. Finally, changes in testosterone concentrations can also be accounted as an alternative mechanism involved in the prevention of prostate cancer. It has also been reported that diets containing 2% PS reduce the activity of 5 a-reductase in liver and prostate and thus the testosterone levels in plasma. However, this hypothesis has not yet been confirmed in human studies.

SLC16A9 was associated with decreased levels of carnitine in a variable threshold test (9.5×10^{-09}) aggregating 13 variants in a window. Solute carrier family 16, member 9 (SLC16A9) aka Monocarboxylate transporter 9 (MTC9) belongs to a family of monocarboxylate transporters that facilitate diffusion of monocarboxylate across the plasma membrane via a proton-linked transport along a pH gradient [20]. Monocarboxylates, such as lactate and pyruvate, play a central role in cellular metabolism and metabolic communication between tissues. SLC16A9 is involved in drug transport, bile salt and organic anion transport and has been previously shown to be associated with carnitine and uric acid levels. [13] identified rs7094971 in the *SLC16A9* gene as associated with carnitine levels with a P of 3.4 x 10(-14). To follow up this finding, they tested 3H-carnitine uptake by SLC16A9-expressing Xenopus oocytes and found that

SLC16A9 is a pH-independent carnitine efflux transporter, possibly responsible for carnitine efflux from absorptive epithelia into the blood. Insight in Genome-Wide Association of Metabolite Quantitative Traits by Exome Sequence Analyses from The Erasmus Rucphen Family (ERF) study showed that in the ERF population carnitine and uric acid are highly correlated ($r=0.25$, $P=3.93\times10^{-13}$). They found that rs1171614, located in the 5′UTR of SLC16A9, influences the lymphoblast expression of *SLC16A9* in both the GTEX and GEUVADIS databases, indicating that the effect on carnitine level is possible through expression, rather than through the change in protein function. *SLC16A9* was associated with elevated serum uric acid (SUA) levels in recent genome-wide association studies (GWAS) [21]. A missense variant of *SLC16A9* (K258T), rs2242206, was investigated by [22] in relation to gout, a common disease caused by hyperuricemia, which shows elevated serum uric acid (SUA) levels. Gout patients can be divided into those with renal overload (ROL) gout with intestinal urate underexcretion, and those with renal underexcretion (RUE) gout. rs2242206 significantly increased the risk of ROL gout ($P = 0.012$), with an odds ratio (OR) of 1.28 indicating decrease in intestinal urate excretion and therefore providing clues to better understand the pathophysiology of gout. [22]. In another study conducted by Kolz et al. (2009), rs12356193 within *SLC16A9* was associated with DL-carnitine ($P=4.0\times10^{-26}$) and propionyl-L-carnitine ($P=5.0\times10^{-8}$) concentrations, which in turn were associated with serum UA levels ($P=1.4\times10^{-57}$ and $P=8.1\times10^{-54}$, respectively), forming a triangle between SNP, metabolites, and UA levels. These associations highlight additional pathways that are important in the regulation of serum uric acid levels and point towards novel potential targets for pharmacological intervention to prevent or treat hyperuricemia. In addition, these findings strongly support the hypothesis that transport proteins are key in regulating serum uric acid levels [23]. *SLC16A9* amongst others was also reported to be associated with serum urate concentrations in a study that combined data from >140,000 individuals of European ancestry within the Global Urate Genetics Consortium (GUGC) [21].

CERS4 was associated with decreased sphingomyelin (d18:1/20:1, d18:2/20:0) level in a burden test that included 19 variants ($P=6.2\times10^{-14}$). CERS4 was also found associated with sphingomyelin (d18:1/18:1, d18:2/18:0) and stearoyl sphingomyelin (d18:1/18:0). Ceramide, the structural backbone of sphingolipids, is also an important signaling molecule in apoptosis, differentiation, and the cell cycle. Ceramide synthases (EC 2.3.1.24), such as CERS4, are conserved from yeast to mammals and are essential for de novo ceramide synthesis, which involves the formation of an amide linkage between a fatty acyl-CoA and a sphingoid base [24,25]. When overexpressed in cells is involved in the production of sphingolipids containing different fatty acid donors (N-linked stearoyl-(C18) or arachidoyl-(C20) ceramides) in a fumonisin B1-independent manner. [26] provided evidence of an association between activity of phospholipid transfer protein (PLTP; 172425) and coding variation in the CERS4 gene using linkage study. In human studies, the activity of PLTP (PLTPa) is associated with total cholesterol, VLDL and LDL cholesterol, and apoB level. PLTPa is positively correlated with insulin and glycosylated hemoglobin (HbA1C) and decreases in response to insulin infusion [26]. In a genome-wide association studies of European ancestry *CERS4* variant at rs2100944 was associated with higher levels of long-chain saturated fatty acids (VLSFAs) 20:0 carbons ($P = 2.6 \times 10^{-40}$) indicating an inter-relationship of circulating VLSFAs and sphingolipid synthesis.

RGS3 was associated with increase in stearoyl sphingomyelin (d18:1/18:0) level - already reported in this study to be associated with *CERS4* but with opposite direction of effect than *RGS3* - in a variable threshold test ($P=1.6\times10^{-08}$) that included 10 variants. RGS3 is part of what is defined as a 'regulator of G protein signaling' (RGS) domain. Proteins containing the RGS domain constitute a family of molecules that appear to function as negative regulators of heterotrimeric G protein signaling. RGS3 Down-regulates signaling from heterotrimeric G-proteins by increasing the GTPase activity of the alpha subunit, thereby driving them into their inactive GDP-bound form. RGS3 inhibits signaling through the sphingosine 1-phosphate S1P receptor subtypes 1, 2, and 3 [27].

ACADS was associated with increased butyrylcarnitine and ethylmalonate levels in a SKAT test ($P=1.2\times10^{-08}$ and $P=4.7\times10^{-09}$, respectively). Out of the 11 variants included in the test 2 and 4 were associated with a slight reduction in ethylmalonate and butyrylcarnitine indicating the value of using a regression-based model to identify cases where a window contains a number of non-causal or opposite directions of effect. ACADS is aka Butyryl-CoA dehydrogenase and has a biochemical consequence on butyrylcarnitine level that is elevated in patients with acyl-CoA dehydrogenase, short-chain (SCAD) deficiency. SCAD deficiency is an autosomal recessive metabolic disorder of fatty acid beta-oxidation with a prevalence of 1 in 35,000 to 50,000 new-borns. In infants, it causes acute acidosis and generalized muscle weakness and in middle-

aged patients, it causes chronic myopathy localized in muscle. SCAD deficiency is generalized in the former type and localized to skeletal muscles in the latter (OMIM 201470). SCAD prevents the body from converting certain fats into energy, especially during periods without food (fasting). Van Maldegem et al. (2006) found on at least two occasions, increased butyrylcarnitine (C4) concentrations in plasma or bloodspot, and/or increased ethylmalonic acid (EMA) concentrations in urine under non-stressed conditions [28]

Two carbohydrate metabolites were associated with 2 genes (*NPL, SLC5A10*) that recapitulates the biochemical reactions.

NPL was associated with increased N-acetylneuraminate level in a burden test ($P=3.2\times10^{-09}$) that included 15 variants. *NPL* encodes for N-acetylneuraminate pyruvate lyase (EC 4.1.3.3) that controls the cellular concentration of sialic acid by catalyzing the conversion of sialic acid into acylmannosamines and pyruvate [29]. The *NPL* gene encodes a member of the N-acetylneuraminate lyase sub-family that regulate cellular concentrations of N-acetylneuraminate by mediating the reversible aldol condensation between N-acetyl-d-mannosamine (ManNAc) and pyruvate to N-acetylneuraminate. This is an ancient pathway conserved to bacteria and indeed represents a therapeutic target for pathogenic bacteria in humans because of the ability of these species to utilize the carbon sources present in the mucus-rich surfaces. N-acetylneuraminate (sialic acid, NANA, Neu5Ac) is an essential component of complex carbohydrates, which play pivotal roles in recognition processes in a variety of cellular recognition and communication processes, including host-parasite interactions, where the oligosaccharide is often required for invasion, infectivity and survival of the invading organism in the host. N-acetylneuraminate analogues therefore represent attractive targets for novel chemotherapeutic agents against bacterial and viral infections. Thus, there is an obvious link between the *NPL* gene with N-acetylneuraminate availability and suggest that *NPL* genetic variation can have strong influences on susceptibility to bacterial and viral pathogenicity.

SLC5A10 was associated with decreased 1,5-anhydroglucitol (1,5-AG) levels in a burden test ($P=9.2\times10^{-09}$) that included 15 variants. *SLC5A10* encodes for a kidney-specific sodium-dependent sugar transporter that maintains in part 1,5-anhydroglucitol level by active renal uptake. 1,5-anhydroglucitol (1,5-AG) is a non-traditional biomarker of hyperglycemia that is of growing clinical interest. It is a naturally occurring monosaccharide found in nearly all foods and absorbed in the gut. Under normoglycemic conditions, its concentrations in blood are maintained constant through renal filtration followed by reabsorption in the proximal tubules. Glucose and 1,5-AG share some transport proteins for which they represent competing substrates. When blood glucose concentrations exceed the renal glucose threshold of approximately 180 mg/dL, glucose is excreted in the urine and inhibits tubular reabsorption of 1,5-AG, resulting in lower blood 1,5-AG concentrations. Consequently, glucose peaks can lead to decreased 1,5-AG serum concentrations, and 1,5-AG has been established as a marker of hyperglycemic excursions and postprandial glucose peaks. Recent studies have demonstrated robust associations of low serum 1,5-AG concentrations with long-term microvascular and macrovascular complications in persons with diabetes, and with major cardiovascular events in persons without diabetes. These observations are supported by complementary evidence linking daily glucose fluctuations to cardiovascular complications. Gaining insights into the genetic underpinnings of a glycemic marker with unique properties, such as 1,5-AG, may improve our understanding not only of the biology of the marker itself, but also of diabetes, hyperglycemia and glucose metabolism [30]. According to the Human Protein Atlas, *SLC5A10* transcript is exclusively found in the human kidney cortex. The protein is a Na+-dependent transporter of mannose, fructose, galactose and glucose, responsible for their reabsorption from urine in the brush border of renal proximal tubule cells. Because of its exclusive expression in the kidney, genetic variation in this gene is likely related to 1,5-AG concentrations either because it also transports 1,5-AG or because it influences the amount of urinary glucose that competes with 1,5-AG for renal reuptake through *SLC5A9*. The latter protein is thought to be the main renal re-uptake mechanism for 1,5-AG. Of note, we did not observe any association between variants in SLC5A9 and 1,5-AG concentrations in our study, suggesting that SLC5A9 may not be the main transporter for renal 1,5-AG reuptake or that variants impacting SLC5A9 function were not present or detectable in our population. SLC5A9 shows high similarity to SLC5A10, suggesting that SLC5A10 may represent a novel 1,5-AG transport protein [31].

Four nucleotide metabolites involved in purine and pyrimidine metabolism were associated with 3 genes (*PTER, ADSL, UMPS*).

PTER was associated with increased N-acetyl-beta-alanine levels in a Madsen and Browning test ($P=1.9\times10^{-14}$) that included 14 variants. PTER was also associated with N-acetyltaurine in a burden test (5.5×10^{-12}). Microbial phosphotriesterases are a group of zinc metalloenzymes that catalyze the hydrolysis of a range of phosphotriester compounds. By differential cDNA library screening, [32] found that Pter was significantly underexpressed in cystic kidneys of cpk mouse, a model of human autosomal recessive polycystic kidney disease (ARPKD; 263200). Pter expression was significantly decreased upon acute renal injury induced by a single intraperitoneal injection of folic acid, and normal Pter levels returned upon recovery of kidney function. It may be involved in hydrolysing bile acids.

ADSL was associated with increased N6-succinyladenosine levels in the burden test ($P=8.3\times10^{-11}$) that included 13 variants. Adenylosuccinate lyase (ADSL) catalyzes two non-sequential steps in de novo AMP synthesis: converts (S)-2-(5-amino-1-(5-phospho-D-ribosyl)imidazole-4-carboxamido)succinate (SAICAR) to fumarate plus 5-amino-1-(5-phospho-D-ribosyl)imidazole-4-carboxamide, and thereby also contributes to de novo IMP synthesis, and converts succinyladenosine monophosphate (SAMP) to AMP and fumarate. Succinyladenosine (SAdo) is one of the dephosphorylated enzyme substrates that accumulates in body fluids of patients with adenylosuccinate lyase (ADSL) deficiency, the other being 5-amino-4-imidazole-N-succinocarboxamide riboside (SAICAr). ADSL is an inherited metabolic disease characterized by various degrees of psychomotor retardation [33]. The severity of the clinical presentation correlates with a low SAdo/SAICAr ratio in body fluids [34]. Normally Succinyladenosine is not found in blood or CSF but may be detected in trace amounts in urine. Would be interested to know how we found it in blood. And wht is the ratio SAdo/SAICAr.

UMPS was associated with increased orotate level in a SKAT test ($P=1.4\times10^{-9}$) that included 16 variants. *UMPS* encode a bifunctional enzyme with orotate phosphoribosyltransferase (OPRT) and orotidylic decarboxylase (ODC) activity, on chromosome 3q13. In mammalian cells, the last step of pyrimidine nucleotide synthesis involves the conversion of orotate to uridine monophosphate (UMP) and is catalyzed by UMP synthase [35]. This bifunctional enzyme has 2 sequential activities, orotate phosphoribosyltransferase (OPRT; EC 2.4.2.10) and orotidine-5-monophosphate decarboxylase (ODC; EC 4.1.1.23). Compound heterozygous mutation in the UMPS gene can cause orotic aciduria. Orotic aciduria is a rare autosomal recessive disorder characterized by megaloblastic anemia and orotic acid crystalluria that is frequently associated with some degree of physical and mental retardation. These features respond to appropriate pyrimidine replacement therapy, and most cases appear to have a good prognosis. A minority of cases have additional features, particularly congenital malformations and immune deficiencies, which may adversely affect this prognosis. Webster et al. 2001 stated that only 2 cases of orotic aciduria without megaloblastic anemia (OAWA) had been reported [36]. Orotic acid is a minor dietary constituent. Indeed, until it was realized that it could be synthesized by humans, orotic acid was known as vitamin B13. The richest dietary sources are cow's milk and other dairy products as well as root vegetables such as carrots and beets. Dietary intake probably contributes to a basal rate of orotic acid excretion in urine because fasting decreases excretion by ~50%. However, it is now apparent that most urinary orotic acid is synthesized in the body, where it arises as an intermediate in the pathway for the synthesis of pyrimidine nucleotides. Orotic acid is converted to UMP by UMP synthase, a multifunctional protein with both orotate phosphoribosyltransferase and orotidylate decarboxylase activity. The most frequently observed inborn error of pyrimidine nucleotide synthesis is a mutation of the multifunctional protein UMP synthase. This disorder prevents the conversion of orotic acid to UMP and thus to other pyrimidines. As a result, plasma orotic acid accumulates to high concentrations, and increased quantities appear in the urine. Indeed, urinary orotic acid is so markedly increased in individuals harboring a mutation in UMP synthase that orotic acid crystals can form in the urine. The urinary concentration of orotic acid in homozygotes can be of the order of millimoles per millimole creatinine. By comparison, the urinary level in unaffected individuals is ~ 1 umol/mmol creatinine [37]. Potential for therapeutic uses. Pyrimidine synthesis inhibitors are used in active moderate to severe rheumatoid arthritis and psoriatic arthritis, as well as in multiple sclerosis. Examples include Leflunomide and Teriflunomide. One energy metabolite involved in the TCA cycle was associated with the LACTB gene.

LACTB was associated with increased succinylcarnitine level in a variable threshold test ($P=7.6\times10^{-13}$) that included 14 variants. LACTB, is a mitochondrial serine protease that acts as a regulator of mitochondrial lipid metabolism. Acts by decreasing protein levels of PISD, a mitochondrial enzyme that converts phosphatidylserine (PtdSer) to phosphatidylethanolamine (PtdEtn), thereby affecting mitochondrial lipid metabolism [38]. Succinylcarnitine (C4DC) arises from the TCA cycle intermediate succinyl-CoA in plasma and liver. Evidence of the association of LACTB with succinylcarnitine arise from a perturbed hepatic gene expression in transgenic LACTB mice that suggests a role of LACTB in the butanoate/succinate pathway. Moreover, transgenic LACTB mice are obese [39]. Common variants in LACTB have been previously associated with high-density lipoprotein cholesterol measurement in GWAS study and also with succinylcarnitine itself [13,40]. LACTB is also a tumor suppressor that modulates lipid metabolism and cell state [38].

Five unknown metabolites were associated with 3 genes (*COMT, ABCC2, ALB*)

COMT was associated with decreased X - 11593 levels in a SKAT test ($P=9.2\times10^{-09}$) that included 8 variants. Catechol-O-methyltransferase (COMT; EC 2.1.1.6) is one of the major mammalian enzymes involved in the metabolic degradation of catecholamines [41]. COMT catalyzes the transfer of a methyl group from S-adenosyl-methionine (SAM) to a hydroxyl group on a catechol nucleus (e.g., dopamine, norepinephrine, or catechol estrogen) [42]. Catechol-O-methyltransferase (COMT) is one of several enzymes that degrade catecholamines (such as dopamine, epinephrine, and norepinephrine), catecholestrogens, and various substances having a catechol structure. Heterozygous or homozygous variants for the COMT V158M methionine allele slow down the COMT enzyme, affecting the breakdown of dopamine and stress hormones, as well as estrogen metabolism. [43] found the COMT 158val/val genotype to confer a significant risk of worse response after 4–6 weeks of antidepressant treatment in patients with major depression suggesting a potentially beneficial effect of an antidepressant add-on therapy with substances increasing dopamine availability individually tailored according to COMT val158met genotype. The molecular formula of the unnamed compound is $C_7H_{10}O_6$ and is most likely O-methylascorbate based on some previous correlations, but we are still in the process of final confirmation. O-methylascorbate is a known product of ascorbate (vitamin C) O-methylation by COMT [44]. Thus, these observations establish a link between O-methylascorbate blood levels, common genetic variation in the COMT locus, and COMT-mediated liver detoxification processes.

ABCC2 was associated with an increased X - 21467 level in a SKAT test ($P=2\times10^{-09}$) that included 16 variants. *ABCC2* was also associated with X - 21441 in a burden test ($P=2\times10^{-08}$).

ABCC2 belongs to the ATP-binding cassette transporter superfamily and transports endogenous and exogenous anionic conjugates from hepatocytes to bile. ABCC2 is also associated with Dubin-Johnson syndrome, a recessively inherited disorder of bilirubin characterized by a build-up of bilirubin in the bloodstream (hyperbilirubinemia). Within this context, X-21441 is a large compound that has precluded identification of a definitive formula that has both sulfate and glucuronide signatures. There are two formulas which work, assuming a sulfate and glucuronide conjugation, but the most likely one is $C_{27}H_{42}SO_{12}$, that supports a steroid backbone (e.g. sulfate pregnenetriol glucuronide), and likely precludes a bilirubin-associated catabolite. However, there are many combinations and further work is required to elucidate final structural determinants for this biochemical.

ALB was associated with decreased X - 22771 levels in a SKAT test ($P=2\times10^{-09}$) that included 9 variants. *ALB* was also associated with X - 16964 in a SKAT test ($P=4.5\times10^{-09}$). *ALB* is encoding for albumin, which is the major protein of the blood plasma, amounting to 60 to 65% of its total protein. The principal functions of albumin are to support the oncotic pressure, which aids in keeping the blood within the circulation, and to sequester and transport many metabolites within the body, particularly less soluble, hydrophobic ones. It is also an important circulating antioxidant and possesses enzymatic properties [45]. We could speculate that the unknown metabolite could be a byproduct of albumin. Mutation on this gene could cause analbuminemia is a rare autosomal recessive disorder manifested by the presence of a very low amount of circulating serum albumin (Negative beta from our result = -2.443 (0.407)). Affected individuals have few clinical symptoms other than mild edema, hypotension, fatigue, and, occasionally, a peculiar lower body lipodystrophy (mainly in adult females). The most common biochemical finding is a gross hyperlipidemia, with a significant increase in the total and LDL cholesterol concentrations, but normal concentrations of

HDL cholesterol and triglycerides. Analbuminemia often leads to fetal or neonatal death in siblings in families of analbuminemic subjects, which may explain the rarity of the trait [46]. Heterozygotes showed intermediate levels of serum albumin. Familial dysalbuminemic hyperthyroxinemia is an autosomal dominant condition characterized by the presence of a variant serum albumin with preferential affinity for thyroxine (T4) in clinically euthyroid individuals. Individuals have consistently elevated total T4 and elevated or normal free T4 values with normal TSH levels. The condition may be confused with hyperthyroidism or thyroid hormone resistance syndromes, prompting repeated unnecessary laboratory testing and possibly even inappropriate treatment [47].

LOF tests

ERICH6 was associated with decreased glycerophosphorylcholine (GPC) level in a variable threshold test ($P=1.9\times10^{-08}$) that included 5 variants. This gene shows a much greater number of LoF variants compared to the window means for other genes (mean number of variants = 3.056). *ERICH6* is not a well characterized gene and the association with glycerophosphorylcholine (GPC) is not trivial to interpret. All the 5 variants are LoF High confidence from Loftee, but the gene is really tolerant to LoF mutation rvis score 1.82 (97.02%). GPC rises in renal medullary cells in vivo when the medullary interstitial levels of salt and urea to which they are exposed become elevated. High NaCl increases the rate of synthesis of GPC from PC, but high urea does not. PC synthesis in mammalian kidney cells occurs through the Kennedy pathway with choline as one of the starting materials. The synthesis of GPC from PC entails the removal of fatty acids by phospholipase activity. The synthesis of GPC could involve sequential activity of a phospholipase A and a lysophospholipase or, alternatively, activity of a single phospholipase B. The GPC that results is water soluble [48].

CODING tests

CIC was associated with decreased 1-(1-enyl-stearoyl)-2-linoleoyl-GPE (P-18:0/18:2) level in a burden test ($P=2.2\times10^{-08}$) that included 18 variants. CIC is an incompletely understood tissue-specific transcriptional repressor that is highly conserved among metazoan organisms and is required for the normal development of multiple adult structures. CIC is an orthologue of the Drosophila melanogaster capicua gene and functions to transduce receptor tyrosine kinase (RTK) and ERK signaling into gene expression changes through a mechanism termed default repression, wherein CIC protein is bound to target gene promoters or enhancers and inhibits transcription in the absence of signal. Activation of RTK signaling results in the accumulation of activated ERK, which directly binds to and phosphorylates CIC protein [49]. ERK-mediated CIC phosphorylation leads to a rapid relief of repression of CIC target genes, followed by cytoplasmic CIC degradation. CIC is well conserved in mammals and recent evidence suggests that human CIC mediates RTK-dependent responses linked to deregulated growth (cell cycle control), ATP-citrate lyase phosphorylation, reactive oxygen species homeostasis, and bile acid homeostasis [50]. CIC encodes a transcriptional repressor that interacts with ATXN1 [51]. [52] reported 5 patients from 4 families with a neurodevelopmental disorder characterized by delayed development apparent in infancy or the first years of life, variable intellectual disability, often with language delay, and behavioral disorders, including autistic features, attention deficit, and hyperactivity. Some patients showed developmental regression early in life and/or learning difficulties. Three patients had seizures, including blinking and staring episodes, myoclonic seizures, complex partial seizures, and absence seizures. Two patients had hypotonia, and 3 had nonspecific T2-weighted white matter abnormalities on brain imaging. Since this gene encodes a DNA binding protein and not an enzyme, the genetic association with the plasmalogen 1-(1-enyl-stearoyl)-2-linoleoyl-GPE (P-18:0/18:2)* is less tractable and can involve regulation of a number of genes in the synthesis and degradation of plasmalogens [53]. However, future work can focus on the specific set of genes that CIC could potentially regulate in plasmalogen synthesis. Perhaps it could be due to the neighboring gene platelet activating factor acetylhydrolase 1b catalytic subunit 3 which has a role in hydrolyzing phospholipids at the golgi.

TYMP was associated with increased 5-methyluridine level in a SKAT test ($P=3.4\times10^{-09}$) that included 10 variants. *TYMP* encodes thymidine phosphorylase (EC 2.4.2.4), a cytosolic enzyme that catalyzes the phosphorylation of thymidine or deoxyuridine to thymine or uracil, and is thus essential for the nucleotide salvage pathway [54]. The protein product was originally identified as platelet-derived endothelial cell growth

factor (PDECGF), an angiogenic factor distinct from the previously described endothelial cell mitogens of the fibroblast growth factor family [55]. PDECGF is stored in platelets as a 45-kD single polypeptide chain and has a highly restricted target cell specificity acting only on endothelial cells. It promotes angiogenesis in vivo, and stimulates the in vitro growth of a variety of endothelial cells. Homozygous or compound heterozygous mutation in the nuclear-encoded TYMP on chromosome 22q13 causes mitochondrial DNA depletion syndrome-1 (MTDPS1), which manifests as a neurogastrointestinal encephalopathy (MNGIE). Mitochondrial DNA depletion syndrome-1 (MTDPS1) is an autosomal recessive progressive multisystem disorder clinically characterized by onset between the second and fifth decades of life of ptosis, progressive external ophthalmoplegia (PEO), gastrointestinal dysmotility (often pseudo obstruction), cachexia, diffuse leukoencephalopathy, peripheral neuropathy, and mitochondrial dysfunction. Mitochondrial DNA abnormalities can include depletion, deletion, and point mutations [56].

CHKB was associated with an increased 5-methyluridine level in a SKAT test ($P=4.8\times10^{-09}$) that included 14 variants. CHKB encoded for choline kinases (EC 2.7.1.32) that catalyze phosphorylation of choline by ATP in the presence of Mg(2+), yielding phosphocholine and ADP. This step commits choline to the enzymatic pathway for biosynthesis of phosphatidylcholine [57,58]. Choline kinase in mammals is encoded by two genes, CHKA and CHKB. These enzymes catalyze the phosphorylation of choline and ethanolamine to phosphocholine and phosphoethanolamine. As a class, these are the first enzymes in the biosynthesis of phosphatidylcholine and phosphatidylethanolamine in all animal cells. Disruption of murine CHKA leads to embryonic lethality, whereas a spontaneous genomic deletion in murine CHKB results in neonatal forelimb bone deformity and hindlimb muscular dystrophy. Studies have found that CHKB is the major isoform in murine hindlimb muscle and contributes more to choline kinase activity. 5-methyluridine, also called ribothymidine, is an endogenous methylated nucleoside resulting from the m5U methyltransferase from uridine substrate with SAM as the methyl donor. Choline and pyrimidine metabolism are required components of phospholipid metabolism, thus genetic association of CHKB with 5-methyluridine may be due to perturbation of cytidine and CTP utilization in phosphatidylcholine synthesis.

CR1L was associated with decreased X - 21444 level in a Madsen and Browning test ($P=1.8\times10^{-08}$) that included 11 variants. The C3b/C4b receptor (CR1) is important in immune complex processing. On phagocytic cells it promotes the adherence and sometimes the endocytosis of C3b- and C4b-coated particles. CR1 is composed largely of a tandemly repeated motif of approximately 60 amino acids, the short consensus repeat (SCR). In the course of cloning genomic CR1 sequences, [59] identified a related gene, CR1L. Analysis of subclones identified 10 exons encoding a signal peptide and SCR1 through SCR6 and SCR9, which are 91% homologous to the corresponding N-terminal regions of the CR1 protein. [59] concluded that CR1L arose by a gene duplication event and has undergone mutation at the beginning of SCR1. The unnamed metabolite has a very tentative formula of $C_4H_6O_3$ with a compound at 102m/z. We will use the information of an association with the complement component (3b/4b) receptor 1-like gene to guide us in retiring this compound, but there is insufficient information to come up with rational annotation for this molecule.

SLC25A15 was associated with decreased X - 15728 levels in a burden test ($P=4.8\times10^{-09}$) that included 18 variants. SLC25A15 encodes the mitochondrial ornithine transporter, which transports ornithine across the inner mitochondrial membrane from the cytosol to the mitochondrial matrix. This is a vital step in the urea cycle, which serves to eliminate toxic ammonium ions from the breakdown of nitrogen [60]. [61] reported a child with mental retardation and myoclonic seizures associated with hyperornithinemia, hyperammonemia, and homocitrullinemia. The findings were consistent with an inherited disorder of amino acid metabolism. The protein transports ornithine across the inner mitochondrial membrane, from the cytoplasm to the matrix. Metabolite likely to be related to ornithine, citrulline of urea cycle.

DPRC1 was associated with an increased 2-aminobutyrate level in a burden test ($P=2.5\times10^{-09}$) that included 16 variants. The *DPCR1* gene was mapped to the HLA class I region and subsequently linked among other 8 genes to diffuse panbronchiolitis (DPB). Diffuse panbronchiolitis (DPB) is a rare chronic inflammatory obstructive pulmonary disease primarily affecting the respiratory bronchioles [62].

In summary, we identified a total of 6 genes associated with 13 metabolites classified as amino acids, 3 as nucleotides with 4 metabolites, 5 as lipids with 7 metabolites, 2 as carbohydrate 2 metabolites, 1 as energy, 3 as unknown 5 metabolites.

Acknowledgements

This research was supported by (1) Wellcome Trust Grant number 206194. For the purpose of Open Access, the author has applied a CC BY public copyright license to any Author Accepted Manuscript version arising from this submission; (2) Open Targets; (3) Participants in the INTERVAL randomized controlled trial were recruited with the active collaboration of NHS Blood and Transplant England (www.nhsbt.nhs.uk [nhsbt.nhs.uk]), which has supported field work and other elements of the trial. DNA extraction and genotyping were co-funded by the National Institute for Health Research (NIHR), the NIHR BioResource (http://bioresource.nihr.ac.uk [bioresource.nihr.ac.uk]) and the NIHR Cambridge Biomedical Research Center (BRC-1215-20014) [*]. Sequencing of the INTERVAL data was supported by the Wellcome Trust grant number 206194. The academic coordinating center for INTERVAL was supported by core funding from the: NIHR Blood and Transplant Research Unit in Donor Health and Genomics (NIHR BTRU-2014-10024), UK Medical Research Council (MR/L003120/1), British Heart Foundation (SP/09/002; RG/13/13/30194; RG/18/13/33946) and NIHR Cambridge BRC (BRC-1215-20014) [*]. A complete list of the investigators and contributors to the INTERVAL trial is provided in reference [23]**. The academic coordinating center would like to thank blood donor center staff and blood donors for participating in the INTERVAL trial.; (4) Health Data Research UK, which is funded by the UK Medical Research Council, Engineering and Physical Sciences Research Council, Economic and Social Research Council, Department of Health and Social Care (England), Chief Scientist Office of the Scottish Government Health and Social Care Directorates, Health and Social Care Research and Development Division (Welsh Government), Public Health Agency (Northern Ireland), British Heart Foundation and Wellcome. (5) Metabolon Metabolomics assays were funded by the NIHR BioResource and the NIHR Cambridge Biomedical Research Center (BRC-1215-20014) [*]. (6) SomaLogic assays were funded by Merck and the NIHR Cambridge Biomedical Research Center (BRC-1215-20014) [*]. (7) Nightingale Health NMR assays were funded by the European Commission Framework Programme 7 (HEALTH-F2-2012-279233). (8) Professor John Danesh holds a British Heart Foundation Professorship and a NIHR Senior Investigator Award [*].
*The views expressed are those of the author(s) and not necessarily those of the NIHR or the Department of Health and Social Care.
**Di Angelantonio E, Thompson SG, Kaptoge SK, Moore C, Walker M, Armitage J, Ouwehand WH, Roberts DJ, Danesh J, INTERVAL Trial Group. Efficiency and safety of varying the frequency of whole blood donation (INTERVAL): a randomized trial of 45,000 donors. Lancet. 2017 Nov 25;390(10110):2360-2371.[63]
We would like to thank Lorenz Wernisch for useful discussions and statistical advice, and we would like to thank three anonymous reviewers for insightful suggestions.

Supplemental Figures and Legends

Figure S1: Distribution of number of variants per window in each strategy.

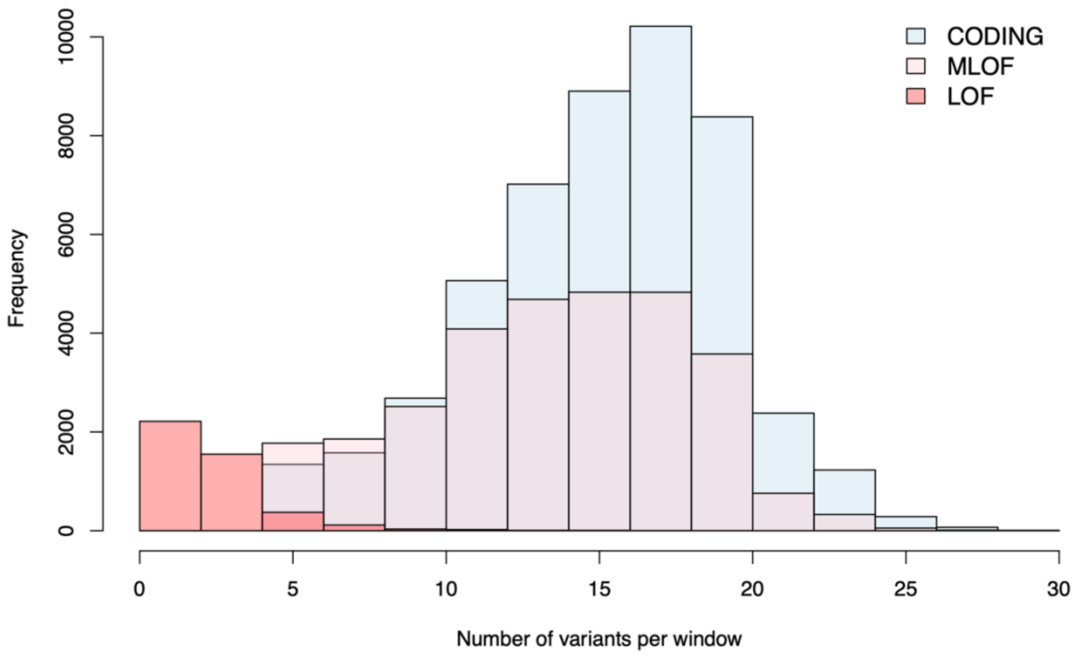

Figure S2: a) Number of driver variants shared using a forward selection approach and Lasso and b) percentage of total number of variants.

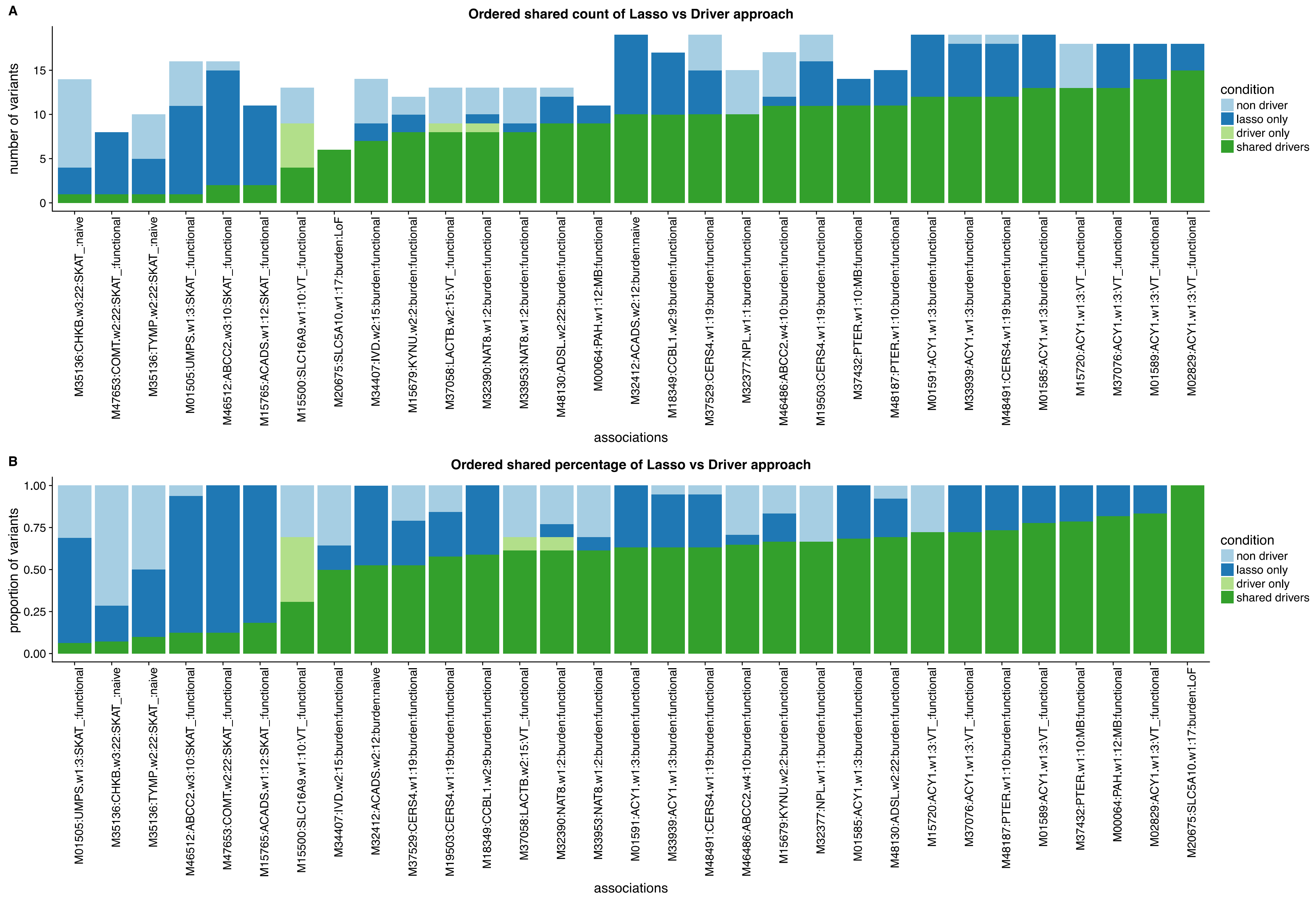
A
Ordered shared count of Lasso vs Driver approach
number of variants
0
5
10
15
M35136:CHKB.w3:22:SKAT_:naive
M47653:COMT.w2:22:SKAT_:functional
M35136:TYMP.w2:22:SKAT_:naive
M01505:UMPS.w1:3:SKAT_:functional
M46512:ABCC2.w3:10:SKAT_:functional
M15765:ACADS.w1:12:SKAT_:functional
M15500:SLC16A9.w1:10:VT_:functional
M20675:SLC5A10.w1:17:burden:LoF
M34407:IVD.w2:15:burden:functional
M15679:KYNU.w2:2:burden:functional
M37058:LACTB.w2:15:VT_:functional
M32390:NAT8.w1:2:burden:functional
M33953:NAT8.w1:2:burden:functional
M48130:ADSL.w2:22:burden:functional
M00064:PAH.w1:12:MB:functional
M32412:ACADS.w2:12:burden:naive
M18349:CCBL1.w2:9:burden:functional
M37529:CERS4.w1:19:burden:functional
M32377:NPL.w1:1:burden:functional
M46486:ABCC2.w4:10:burden:functional
M19503:CERS4.w1:19:burden:functional
M37432:PTER.w1:10:MB:functional
M48187:PTER.w1:10:burden:functional
M01591:ACY1.w1:3:burden:functional
M33939:ACY1.w1:3:burden:functional
M48491:CERS4.w1:19:burden:functional
M01585:ACY1.w1:3:burden:functional
M15720:ACY1.w1:3:VT_:functional
M37076:ACY1.w1:3:VT_:functional
M01589:ACY1.w1:3:VT_:functional
M02829:ACY1.w1:3:VT_:functional
associations
condition
non driver
lasso only
driver only
shared drivers
B
Ordered shared percentage of Lasso vs Driver approach
proportion of variants
0.00
0.25
0.50
0.75
1.00
M01505:UMPS.w1:3:SKAT_:functional
M35136:CHKB.w3:22:SKAT_:naive
M35136:TYMP.w2:22:SKAT_:naive
M46512:ABCC2.w3:10:SKAT_:functional
M47653:COMT.w2:22:SKAT_:functional
M15765:ACADS.w1:12:SKAT_:functional
M15500:SLC16A9.w1:10:VT_:functional
M34407:IVD.w2:15:burden:functional
M32412:ACADS.w2:12:burden:naive
M37529:CERS4.w1:19:burden:functional
M19503:CERS4.w1:19:burden:functional
M18349:CCBL1.w2:9:burden:functional
M37058:LACTB.w2:15:VT_:functional
M32390:NAT8.w1:2:burden:functional
M33953:NAT8.w1:2:burden:functional
M01591:ACY1.w1:3:burden:functional
M33939:ACY1.w1:3:burden:functional
M48491:CERS4.w1:19:burden:functional
M46486:ABCC2.w4:10:burden:functional
M15679:KYNU.w2:2:burden:functional
M32377:NPL.w1:1:burden:functional
M01585:ACY1.w1:3:burden:functional
M48130:ADSL.w2:22:burden:functional
M15720:ACY1.w1:3:VT_:functional
M37076:ACY1.w1:3:VT_:functional
M48187:PTER.w1:10:burden:functional
M01589:ACY1.w1:3:VT_:functional
M37432:PTER.w1:10:MB:functional
M00064:PAH.w1:12:MB:functional
M02829:ACY1.w1:3:VT_:functional
M20675:SLC5A10.w1:17:burden:LoF
associations
condition
non driver
lasso only
driver only
shared drivers

Supplemental tables

Table S1: Study participant characteristics expressed in counts and percentages.

Table S2: Linear regressions of the first five principal components against age, sex, BMI, current smoking, alcohol consumption frequency, center, batch, plate, appointment month and time between appointment and processing.

Table S3: Study metabolite counts per pathway categories.

Table S4: Summary statistics of different strategies used in the study such as number of genes tested in each strategy.

Table S5: Table of all gene-metabolite associations including variants used in the test, type of test and strategy used, whether variants were drivers or not, recalculated Ps when including only driver variants, gene reported in previous mGWAS and OMIM annotation. Also includes overall association results for window-based and gene-based analysis.

Table S6: WGS replications of discovery signals.

Table S7: Summary statistics of associations of previously identified genes (Metabolon, this study) with NMR measured metabolites levels.

Table S8: Conditional analysis of RV discoveries with sentinel variants from imputed data.

Table S9: Biological annotation of rare variant test (RVT) signals

Table S10: Details of all associations discovered based on strategies and test categories.

Supplemental References

1. Maceyka, M., Nava, V.E., Milstien, S., and Spiegel, S. (2004). Aminoacylase 1 is a sphingosine kinase 1-interacting protein. FEBS Lett. *568*, 30–34.

2. Shi, H., Hayes, M.T., Kirana, C., Miller, R.J., Keating, J.P., and Stubbs, R.S. (2013). Overexpression of aminoacylase 1 is associated with colorectal cancer progression. Hum. Pathol. *44*, 1089–1097.

3. Vockley, J., Parimoo, B., and Tanaka, K. (1991). Molecular characterization of four different classes of mutations in the isovaleryl-CoA dehydrogenase gene responsible for isovaleric acidemia. Am. J. Hum. Genet. *49*, 147–157.

4. Kılıç, M., Kaymaz, N., and Özgül, R.K. (2014). Isovaleric acidemia presenting as diabetic ketoacidosis: a case report. J. Clin. Res. Pediatr. Endocrinol. *6*, 59–61.

5. Vockley, J., and Ensenauer, R. (2006). Isovaleric acidemia: new aspects of genetic and phenotypic heterogeneity. Am. J. Med. Genet. C Semin. Med. Genet. *142C*, 95–103.

6. Vockley, J., and Ensenauer, R. (2006). Isovaleric acidemia: new aspects of genetic and phenotypic heterogeneity. Am. J. Med. Genet. C Semin. Med. Genet. *142C*, 95–103.

7. Toma, S., Nakamura, M., Toné, S., Okuno, E., Kido, R., Breton, J., Avanzi, N., Cozzi, L.,

Speciale, C., Mostardini, M., et al. (1997). Cloning and recombinant expression of rat and human kynureninase. FEBS Lett. *408*, 5–10.

8. Shi, H., Enriquez, A., Rapadas, M., Martin, E.M.M.A., Wang, R., Moreau, J., Lim, C.K., Szot, J.O., Ip, E., Hughes, J.N., et al. (2017). NAD Deficiency, Congenital Malformations, and Niacin Supplementation. N. Engl. J. Med. *377*, 544–552.

9. Komrower, G.M., Wilson, V., Clamp, J.R., and Westall, R.G. (1964). HYDROXYKYNURENINURIA: A CASE OF ABNORMAL TRYPTOPHAN METABOLISM PROBABLY DUE TO A DEFICIENCY OF KYNURENINASE. Arch. Dis. Child. *39*, 250–256.

10. Chen, Y., and Guillemin, G.J. (2009). Kynurenine pathway metabolites in humans: disease and healthy States. Int. J. Tryptophan Res. *2*, 1–19.

11. Zurflüh, M.R., Zschocke, J., Lindner, M., Feillet, F., Chery, C., Burlina, A., Stevens, R.C., Thöny, B., and Blau, N. (2008). Molecular genetics of tetrahydrobiopterin-responsive phenylalanine hydroxylase deficiency. Hum. Mutat. *29*, 167–175.

12. Veiga-da-Cunha, M., Tyteca, D., Stroobant, V., Courtoy, P.J., Opperdoes, F.R., and Van Schaftingen, E. (2010). Molecular identification of NAT8 as the enzyme that acetylates cysteine S-conjugates to mercapturic acids. J. Biol. Chem. *285*, 18888–18898.

13. Suhre, K., Shin, S.-Y., Petersen, A.-K., Mohney, R.P., Meredith, D., Wägele, B., Altmaier, E., CARDIoGRAM, Deloukas, P., Erdmann, J., et al. (2011). Human metabolic individuality in biomedical and pharmaceutical research. Nature *477*, 54–60.

14. Shin, S.-Y., Fauman, E.B., Petersen, A.-K., Krumsiek, J., Santos, R., Huang, J., Arnold, M., Erte, I., Forgetta, V., Yang, T.-P., et al. (2014). An atlas of genetic influences on human blood metabolites. Nat. Genet. *46*, 543–550.

15. Santas, J., Codony, R., and Rafecas, M. (2013). Phytosterols: Beneficial Effects. In Natural Products, K.G. Ramawat, and J.-M. Mérillon, eds. (Berlin, Heidelberg: Springer Berlin Heidelberg), pp. 3437–3464.

16. Glueck, C.J., Streicher, P., and Illig, E. (1992). Serum and dietary phytosterols, cholesterol, and coronary heart disease in hyperphytosterolemic probands. Clin. Biochem. *25*, 331–334.

17. Salen, G., Horak, I., Rothkopf, M., Cohen, J.L., Speck, J., Tint, G.S., Shore, V., Dayal, B., Chen, T., and Shefer, S. (1985). Lethal atherosclerosis associated with abnormal plasma and tissue sterol composition in sitosterolemia with xanthomatosis. J. Lipid Res. *26*, 1126–1133.

18. Teupser, D., Baber, R., Ceglarek, U., Scholz, M., Illig, T., Gieger, C., Holdt, L.M., Leichtle, A., Greiser, K.H., Huster, D., et al. (2010). Genetic regulation of serum phytosterol levels and risk of coronary artery disease. Circ. Cardiovasc. Genet. *3*, 331–339.

19. Awad, A.B., and Fink, C.S. (2000). Phytosterols as anticancer dietary components: evidence and mechanism of action. J. Nutr. *130*, 2127–2130.

20. Halestrap, A.P., and Price, N.T. (1999). The proton-linked monocarboxylate transporter (MCT) family: structure, function and regulation. Biochem. J *343 Pt 2*, 281–299.

21. Köttgen, A., Albrecht, E., Teumer, A., Vitart, V., Krumsiek, J., Hundertmark, C., Pistis, G., Ruggiero, D., O'Seaghdha, C.M., Haller, T., et al. (2013). Genome-wide association analyses

identify 18 new loci associated with serum urate concentrations. Nat. Genet. *45*, 145–154.

22. Nakayama, A., Matsuo, H., Shimizu, T., Ogata, H., Takada, Y., Nakashima, H., Nakamura, T., Shimizu, S., Chiba, T., Sakiyama, M., et al. (2013). Common missense variant of monocarboxylate transporter 9 (MCT9/SLC16A9) gene is associated with renal overload gout, but not with all gout susceptibility. Hum. Cell *26*, 133–136.

23. Kolz, M., Johnson, T., Sanna, S., Teumer, A., Vitart, V., Perola, M., Mangino, M., Albrecht, E., Wallace, C., Farrall, M., et al. (2009). Meta-analysis of 28,141 individuals identifies common variants within five new loci that influence uric acid concentrations. PLoS Genet. *5*, e1000504.

24. Riebeling, C., Allegood, J.C., Wang, E., Merrill, A.H., Jr, and Futerman, A.H. (2003). Two mammalian longevity assurance gene (LAG1) family members, trh1 and trh4, regulate dihydroceramide synthesis using different fatty acyl-CoA donors. J. Biol. Chem. *278*, 43452–43459.

25. Venkataraman, K., and Futerman, A.H. (2002). Do longevity assurance genes containing Hox domains regulate cell development via ceramide synthesis? FEBS Lett. *528*, 3–4.

26. Rosenthal, E.A., Ronald, J., Rothstein, J., Rajagopalan, R., Ranchalis, J., Wolfbauer, G., Albers, J.J., Brunzell, J.D., Motulsky, A.G., Rieder, M.J., et al. (2011). Linkage and association of phospholipid transfer protein activity to LASS4. J. Lipid Res. *52*, 1837–1846.

27. Brinkmann, V. (2007). Sphingosine 1-phosphate receptors in health and disease: mechanistic insights from gene deletion studies and reverse pharmacology. Pharmacol. Ther. *115*, 84–105.

28. van Maldegem, B.T., Duran, M., Wanders, R.J.A., Niezen-Koning, K.E., Hogeveen, M., Ijlst, L., Waterham, H.R., and Wijburg, F.A. (2006). Clinical, biochemical, and genetic heterogeneity in short-chain acyl-coenzyme A dehydrogenase deficiency. JAMA *296*, 943–952.

29. Wu, M., Gu, S., Xu, J., Zou, X., Zheng, H., Jin, Z., Xie, Y., Ji, C., and Mao, Y. (2005). A novel splice variant of human gene NPL, mainly expressed in human liver, kidney and peripheral blood leukocyte. DNA Seq. *16*, 137–142.

30. Dungan, K.M. (2008). 1,5-anhydroglucitol (GlycoMark) as a marker of short-term glycemic control and glycemic excursions. Expert Rev. Mol. Diagn. *8*, 9–19.

31. Long, T., Hicks, M., Yu, H.-C., Biggs, W.H., Kirkness, E.F., Menni, C., Zierer, J., Small, K.S., Mangino, M., Messier, H., et al. (2017). Whole-genome sequencing identifies common-to-rare variants associated with human blood metabolites. Nat. Genet. *49*, 568–578.

32. Hou, X., Maser, R.L., Magenheimer, B.S., and Calvet, J.P. (1996). A mouse kidney- and liver-expressed cDNA having homology with a prokaryotic parathion hydrolase (phosphotriesterase)-encoding gene: abnormal expression in injured and polycystic kidneys. Gene *168*, 157–163.

33. Zikánová, M., Krijt, J., Hartmannová, H., and Kmoch, S. (2005). Preparation of 5-amino-4-imidazole-N-succinocarboxamide ribotide, 5-amino-4-imidazole-N-succinocarboxamide riboside and succinyladenosine, compounds usable in diagnosis and research of adenylosuccinate lyase deficiency. J. Inherit. Metab. Dis. *28*, 493–499.

34. Marinaki, A.M., Champion, M., Kurian, M.A., Simmonds, H.A., Marie, S., Vincent, M.F., van den Berghe, G., Duley, J.A., and Fairbanks, L.D. (2004). Adenylosuccinate lyase deficiency--first British case. Nucleosides Nucleotides Nucleic Acids *23*, 1231–1233.

35. McClard, R.W., Black, M.J., Livingstone, L.R., and Jones, M.E. (1980). Isolation and initial characterization of the single polypeptide that synthesizes uridine 5'-monophosphate from orotate in Ehrlich ascites carcinoma. Purification by tandem affinity chromatography of uridine-5'-monophosphate synthase. Biochemistry *19*, 4699–4706.

36. Bailey, C.J. (2009). Orotic aciduria and uridine monophosphate synthase: a reappraisal. J. Inherit. Metab. Dis. *32 Suppl 1*, S227–S233.

37. Brosnan, M.E., and Brosnan, J.T. (2007). Orotic acid excretion and arginine metabolism. J. Nutr. *137*, 1656S – 1661S.

38. Keckesova, Z., Donaher, J.L., De Cock, J., Freinkman, E., Lingrell, S., Bachovchin, D.A., Bierie, B., Tischler, V., Noske, A., Okondo, M.C., et al. (2017). LACTB is a tumour suppressor that modulates lipid metabolism and cell state. Nature *543*, 681–686.

39. Yang, X., Deignan, J.L., Qi, H., Zhu, J., Qian, S., Zhong, J., Torosyan, G., Majid, S., Falkard, B., Kleinhanz, R.R., et al. (2009). Validation of candidate causal genes for obesity that affect shared metabolic pathways and networks. Nat. Genet. *41*, 415–423.

40. Willer, C.J., Schmidt, E.M., Sengupta, S., Peloso, G.M., Gustafsson, S., Kanoni, S., Ganna, A., Chen, J., Buchkovich, M.L., Mora, S., et al. (2013). Discovery and refinement of loci associated with lipid levels. Nat. Genet. *45*, 1274–1283.

41. Gogos, J.A., Morgan, M., Luine, V., Santha, M., Ogawa, S., Pfaff, D., and Karayiorgou, M. (1998). Catechol-O-methyltransferase-deficient mice exhibit sexually dimorphic changes in catecholamine levels and behavior. Proc. Natl. Acad. Sci. U. S. A. *95*, 9991–9996.

42. Chen, J., Lipska, B.K., Halim, N., Ma, Q.D., Matsumoto, M., Melhem, S., Kolachana, B.S., Hyde, T.M., Herman, M.M., Apud, J., et al. (2004). Functional analysis of genetic variation in catechol-O-methyltransferase (COMT): effects on mRNA, protein, and enzyme activity in postmortem human brain. Am. J. Hum. Genet. *75*, 807–821.

43. Baune, B.T., Hohoff, C., Berger, K., Neumann, A., Mortensen, S., Roehrs, T., Deckert, J., Arolt, V., and Domschke, K. (2008). Association of the COMT val158met variant with antidepressant treatment response in major depression. Neuropsychopharmacology *33*, 924–932.

44. Bowers-Komro, D.M., McCormick, D.B., King, G.A., Sweeny, J.G., and Iacobucci, G.A. (1982). Confirmation of 2-O-methyl ascorbic acid as the product from the enzymatic methylation of L-ascorbic acid by catechol-O-methyltransferase. Int. J. Vitam. Nutr. Res. *52*, 186–193.

45. Minchiotti, L., Galliano, M., Kragh-Hansen, U., and Peters, T., Jr (2008). Mutations and polymorphisms of the gene of the major human blood protein, serum albumin. Hum. Mutat. *29*, 1007–1016.

46. Caridi, G., Maout, A., Artan, R., Campagnoli, M., Lugani, F., Abada, M.E.A., Sayar, E., Galliano, M., and Minchiotti, L. (2018). Congenital Analbuminemia in Unrelated Algerian and Turkish Families is Caused by the Same Molecular Defect in the Albumin Gene. Ann. Lab. Med.

38, 185–188.

47. Heufelder, A.E., Klee, G.G., Wynne, A.G., and Gharib, H. (1995). Familial dysalbuminemic hyperthyroxinemia: cumulative experience in 29 consecutive patients. Endocr. Pract. *1*, 4–8.

48. Gallazzini, M., and Burg, M.B. (2009). What's new about osmotic regulation of glycerophosphocholine. Physiology *24*, 245–249.

49. Astigarraga, S., Grossman, R., Díaz-Delfín, J., Caelles, C., Paroush, Z. 'ev, and Jiménez, G. (2007). A MAPK docking site is critical for downregulation of Capicua by Torso and EGFR RTK signaling. EMBO J. *26*, 668–677.

50. Jiménez, G., Shvartsman, S.Y., and Paroush, Z. 'ev (2012). The Capicua repressor--a general sensor of RTK signaling in development and disease. J. Cell Sci. *125*, 1383–1391.

51. Bergeron, D., Lapointe, C., Bissonnette, C., Tremblay, G., Motard, J., and Roucou, X. (2013). An out-of-frame overlapping reading frame in the ataxin-1 coding sequence encodes a novel ataxin-1 interacting protein. J. Biol. Chem. *288*, 21824–21835.

52. Lu, H.-C., Tan, Q., Rousseaux, M.W.C., Wang, W., Kim, J.-Y., Richman, R., Wan, Y.-W., Yeh, S.-Y., Patel, J.M., Liu, X., et al. (2017). Disruption of the ATXN1-CIC complex causes a spectrum of neurobehavioral phenotypes in mice and humans. Nat. Genet. *49*, 527–536.

53. Braverman, N.E., and Moser, A.B. (2012). Functions of plasmalogen lipids in health and disease. Biochim. Biophys. Acta *1822*, 1442–1452.

54. Suomalainen, A., and Isohanni, P. (2010). Mitochondrial DNA depletion syndromes--many genes, common mechanisms. Neuromuscul. Disord. *20*, 429–437.

55. Ishikawa, F., Miyazono, K., Hellman, U., Drexler, H., Wernstedt, C., Hagiwara, K., Usuki, K., Takaku, F., Risau, W., and Heldin, C.H. (1989). Identification of angiogenic activity and the cloning and expression of platelet-derived endothelial cell growth factor. Nature *338*, 557–562.

56. Taanman, J.-W., Daras, M., Albrecht, J., Davie, C.A., Mallam, E.A., Muddle, J.R., Weatherall, M., Warner, T.T., Schapira, A.H.V., and Ginsberg, L. (2009). Characterization of a novel TYMP splice site mutation associated with mitochondrial neurogastrointestinal encephalomyopathy (MNGIE). Neuromuscul. Disord. *19*, 151–154.

57. Ishidate, K. (1997). Choline/ethanolamine kinase from mammalian tissues. Biochim. Biophys. Acta *1348*, 70–78.

58. Aoyama, C., Yamazaki, N., Terada, H., and Ishidate, K. (2000). Structure and characterization of the genes for murine choline/ethanolamine kinase isozymes alpha and beta. J. Lipid Res. *41*, 452–464.

59. Hourcade, D., Miesner, D.R., Bee, C., Zeldes, W., and Atkinson, J.P. (1990). Duplication and divergence of the amino-terminal coding region of the complement receptor 1 (CR1) gene. An example of concerted (horizontal) evolution within a gene. J. Biol. Chem. *265*, 974–980.

60. Camacho, J.A., Obie, C., Biery, B., Goodman, B.K., Hu, C.A., Almashanu, S., Steel, G., Casey, R., Lambert, M., Mitchell, G.A., et al. (1999). Hyperornithinaemia-hyperammonaemia-homocitrullinuria syndrome is caused by mutations in a gene encoding a mitochondrial ornithine transporter. Nat. Genet. *22*, 151–158.

61. Shih, V.E., Laframboise, R., Mandell, R., and Pichette, J. (1992). Neonatal form of the hyperornithinaemia, hyperammonaemia, and homocitrullinuria (HHH) syndrome and prenatal diagnosis. Prenat. Diagn. *12*, 717–723.

62. Matsuzaka, Y., Tounai, K., Denda, A., Tomizawa, M., Makino, S., Okamoto, K., Keicho, N., Oka, A., Kulski, J.K., Tamiya, G., et al. (2002). Identification of novel candidate genes in the diffuse panbronchiolitis critical region of the class I human MHC. Immunogenetics *54*, 301–309.

63. Di Angelantonio, E., Thompson, S.G., Kaptoge, S., Moore, C., Walker, M., Armitage, J., Ouwehand, W.H., Roberts, D.J., Danesh, J., and INTERVAL Trial Group (2017). Efficiency and safety of varying the frequency of whole blood donation (INTERVAL): a randomised trial of 45 000 donors. Lancet *390*, 2360–2371.
